# Enantioselective Synthesis of 3,5,6‐Substituted Dihydropyranones and Dihydropyridinones using Isothiourea‐Mediated Catalysis

**DOI:** 10.1002/asia.201500907

**Published:** 2015-11-12

**Authors:** Daniel G. Stark, Louis C. Morrill, David B. Cordes, Alexandra M. Z. Slawin, Timothy J. C. O'Riordan, Andrew D. Smith

**Affiliations:** ^1^ School of ChemistryUniversity of St AndrewsNorth HaughSt Andrews FifeKY16 9STUK; ^2^ Syngenta, Jealott's Hill International Research Centre, BracknellBerkshireRG42 6EYUK

**Keywords:** dihydropyranones, dihydropyridinones, enantioselective catalysis, isothioureas, Michael addition

## Abstract

The scope of dihydropyranone and dihydropyridinone products accessible by isothiourea‐catalyzed processes has been expanded and explored through the use of 2‐*N*‐tosyliminoacrylates and 2‐aroylacrylates in a Michael addition‐lactonization/lactamization cascade reaction. Notably, to ensure reproducibility it is essential to use homoanhydrides as ammonium enolate precursors with 2‐aroyl acrylates, while carboxylic acids can be used with 2‐*N*‐tosyliminoacrylates, delivering a range of 3,5,6‐substituted dihydropyranones and dihydropyridinones with high enantioselectivity (typically >90 % *ee*). The derivatization of the heterocyclic core of a 3,5,6‐substituted dihydropyranone through hydrogenation is also reported.

## Introduction

**Figure 1:**
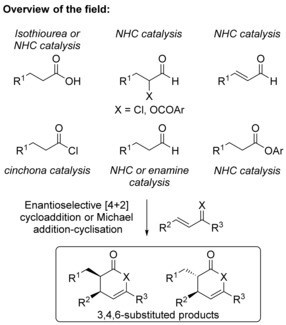
Enantioselective synthesis of dihydropyranones and dihydropyridinones – overview of the field.

The synthesis of small, functionalized chiral heterocycles through asymmetric catalysis remains a prominent area of research in synthetic methodology. The recognition of endocyclic enol dihydropyranones and dihydropyridinones as key constituents within natural products and bioactive compounds aids the appeal of catalytic routes to produce these molecules.^1^ Classical routes involve uncatalyzed Diels–Alder reactions and more recently metal‐catalyzed π‐olefin and π‐alkyne cyclizations.^2^ However, the current state‐of‐the‐art methods for the production of chiral dihydropyranones and dihydropyridinones with high diastereo‐ and enantiocontrol remains the use of organocatalytically generated enolate equivalents. Typical processes within this area have utilized N‐heterocyclic carbene (NHC)‐generated azolium enolates,^3^ enamine catalysis,^4^ or cinchona alkaloids^5^ and isothiourea‐generated ammonium enolates in formal [4+2]‐cycloaddition/Michael addition‐cyclization reactions with electron‐deficient olefins using a wide range of enolate precursors and strategies.^6^ To date, intermolecular reactions within these systems have typically utilized β‐substituted electron‐deficient enones or α,β‐unsaturated ketimines to form 3,4,6‐substituted dihydropyranones and dihydropyridinones with excellent diastereo‐ and enantiocontrol (typically >90:10 d.r., >95 % *ee*; Figure 1).

**Figure 2:**
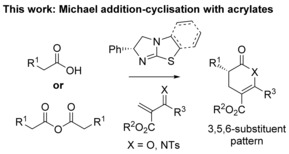
Enantioselective synthesis of dihydropyranones and dihydropyridinones – this work.

Within this area, following pioneering work by Romo and co‐workers,^7^ our laboratory has developed an isothiourea‐catalyzed^8^ Michael‐addition cyclization protocol using bench stable carboxylic acids as starting materials that has been applied to the synthesis of a variety of heterocycles.^9^ Following our previous report on the synthesis of di‐, tri‐, and tetra‐substituted pyridines using 2‐*N*‐tosyliminoacrylates, we considered using β‐unsubstituted Michael acceptors (2‐*N*‐tosyliminoacrylates and 2‐aroylacrylates) in enantioselective isothiourea‐catalyzed Michael addition‐cyclization cascades to generate 3,5,6‐trisubstituted dihydropyranones and dihydropyridinones containing a single stereocenter (Figure 2).^10^ To the best of our knowledge, only limited precedent with such β‐unsubstituted acrylate acceptors in organocatalytic [4+2]‐cycloaddition type processes have been reported.^11^

At the onset of these investigations the highly reactive nature of β‐unsubstituted 2‐*N*‐tosylimino‐ and 2‐aroylacrylates, and the assumed relative rate of a competitive racemic base‐promoted background reaction, were envisioned as problems to overcome to obtain high enantioselectivity in these reactions. In this study, the scope and limitations of this approach are investigated and explored, with the key finding being the necessity to use homoanhydrides as ammonium enolate precursors with 2‐aroylacrylates, while carboxylic acids can be used with 2‐*N*‐tosyliminoacrylates.

## Results and Discussion

### Michael Addition‐Lactonization using 2‐Aroylacrylates; Optimization and Generality

**Scheme 1:**
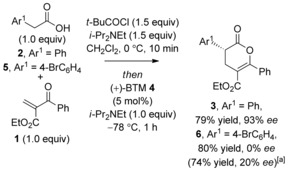
Initial results of the Michael addition‐lactonization. [a] Syringe pump addition of **1** (0.25 m in CH_2_Cl_2_) over 2 h.

Preliminary studies began with optimization of the isothiourea‐catalyzed Michael addition‐lactonization using 2‐aroylacrylate **1** and phenylacetic acid **2** as a model system.^12^ Employing in situ mixed anhydride formation using pivaloyl chloride and phenylacetic acid **2** with 2‐aroylacrylate **1** catalyzed by (+)‐BTM (benzotetramisole) **4** (5 mol %) at −78 °C after 1 h afforded dihydropyranone **3** in an excellent yield of 79 % and 93 % *ee* (Scheme 1). However, disappointingly this method did not prove general when applied to subsequent substrates. In all cases the desired dihydropyranone products were formed in typically excellent yield but with no enantiocontrol. For example, treatment of 4‐bromophenyl acetic acid **5** and 2‐aroylacrylate **1** with *i*Pr_2_NEt, pivaloyl chloride, and (+)‐BTM **4** at −78 °C gave the desired dihydropyranone **6** in a good yield of 80 % but with 0 % *ee*. Attempted optimization through syringe pump addition of Michael acceptor **1** (0.25 m in CH_2_Cl_2_) gave marginal improvement with typical enantioselectivities of approximately 20 % *ee* observed with isothiourea catalyst **4** (Scheme 1).

**Scheme 2:**
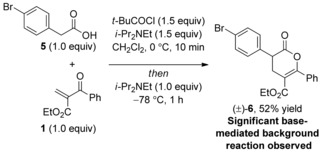
Control experiment.

To investigate the lack of enantiocontrol using 4‐bromophenyl acetic acid **5** in this process the feasibility of a competitive base‐mediated racemic reaction process was probed. Direct treatment of 4‐bromophenyl acetic acid **5** with pivaloyl chloride and *i*Pr_2_NEt, followed by addition of 2‐aroylacrylate **1** without the inclusion of the isothiourea Lewis base yielded dihydropyranone **6** in an isolated yield of 52 %. This is consistent with a significant base‐mediated reaction under these conditions presumably enhanced by the high reactivity of the 2‐aroylacrylate (Scheme 2).^13^

Alternative reaction conditions were explored to prepare the target products with reproducibly high enantioselectivity. We have previously demonstrated the use of homoanhydrides as alternative ammonium enolate precursors to carboxylic acids, with one advantage of this approach being the reduced levels of added organic base necessary for catalysis in comparison to the in situ mixed anhydride approach.9g The use of homoanhydride **7** as the enolate precursor offered a major breakthrough in this system, delivering products with high and reproducible levels of enantioselectivity under optimized reaction conditions (Table 1).

**Table 1:** Optimization studies of the Michael addition‐lactonization.

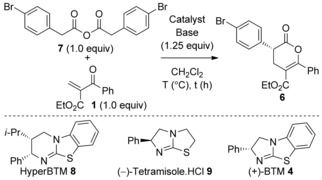						
Entry	Lewis base [mol %]	*T* [°C]	*t* [h]	Base	Yield [%]^[a]^	*ee* [%]^[b]^
1	**8** (5)	rt	0.2	*i*Pr_2_NEt	81	46
2	**9** (5)	rt	0.2	*i*Pr_2_NEt	80	78^[c]^
3	**4** (5)	rt	0.2	*i*Pr_2_NEt	88	88
4	**4** (5)	−78	1	*i*Pr_2_NEt	88	91
5	**4** (5)	−78	1	Et_3_N	85	0
6	**4** (5)	−78	1	Cs_2_CO_3_	58	57
7	**4** (5)	−78	1	Na_2_CO_3_	81	83
8^[d]^	**4** (5)	−78	2	*i*Pr_2_NEt	88	70

[a] Yield of isolated product. [b] Determined by chiral HPLC analysis. [c] (*S*)‐enantiomer obtained. [d] Syringe pump addition of **1** (0.25 m in CH_2_Cl_2_) over 2 h.

Homoanhydride **7** and 2‐aroylacrylate **1** were used as a model system for reaction optimization. Lewis base screening showed 5 mol % (+)‐BTM **4** to be optimal, forming product **6** in 88 % yield and excellent 91 % *ee* at −78 °C (Table 1, entry 4). The nature of the base used in this process also proved key to high enantioselectivity, as Et_3_N gave the product in 85 % yield, but in racemic form, consistent with a competitive base‐catalyzed background reaction (Table 1, entry 5). The inorganic base Cs_2_CO_3_ gave **6** in poor yield and *ee*, whereas Na_2_CO_3_ proved moderately successful, and yielded **6** in 81 % yield and 83 % *ee* (Table 1, entries 6 and 7). Syringe pump‐addition of Michael acceptor **1** in CH_2_Cl_2_ over 2 h gave no improvement, and formed **6** in 70 % *ee* (Table 1, entry 8).

**Figure 3:**
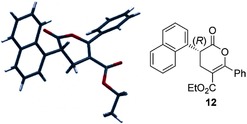
Molecular representation of X‐ray structure **12**.

With optimized reaction conditions in hand, the scope of this reaction process was evaluated (Table 2).^14^ The use of a homoanhydride containing an electron‐donating 4‐MeOC_6_H_4_ substituent was tolerated and gave **10** in 83 % yield and 91 % *ee*, while 4‐substituted halogenated aromatics could also be installed in high yield and *ee* (products **6** and **11**). Pleasingly, sterically demanding enolate precursors such as the 1‐naphthyl and *o*‐tolyl homoanhydrides could be applied, producing **12** in an excellent yield of 81 % and 97 % *ee* and **13** in 81 % yield with moderate 50 % *ee*. Assessing the scope of aroyl acrylate Michael acceptors, the electron‐rich 4‐MeOC_6_H_4_ aryl unit was incorporated in **14** and isolated in 79 % yield and 91 % *ee*. Dihydropyranone **15** was synthesized in 65 % yield and 81 % *ee*, however, in this case Na_2_CO_3_ was required as the base to obtain good enantioselectivity.^15^ Furthermore, 4‐methoxyphenyl acetic anhydride was explored with heteroaryl groups such as 2‐furyl, and gave **16** in 61 % yield and 99 % *ee*. Halogenated aromatics can be installed at the 6‐position to afford **17** in 61 % yield but with moderate 68 % *ee*. Finally, the naphthyl substituent was employed and afforded **18** in 71 % yield and 86 % *ee*. The absolute configuration of **12** was confirmed by X‐ray diffraction with all other examples assigned by analogy (Figure 3).^16^

**Table 2:** Substrate scope of the Michael addition‐lactonization.

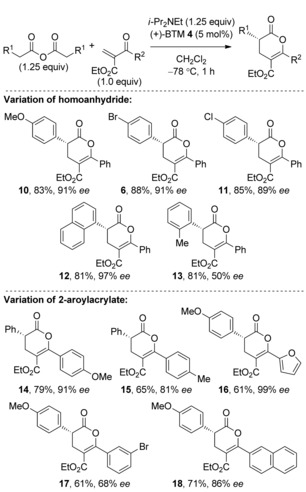

**Scheme 3:**
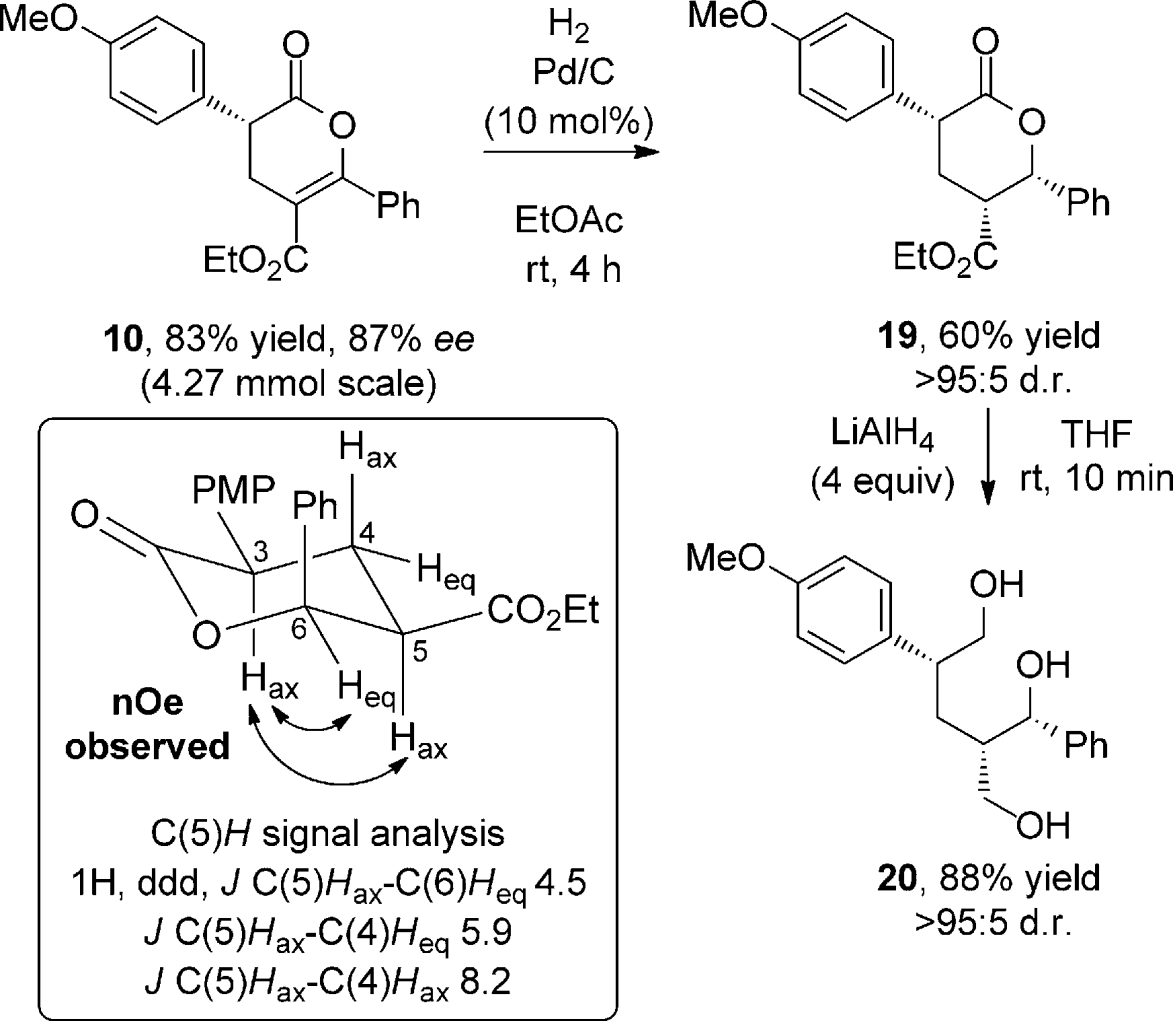
Derivatization of dihydropyranone **10**.

Following the development of this procedure for the synthesis of 3,5,6‐substituted dihydropyranones, the utility and further elaboration of these products was explored. The isothiourea‐catalyzed Michael addition‐lactonization could be readily carried out on a reasonable laboratory scale (4.27 mmol), thereby providing 1.27 g of **10** in 87 % *ee*. Dihydropyranone **10** could be transformed into pyranone **19** through a substrate controlled Pd/C‐catalyzed hydrogenation to afford product **19** in 60 % yield with >95:5 d.r. (Scheme 3). Pyranone **19** was further derivatized by a reductive ring‐opening, providing triol **20** in 88 % yield and high diastereoselectivity. Nuclear Overhauser effect (NOE) experiments confirmed the relative configuration of **19**, however, the *ee* determination of **19** or triol **20** by chiral HPLC or GC was not possible in our hands.^17^

### Michael Addition‐Lactamization using 2‐*N*‐tosyliminoacrylates; Optimization and Generality

Following the successful synthesis of dihydropyranones through an isothiourea‐catalyzed Michael addition‐lactonization process, the method was extended to explore the structurally related dihydropyridinone motif. Initial investigations began with optimization using 2‐*N*‐tosyliminoacrylate **21** and carboxylic acid **2**. Treatment of **2** with pivaloyl chloride and *i*Pr_2_NEt followed by subsequent addition of Michael acceptor **21** and HyperBTM **8** (10 mol %) in CH_2_Cl_2_ at room temperature gave dihydropyridinone **22** in 60 % yield and 80 % *ee* after 30 min (Table 3, entry 1). A screen of isothiourea catalysts revealed (−)‐tetramisole **9** to be optimum, providing dihydropyridinone **22** in 62 % yield and 84 % *ee* (Table 3, entry 2). Lowering the reaction temperature to −78 °C gave **22** in a 74 % yield and an improved 91 % *ee* (Table 3, entry 5). The reaction was sensitive to solvent choice in terms of both conversion and enantioselectivity. For example, THF gives a poor 40 % yield of **22** in 86 % *ee* (Table 3, entry 7), while MeCN at −30 °C provides **22** in 70 % yield but in racemic form (Table 3, entry 8). Finally, the catalyst loading for **9** could be lowered to 5 mol % without compromising the yield or *ee* (Table 3, entry 9).

**Table 3:** Optimization studies of the Michael addition‐lactamization.

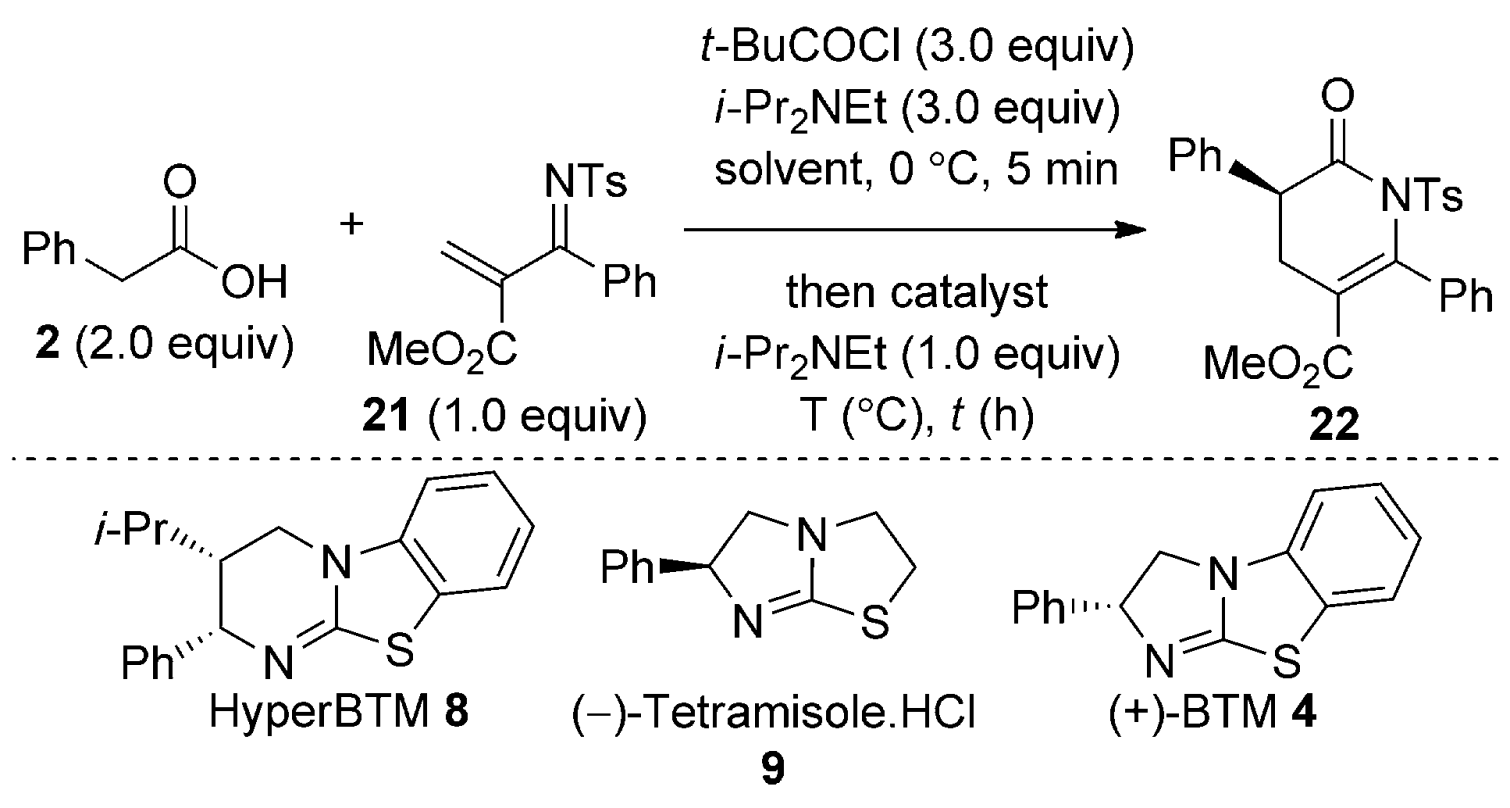						
Entry	Lewis base [mol %]	*T* [°C]	*t* [h]	Solvent	Yield [%]^[a]^	*ee* [%]^[b]^
1	**8** (10)	rt	0.5	CH_2_Cl_2_	60	80^[c]^
2	**9** (10)	rt	0.5	CH_2_Cl_2_	62	84
3	**4** (10)	rt	0.5	CH_2_Cl_2_	60	80^[c]^
4	**8** (10)	−78	16	CH_2_Cl_2_	71	86^[c]^
5	**9** (10)	−78	16	CH_2_Cl_2_	74	91
6	**4** (10)	−78	16	CH_2_Cl_2_	70	85^[c]^
7	**9** (10)	−78	16	THF	40	86
8	**9** (10)	−30	16	MeCN	70	0
9	**9** (5)	−78	16	CH_2_Cl_2_	74	91

[a] Yield of isolated product. [b] Determined by chiral HPLC analysis. [c] (*R*)‐enantiomer obtained.

**Figure 4:**
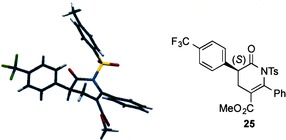
Molecular representation of X‐ray structure **25**.

The generality of this process was next examined using (−)‐tetramisole **9** (5 mol %) as the Lewis base. Initially 2‐*N*‐tosyliminoacrylate **21** was treated with a range of commercially available carboxylic acids under the previously optimized reaction conditions (Table 4). However, some examples gave poor conversion of the desired dihydropyridinone products even after extended reaction times at −78 °C. Therefore, a more general procedure was developed by allowing the reaction to warm over 16 h from −78 °C to room temperature, giving full conversion of the Michael acceptor with a range of acetic acids.^18^ Electron‐rich aromatic substituents such as 4‐MeOC_6_H_4_ and 4‐Me_2_NC_6_H_4_ are tolerated and desired products **23** and **24** are afforded in good yields of 76 % and 71 % with excellent 95 % and 94 % *ee*, respectively. Electron‐deficient aryl units were also tolerated, and the CF_3_‐bearing dihydropyridinone **25** was isolated in 73 % yield and 93 % *ee*. 3‐Tolylacetic acid produced dihydropyridinone **26** in 69 % yield and high 95 % *ee*. Halogen‐substituted aryl substituents could also be incorporated, giving product **27** in 61 % yield, albeit a reduced 71 % *ee* was obtained. Pleasingly, heteroaryl groups were tolerated and delivered dihydropyridinone **28** in 65 % yield and excellent 90 % *ee*. Next, the scope of 2‐*N*‐tosyliminoacrylate Michael acceptors was explored. Unfortunately, only electron‐rich aryl units could be included at the 6‐position of the dihydropyridinone products owing to a limitation in the synthesis of the 2‐*N*‐tosyliminoacrylates.^19^ For example, the 4‐MeOC_6_H_4_ aryl group could be included to give product **29** in 63 % yield and 98 % *ee*. Also, 4‐tolyl and 3,5‐xylyl groups were well tolerated and afforded the corresponding products **30** and **31** in 60 % yield, 97 % *ee* and 59 % yield and 90 % *ee*, respectively. Finally, 2‐naphthyl substitution was possible, **32** was formed in 69 % yield and 91 % *ee*. This Michael addition‐lactamization method was also performed on reasonable laboratory scale (4.17 mmol), thus providing 1.30 g of **22** with excellent 99 % *ee*. The absolute configuration of **25** was determined by X‐ray diffraction, with all other products assigned by analogy (Figure 4).^20^

**Table 4:** Substrate scope of the Michael addition‐lactamization.

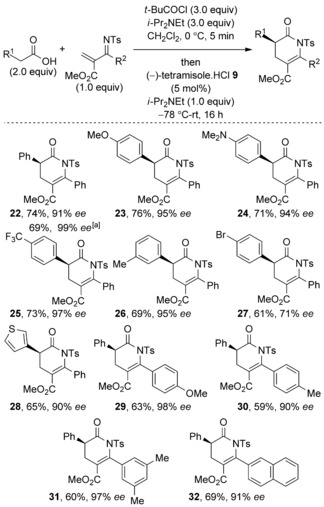

[a] 4.17 mmol scale.

**Figure 5:**
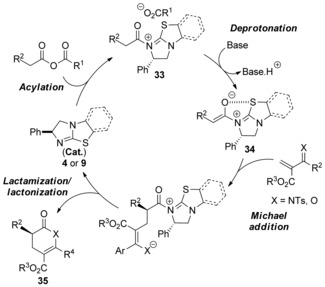
Proposed catalytic cycle.

Following our previous studies a proposed mechanism for the processes described above begins with *N*‐acylation of isothiourea catalyst with either the homoanhydride (with aroyl acrylates) or in situ formed mixed anhydride (with imino acrylates) to form an acyl ammonium species **33** (Figure 5). Subsequent deprotonation gives (*Z*)‐ammonium enolate **34**, which is stabilized by a proposed *n*_O_ to σ*_C−S_ interaction or favorable electrostatic stabilization between the enolate oxygen and sulfur atom on the catalyst framework.^21^ Enantioselective Michael addition to an aroyl acrylate or imino acrylate, followed by lactamization/lactonization, provides the corresponding heterocyclic products **35** and releases the catalyst.

## Conclusions

In conclusion, the isothiourea‐catalyzed Michael‐addition lactamization/lactonization of 2‐[aryl(tosylimino)methyl]acrylate or 2‐aroylacrylates from arylacetic acids or homoanhydrides, respectively, produces stereodefined 3,5,6‐substituted dihydropyridinones or dihydropyranones in high yield and enantioselectivity. Using these products to provide further complex chiral building blocks has been demonstrated through the use of hydrogenation or ring‐opening processes. Further studies within our laboratory are focused towards the continued development of isothioureas and other Lewis bases in catalysis.

## Experimental Section

**General procedure**: Isothiourea‐catalyzed Michael addition‐lactonization

To a solution of requisite homoanhydride (1.25 equiv) in CH_2_Cl_2_ (0.31 m in homoanhydride) at −78 °C was added Lewis base catalyst (5 mol %) and the reaction stirred for 20 min. A solution of Michael acceptor (1.0 equiv) in CH_2_Cl_2_ (0.25 m), pre‐cooled to −78 °C, is added followed by a solution of *i*Pr_2_NEt (1.25 equiv) in CH_2_Cl_2_ (0.31 m), also pre‐cooled to −78 °C, and reaction stirred until complete by TLC analysis. The reaction was quenched with HCl (1 m in H_2_O), extracted with CH_2_Cl_2_ (×3), dried over MgSO_4_, and concentrated under reduced pressure to give the crude residue. Products were purified by Biotage Isolera 4 and kieselgel 60 (0.040–0.063 mm) silica grade in the solvent system reported.

**General procedure**: Isothiourea‐catalyzed Michael addition‐lactamization

To a solution of requisite carboxylic acid (2.0 equiv) in CH_2_Cl_2_ (0.1 m in carboxylic acid) at 0 °C was added *i*Pr_2_NEt (3.0 equiv) and pivaloyl chloride (3.0 equiv). The reaction was left to stir for 10 min before being cooled to −78 °C at which point Lewis base catalyst (5 mol %), Michael acceptor (1.0 equiv), and *i*Pr_2_NEt (1.0 equiv) were added and the reaction was warmed to room temperature over 16 h. The reaction was quenched with HCl (1 m in H_2_O), extracted with CH_2_Cl_2_ (×3), dried over MgSO_4_, and concentrated under reduced pressure to give crude residue. Products were purified by column chromatography in the solvent system reported.

The data underpinning the work in this manuscript can be found at http://dx.doi.org/10.17630/6e0ad60a‐ddf5‐459c‐bc45‐cdd80fd518b8

## Supporting information

As a service to our authors and readers, this journal provides supporting information supplied by the authors. Such materials are peer reviewed and may be re‐organized for online delivery, but are not copy‐edited or typeset. Technical support issues arising from supporting information (other than missing files) should be addressed to the authors.

Supplementary
