# Supplementary material for: Enantioselective Synthesis of 3,5,6‐Substituted Dihydropyranones and Dihydropyridinones using Isothiourea‐Mediated Catalysis
Source: Chem Asian J. 2015 Nov 12;11(3):395–400. doi: 10.1002/asia.201500907 (PMC4755233; doi:10.1002/asia.201500907)
Supplement: Supplementary file 1 — Supplementary [file ASIA-11-395-s001.pdf]

CHEMISTRY
AN ASIAN JOURNAL

Supporting Information

Enantioselective Synthesis of 3,5,6-Substituted Dihydropyranones and Dihydropyridinones using Isothiourea-Mediated Catalysis

Daniel G. Stark,[a] Louis C. Morrill,[a] David B. Cordes,[a] Alexandra M. Z. Slawin,[a] Timothy J. C. O'Riordan,[b] and Andrew D. Smith*[a]

asia_201500907_sm_miscellaneous_information.pdf

Contents

1.0 General Information 2

2.0 Isothiourea-Catalysed Michael Addition-Lactonisation 3

Preparation of homoanhydrides 3

Preparation of keto esters 5

Preparation of aroyl acrylates 7

Isothiourea-catalysed Michael addition-lactonisation 10

Derivatisation of dihydropyranone 10 18

Determination of Relative Stereochemistry for 19 19

3.0 Isothiourea-Catalysed Michael Addition-Lactamisation 20

Preparation of 2-(aryl(tosylimino)methyl)acrylates 20

Isothiourea-catalysed Michael addition-lactamisation 22

4.0 ^{1}H NMR and $\{^{1}H\}^{13}C$ NMR spectra for novel compounds 31

5.0 Chiral HPLC analysis 78

1.0 General Information

Reactions were performed in flame-dried glassware under an Ar or N_2 atmosphere unless otherwise stated. Anhydrous CH_2Cl_2, Et_2O, THF and toluene were obtained from an MBraun SPS-800 system. Petrol is defined as petroleum ether 40–60 °C. All other solvents and commercial reagents were used as received without further purification unless otherwise stated. Room temperature (rt) refers to 20–25 °C. Temperatures of 0 °C and −78 °C were obtained using ice/water and CO_2(s)/acetone baths respectively.

Analytical thin layer chromatography was performed on pre-coated aluminium plates (Kieselgel 60 F_{254} silica). Plates were visualised under UV light (254 nm) or by staining with either phosphomolybdic acid or $KMnO_4$ followed by heating. Flash column chromatography was performed on Kieselgel 60 silica in the solvent system stated under a positive pressure of compressed air or on a Biotage® IsoleraTM 4, using Biotage® Snap Ultra or Biotage® KP Sil columns under the solvent system stated.

Melting points were recorded on an Electrothermal 9100 melting point apparatus. Optical rotations were measured on a Perkin Elmer Precisely/Model-341 polarimeter operating at the sodium D line with a 100 mm path cell at 20 °C.

HPLC analyses were obtained on a Shimadzu HPLC consisting of a DGU-20A5 degasser, LC-20AT liquid chromatography SIL-20AHT autosampler, CMB-20A communications bus module, SPDM20A diode array detector and a CTO-20A column oven that allows the temperature to be set from 25–40 °C. Separation was achieved using Chiralcel columns.

Infrared spectra (ν_{max}) were recorded on a Shimadzu IRAffinity-1 Fourier transform IR spectrophotometer using either thin film or solid using Pike MIRacle ATR accessory. Analysis was carried out using Shimadzu IRsolution v1.50 and only characteristic peaks are reported.

^{1}H, ^{13}C{^{1}H}, and ^{19}F{^{1}H} NMR spectra were recorded on Bruker Avance 500 MHz, Bruker Avance 400 MHz and Bruker Avance 300 MHz NMR spectrometers. In $CDCl_3$, ^{1}H and ^{13}C{^{1}H} NMR chemical shifts are reported relative to $CHCl_3$ at 7.27 ppm and 77.0 ppm, respectively. Coupling constants (*J*) are reported in Hertz (Hz). Multiplicities are indicated by: br s (broad singlet), s (singlet), d (doublet), t (triplet), q (quartet) and m (multiplet).

Mass spectrometry (*m/z*) data were acquired by electrospray ionisation (ES) or nanospray ionisation (NSI) at the EPSRC UK National Mass Spectrometry Facility at Swansea University.

2.0 Isothiourea-Catalysed Michael Addition-Lactonisation

Preparation of Homoanhydrides

General Procedure A: Preparation of Homoanhydrides

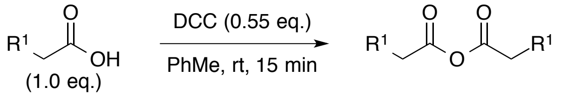

To a solution of requisite carboxylic acid (1.0 equiv) in toluene (0.37 M in acid) at rt was added DCC (0.55 equiv) and the reaction stirred for 15 min. The reaction was filtered through Celite (eluent: toluene) and concentrated under reduced pressure to give crude reaction mixture. Products were purified by recrystallisation if required.

2-Phenylacetic anhydride

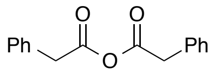

2

Following general procedure A, phenylacetic acid (1.00 g, 7.34 mmol) and DCC (757 mg, 3.67 mmol) in toluene (20 mL) gave crude reaction mixture. Recrystallisation (Et_2O) gave title compound as white solid (1.70 g, 91%); mp 70-72 °C; {lit.[1] mp 72-72.5 °C}; ^{1}H NMR (500 MHz, $CDCl_3$) δ_H: 3.76 (4H, s, C*H*$_2$), 7.23–7.25 (4H, m, Ar(3,5)*H*), 7.32–7.38 (6H, m, Ar(4)*H* and Ar(2,6)*H*). Spectroscopic data are in accordance with the literature.[1]

2-(4-Methoxyphenyl)acetic anhydride

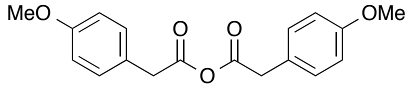

36

Following general procedure A, 4-methoxyphenylacetic acid (1.00 g, 6.00 mmol) and DCC (681 mg, 3.30 mmol) in toluene (25 mL) gave crude reaction mixture. Recrystallisation (Et_2O) gave title compound as white solid (1.70 g, 90%); mp 61-62 °C; {lit.[1] mp 60-62 °C}; ^{1}H NMR (500 MHz, $CDCl_3$) δ_H: 3.66 (4H, s, C*H*$_2$), 3.80 (4H, s, C*H*$_2$), 6.83–6.86 (4H, m, Ar(3,5)*H*), 7.10–7.13 (4H, m, Ar(2,6)*H*). All data are in accordance with the literature.[1]

2-(4-Bromophenyl)acetic anhydride

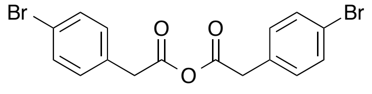

5

Following general procedure A, 4-bromophenylacetic acid (1.00 g, 4.65 mmol) and DCC (528 mg, 2.56 mmol) in toluene (20 mL) gave crude reaction mixture. Recrystallisation (Et_2O) gave title compound as white solid (0.97 g, 97%); mp 75-77 °C; {lit.[2] mp 76-78 °C}; ^{1}H NMR (300 MHz, $CDCl_3$) δ_H: 3.61 (4H, s, CH_2), 6.98–7.03 (4H, m, Ar(3,5)*H*), 7.36–7.41 (4H, m, Ar(2,6)*H*). All data are in accordance with the literature.[2]

2-(4-Chlorophenyl)acetic anhydride

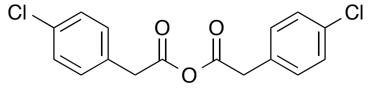

37

Following general procedure A, 4-chlorophenylacetic acid (1.00 g, 5.90 mmol) and DCC (669 mg, 3.25 mmol) in toluene (50 mL) gave crude reaction mixture. Recrystallisation (Et_2O) gave title compound as white solid (0.94 g, 98%); mp 62-64 °C; {lit.[1] mp 62-64 °C}; ^{1}H NMR (500 MHz, $CDCl_3$) δ_H: 3.70 (4H, s, CH_2), 7.13 (4H, d, *J* 8.5, Ar(3,5)*H*), 7.28–7.31 (4H, m, Ar(2,6)*H*). All data are in accordance with the literature.[1]

2-(Naphthalen-1-yl)acetic anhydride

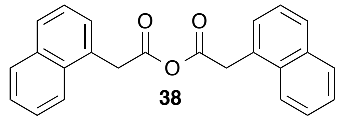

38

Following general procedure A, 1-naphthylacetic acid (2.00 g, 10.8 mmol) and DCC (1.23 g, 5.94 mmol) in toluene (50 mL) gave crude reaction mixture. Recrystallisation (Et_2O) gave title compound as white solid (2.26 g, 59%); mp 34-36 °C; {lit.[3] mp 36-38 °C}; ^{1}H NMR (400 MHz, $CDCl_3$) δ_H: 4.30 (4H, C(2)*H*), 7.23 (2H, d, *J* 6.9, Ar(1)*H*), 7.33-7.36 (2H, m, Ar(8)*H*), 7.47-7.50 (6H, m, Ar*H*), 7.79-7.82 (4H, m, Ar*H*), 7.86-7.87 (2H, m, Ar*H*). All data are in accordance with the literature.[3]

2-(2-Tolyl)acetic anhydride

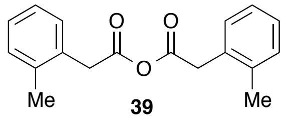

39

Following general procedure A, 2-tolylphenylacetic acid (2.00 g, 16.6 mmol) and DCC (1.88 mg, 9.13 mmol) in toluene (70 mL) gave crude reaction mixture as yellow oil that was used

immediately without further purification. ^{1}H NMR (400 MHz, $CDCl_3$) δ_H: 2.74 (6H, s, ArCH_3), 3.72 (4H, s, C(2)*H*), 7.09-7.21 (8H, m, Ar*H*). Compound was **39** used immediately without further purification or analysis due to instability.

Preparation of keto esters

General Procedure B: Preparation of keto esters

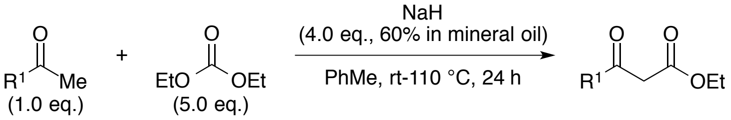

Following the literature procedure of Bretner *et al*,[4] to a solution of ketone in toluene (0.42 M in ketone) at 0 °C was added NaH (60% w/w in mineral oil, 4 equiv) and reaction warmed to rt and stirred for 2 h. Reaction mixture was added dropwise to a solution of diethyl carbonate (5 equiv) in toluene ([6.3 M in carbonate]) and reaction stirred at reflux for 24 h. Once cooled the reaction mixture was quenched slowly with H_2O, acidified with HCl (2 M in H_2O) and extracted with EtOAc (×3). The combined organic fractions were dried over $MgSO_4$, filtered and concentrated under reduced pressure. Products were purified by column chromatography in the solvent system reported.

Ethyl 3-(4-methoxyphenyl)-3-oxopropanoate

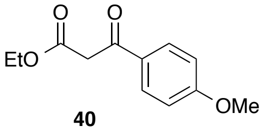

40

Following general procedure B, 4-methoxyacetophenone (5.00 g, 33.3 mmol) in toluene (79 mL), NaH (3.19 g, 133 mmol), diethyl carbonate (20 mL, 166 mmol) in toluene (25 mL) gave crude product. Column chromatography (10:90 EtOAc:hexane, R_f = 0.26) gave the title compound as a yellow oil as a (5.55 g, 75%, 2.5:1 keto:enol tautomeric mixture); ^{1}H NMR (500 MHz, $CDCl_3$) δ_H: 1.26 (3H, t, *J* 7.1, keto $CO_2CH_2CH_3$), 1.33 (1.2H, t, *J* 7.1, enol $CO_2CH_2CH_3$), 3.85 (1.2H, s, enol ArOCH_3), 3.88 (3H, s, keto ArOCH_3), 3.94 (2H, s, keto C(2)*H*), 4.17-4.29 (2.8H, m, keto $CO_2CH_2CH_3$ and enol $CO_2CH_2CH_3$), 5.58 (0.4H, s, enol C(2)*H*), 6.91-6.96 (2.8H, m, keto Ar(3,5)*H* and enol Ar(3,5)*H*), 7.74 (0.8H, d, *J* 7.7, enol Ar(2,6)*H*), 7.93 (2H, d, *J* 9.0, keto Ar(2,6)*H*), 12.6 (0.4H, s, enol O*H*). All data in accordance with literature.[5]

Ethyl 3-oxo-3-(*p*-tolyl)propanoate

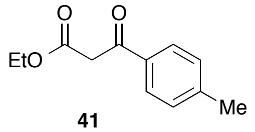

41

Following general procedure B, 4-methylacetophenone (2.97 mL, 22.4 mmol) in toluene (53 mL), NaH (3.58 g, 89.6 mmol), diethyl carbonate (13.6 mL, 112 mmol) in toluene (18 mL) gave crude product. Column chromatography (7.5:92.5 EtOAc:hexane, R_f = 0.25) gave the title compound as a yellow oil as a (6.02 g, 78%); ^{1}H NMR (500 MHz, $CDCl_3$) δ_H: 1.27 (3H, t, *J* 7.1, $CO_2CH_2CH_3$), 2.42 ($ArCH_3$), 3.96 (2H, s, C(2)*H*), 4.21 (2H, q, *J* 7.2, $CO_2CH_2CH_3$), 7.28 (2H, d, *J* 8.0, Ar(3,5)*H*), 7.85 (2H, d, *J* 8.3, Ar(2,6)*H*). All data in accordance with literature.[5]

Ethyl 3-(furan-2-yl)-3-oxopropanoate

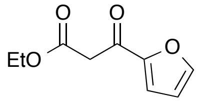

42

To a solution of 2-acetlyfuran (3.00 g, 27.2 mmol) in diethyl carbonate (54.4 mL) was added NaH (2.72 g, 68.0 m mmol) and the reaction stirred at rt for 1 h. Reaction was then heated to 90 C and stirred for further 2 h. The reaction was cooled and quenched with H_2O, acidified to pH 5 and extracted with EtOAc (×3). Combined organics were washed with brine, dried over $MgSO_4$ and concentrated under reduced pressure to give crude mixture. Column chromatography (10:90, EtOAc:hexane R_f = 0.25) gave the title compound as a brown oil as a (2.43 g, 49%, 2:1 keto:enol tautomeric mixture); ^{1}H NMR (500 MHz, $CDCl_3$) δ_H: 1.25 (3H, t, *J* 7.1, $CO_2CH_2CH_3$), 3.84 (2H, s, C(2)H_2), 4.20 (2H, q, *J* 7.1, $CO_2CH_2CH_3$), 6.57 (1H, dd, *J* 3.6, 1.6, Ar(4)*H*), 7.27 (1H, d, *J* 3.59, Ar(3)*H*), 7.61 (1H, s, Ar(5)*H*). All data in accordance with literature.[7]

Ethyl 3-(3-bromophenyl)-3-oxopropanoate

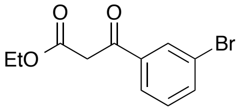

42

Following general procedure B, 4-bromoacetophenone (3.00 g, 15.1 mmol) in toluene (36 mL), NaH (1.45 g, 60.4 mmol), diethyl carbonate (9.16 mL, 75.5 mmol) in toluene (12 mL) gave crude product. Column chromatography (7.5:92.5 EtOAc:hexane, Rf = 0.25) gave the title compound as a yellow oil as a (2.05 g, 50%, 2:1 keto:enol tautomeric mixture); ^{1}H NMR (500 MHz, $CDCl_3$) δ_H: 1.24-1.36 (4.5H, t, *J* 7.1, keto $CO_2CH_2CH_3$ enol $CO_2CH_2CH_3$), 3.96 (2H, s, keto C(2)*H*), 4.18-4.28 (3H, m, keto $CO_2CH_2CH_3$ and enol $CO_2CH_2CH_3$), 5.65 (0.5H,

s, enol C(2)*H*), 7.29-7.40 (1.5H, m, keto Ar(5)*H* and enol Ar(5)*H*), 7.58 (0.5H, ddd, *J* 8.0, 2.0, 1.0, enol Ar(4)*H*), 7.67-7.74 (1.5H, m, keto Ar(4)*H* and enol Ar(6)*H*), 7.86 (1H, dt, *J* 7.8, 1.3, keto Ar(6)*H*), 7.92 (0.5H, t, *J* 1.8, enol Ar(2)*H*), 8.08 (1H, t, *J* 1.8, keto C(2)*H*), 12.5 (0.5H, s, enol O*H*). All data in accordance with literature.[6]

Ethyl 3-(naphthalen-2-yl)-3-oxopropanoate

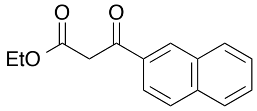

43

Following general procedure B, 4-acetylnaphthalene (3.00 g, 17.6 mmol) in toluene (42 mL), NaH (2.82 g, 70.4 mmol), diethyl carbonate (10.7 mL, 88.1 mmol) in toluene (14 mL) gave crude product. Column chromatography (10:90, EtOAc:hexane R_f = 0.24) gave the title compound as a yellow oil as a (2.56 g, 60%, 4:1 keto:enol tautomeric mixture); ^{1}H NMR (500 MHz, $CDCl_3$) δ_H: 1.27 (3H, t, *J* 7.1, keto $CO_2CH_2CH_3$), 4.13 (2H, s, keto C(2)H_2), 4.24 (2H, q, *J* 7.1, keto $CO_2CH_2CH_3$), 7.56-7.59 (1H, td, *J* 7.6, 7.0, 1.2, keto Ar*H*), 7.61-7.64 (1H, m, keto Ar*H*), 7.88-7.92 (2H, m, keto Ar*H*), 7.98 (1H, d, *J* 8.1, keto Ar*H*), 8.02 (1H, dd, *J* 8.6, 1.8, keto Ar*H*), 8.46 (1H, s, keto Ar*H*). All data in accordance with literature.[7]

Preparation of Aroyl Acrylates

Diisopropylammonium 2,2,2-trifluoroacetate

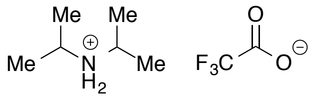

Following a literature procedure,[8] to a solution of DIPA (14.1 mL, 100 mmol) in Et_2O (100 mL) at 0 °C was added TFA (7.70 mL, 100 mmol) dropwise and the reaction mixture was stirred for 5 minutes at 0 °C. The reaction mixture was filtered and the resulting solid was washed with cold Et_2O and dried under reduced pressure to give the title compound (18.4 g, 86%) as a white solid with spectroscopic data in accordance with the literature.[8] mp 120-122 °C {Lit.[9] mp 122-123 °C}; ^{1}H NMR (500 MHz, $CDCl_3$) δ_H: 1.31 (12H, d, *J* 6.7, 4 CH_3), 3.35 (2H, septet, *J* 6.7, 2 C*H*), 8.92 (2H, s, NH_2). All data in accordance with literature.

General Procedure C: Preparation of ethyl aroylacrylates

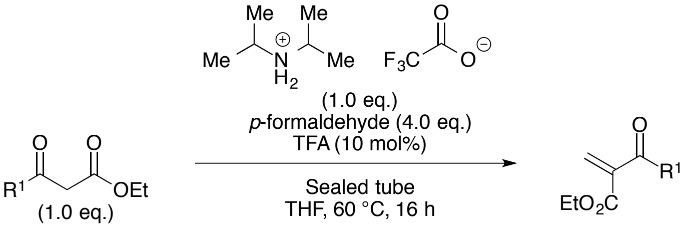

Keto ester (1 equiv), diisopropylammonium 2,2,2-trifluoroacetate (1 equiv), paraformaldehyde (4 equiv) and TFA (0.1 equiv) were added to THF (0.67 M in keto ester) in a sealable reaction tube. The reaction mixture was sealed and heated at reflux for 24 h. Once cooled the reaction mixture was diluted with H_2O and extracted with EtOAc (×3). The combined organic fractions were dried over $MgSO_4$, filtered and concentrated under reduced pressure. Products were purified by column chromatography in the solvent system reported.

Ethyl 2-benzoylacrylate

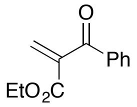

1

Following general procedure C, ethyl benzoylacetate (1.73 mL, 10.0 mmol), diisopropylammonium 2,2,2-trifluoroacetate (2.15 g, 10.0 mmol), paraformaldehyde (1.20 g, 40 mmol) and TFA (77 µL, 1.00 mmol) in THF (60 mL) gave crude product. Column chromatography (10:90 Petrol:EtOAc, R_f = 0.20) to give the title compound (1.80 g, 89%) as a pale yellow oil: ^{1}H NMR (500 MHz, $CDCl_3$) δ_H: 1.19 (3H, t, *J* 7.1, C*H*$_3$), 4.22 (2H, q, *J* 7.1, C*H*$_2$CH$_3$), 6.07 (1H, d, *J* 0.8, =C*H*H), 6.70 (1H, d, *J* 0.8, =CH*H*), 7.47 (2H, t, *J* 7.8, ArC(3,5)H), 7.56-7.65 (1H, m, ArC(4)*H*), 7.86 (2H, d, *J* 8.1, ArC(2,6)*H*). All data in accordance with literature.[10]

Ethyl 2-(4-methoxybenzoyl)acrylate

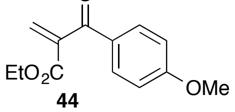

44

Following general procedure C, ethyl 3-(4-methoxyphenyl)-3-oxopropanoate **40** (2.00 mL, 9.43 mmol), diisopropylammonium 2,2,2-trifluoroacetate (2.03 g, 9.43 mmol), paraformaldehyde (1.13 g, 37.7 mmol) and TFA (74 µL, 0.94 mmol) in THF (60 mL) gave crude product. Column chromatography (10:90 Petrol:EtOAc, R_f = 0.21) to give the title compound (1.80 g, 89%) as a yellow oil: ^{1}H NMR (500 MHz, $CDCl_3$) δ_H: 1.22 (3H, t, *J* 7.1, C*H*$_3$), 3.88 (3H, s, ArOC*H*$_3$), 4.23 (2H, q, *J* 7.1, C*H*$_2$CH$_3$), 6.00 (1H, d, *J* 0.7, =C*H*H), 6.66

(1H, d, J 0.7, =CHH), 6.94 (2H, d, J 9.0, ArC(3,5)H), 7.86 (2H, d, J 9.0, ArC(2,6)H). All data in accordance with literature.[10]

Ethyl 2-(4-methylbenzoyl)acrylate

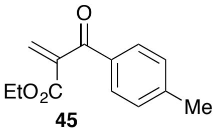

45

Following general procedure C, ethyl 3-(4-tolyl)-3-oxopropanoate **41** (2.0 g, 9.69 mmol), diisopropylammonium 2,2,2-trifluoroacetate (2.08 g, 9.69 mmol), paraformaldehyde (1.17 g, 38.8 mmol) and TFA (75µL, 0.97 mmol) in THF (60 mL) gave crude product. Column chromatography (10:90 Petrol:EtOAc, R_f = 0.24) to give the title compound (1.23 g, 61%) as a yellow oil: ^{1}H NMR (500 MHz, $CDCl_3$) δ_H: 1.21 (3H, t, J 7.1, $CO_2CH_2CH_3$), 4.13 (2H, q, J 7.2, 6.03 (1H, d, J 0.7, =CHH), 6.68 (1H, d, J 0.7, =CHH), 7.26 (2H, d, J 8.2, Ar(3,5)H), 7.77 (2H, d, J 8.2, Ar(2,6)H). All data in accordance with literature.[10]

Ethyl 2-(furan-2-carbonyl)acrylate

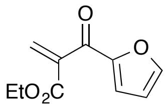

46

Following general procedure C, ethyl 3-(furan-2-yl)-3-oxopropanoate **42** (1.50 g, 8.23 mmol), diisopropylammonium 2,2,2-trifluoroacetate (1.77 g, 8.23 mmol), paraformaldehyde (987 mg, 32.9 mmol) and TFA (92 µL, 0.82 mmol) in THF (60 mL) gave crude product. Column chromatography (15:85 Petrol:EtOAc, R_f = 0.25) to give the title compound (766 g, 48%) as a yellow oil: ^{1}H NMR (400 MHz, $CDCl_3$) δ_H: 1.26 (3H, t, J 7.1, $CO_2CH_2CH_3$), 4.27 (2H, q, J 7.1, $CO_2CH_2CH_3$), 6.21 (1H, d, J 0.7, =CHH), 6.57 (1H, dd, J 3.6, 1.7, Ar(4)H), 6.66 (1H, d, J 0.7, =CHH), 7.20 (1H, dd, J 3.6, 0.8, Ar(3)H), 7.65 (1H, dd, J 1.7, 0.8, Ar(5)H). All data in accordance with literature.[10]

Ethyl 2-(3-bromobenzoyl)acrylate

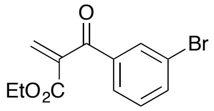

47

Following general procedure C, ethyl 3-(3-bromophenyl)-3-oxopropanoate **42** (2.05 g, 7.56 mmol), diisopropylammonium 2,2,2-trifluoroacetate (1.63 g, 7.56 mmol), paraformaldehyde (906 mg, 30.2 mmol) and TFA (57 µL, 0.76 mmol) in THF (60 mL) gave crude product. Column chromatography (10:90 Petrol:EtOAc, R_f = 0.24) to give the title compound (447 mg, 21%) as a yellow oil: ^{1}H NMR (500 MHz, $CDCl_3$) δ_H: 1.21 (3H, t, J 7.1, CH_3), 4.24 (2H, q, J

7.1, CH_2CH_3), 6.10 (1H, s, =C*H*H), 6.72 (1H, d, *J* 0.7, =CH*H*), 7.35 (1H, t, *J* 7.9, Ar(5)*H*), 7.72 (1H, ddd, *J* 8.0, 1.9, 1.0, Ar(5)*H*), 7.76 (1H, dt, *J* 7.8, 1.3, Ar(2)*H*), 7.99 (1H, t, *J* 1.8, Ar(2)*H*). All data in accordance with literature.[10]

Ethyl 2-(2-naphthoyl)acrylate

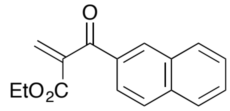

48

Following general procedure C, ethyl 3-(naphthalen-2-yl)-3-oxopropanoate **43** (2.00 g, 8.30 mmol), diisopropylammonium 2,2,2-trifluoroacetate (1.78 g, 8.30 mmol), paraformaldehyde (997 mg, 33.2 mmol) and TFA (64 μL, 0.83 mmol) in THF (60 mL) gave crude product. Column chromatography (10:90 Petrol:EtOAc, R_f = 0.25) to give the title compound (1.01 g, 50%) as a yellow oil: ^{1}H NMR (500 MHz, $CDCl_3$) δ_H: 1.19 (3H, t, *J* 7.1, CH_3), 4.24 (2H, q, *J* 7.1, CH_2CH_3), 6.13 (1H, d, *J* 0.6, =C*H*H), 6.78 (1H, d, *J* 0.6, =CH*H*), 7.56 (1H, ddd, *J* 8.1, 6.9, 1.2, Ar*H*), 7.63 (1H, ddd, *J* 8.2, 6.2, 1.3, Ar*H*), 7.89-8.00 (4H, m, Ar*H*), 8.33 (1H, s, Ar*H*). All data in accordance with literature.[10]

<u>Isothiourea-catalysed Michael addition Lactonisation:</u>

General Procedure D: Isothiourea-catalysed Michael addition-lactonisation

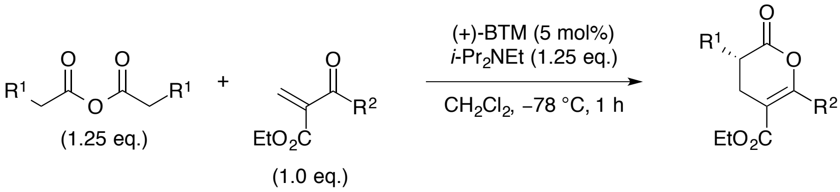

To a solution of requisite homoanhydride (1.25 equiv) in CH_2Cl_2 (0.31 M in homoanhydride) at −78 °C was added Lewis base catalyst (5 mol%) and reaction stirred for 20 min. A solution of Michael acceptor (1.0 equiv) in CH_2Cl_2 (0.25 M), pre-cooled to −78 °C, is added followed by a solution of *i*-Pr_2NEt (1.25 equiv) in CH_2Cl_2 (0.31 M), also pre-cooled to −78 °C, and reaction stirred until complete by TLC analysis. The reaction was quenched with HCl (1 M in H_2O), extracted with CH_2Cl_2 (×3), dried over $MgSO_4$ and concentrated under reduced pressure to give crude residue. Products were purified by Biotage® Isolera™ 4 in the solvent system reported.

Ethyl (*R*)-3-(4-methoxyphenyl)-2-oxo-6-phenyl-3,4-dihydro-2*H*-pyran-5-carboxylate

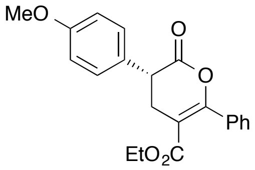

10

Following general procedure D, homoanhydride **36** (198 mg, 0.61 mmol) in CH_2Cl_2 (2 mL), (+)-BTM (5 mg, 0.024 mmol), ethyl 2-benzoylacrylate **1** (100 mg, 0.49 mmol) in CH_2Cl_2 (2 mL) and *i*-Pr_2NEt (106 µL, 0.61 mmol) in CH_2Cl_2 (2 mL) at −78 °C gave crude product. Purification by Biotage® Isolera^{TM} 4 [SNAP 25 g, 75 mL^{-1}, hexane:EtOAc (100:0 1 CV, 100:0 to 70:30 10 CV, 70:30 3 CV)] (hexane:EtOAc 85:15, R_f = 0.30) gave the title compound (143 mg, 83%) as a white solid. mp 113-115 °C; $[\alpha]_D^{20}$ −32.0 (*c* 0.1 CH_2Cl_2); Chiral HPLC analysis, Chiralpak AD-H (80:20 hexane:IPA, flow rate 1 mLmin^{-1}, 254 nm, 30 °C) t_S (3*S*): 33.7 min, t_R (3*R*): 37.5 min; 91% ee; ν_{max} (ATR) 3001, 2837 (C-H), 1775 (C=O dihydropyranone), 1717 (C=O Ester); ^{1}H NMR (400 MHz, $CDCl_3$) 1.00 (3H, t, *J* 7.1, $CO_2CH_2CH_3$), 3.11-3.18 (2H, m, C(4)*H*H and C(4)H*H*), 3.82 (3H, s, ArOC*H*$_3$), 3.94 (1H, dd, *J* 10.7, 7.0, C(3)*H*), 4.04 (2H, q, *J* 7.1, $CO_2CH_2CH_3$), 6.93 (2H, d, *J* 8.8, C(3)Ar(3,5)*H*), 7.25 (2H, d, *J* 8.7, C(3)Ar(2,6)*H*), 7.36-7.45 (5H, m, C(6)Ar(3,5)*H* and C(6)Ar(2,6)*H* and C(6)Ar(4)*H*); ^{13}C{^{1}H} NMR (100 MHz, $CDCl_3$) 13.8 ($CO_2CH_2CH_3$), 29.3 (*C*(4)HH), 43.6 (*C*(3)H), 55.5 (ArO*C*H$_3$), 61.1 ($CO_2CH_2CH_3$), 108.8 (*C*(5)), 114.5 (C(3)Ar*C*(3,5)H), 127.8 (C(6)Ar*C*(4)H), 128.0 (C(6)Ar*C*(2,6)H), 128.8 (C(6)Ar*C*(3,5)H), 129.2 (C(3)Ar*C*(2,6)H), 130.1 (C(6)Ar*C*(1)), 133.1 (C(3)Ar*C*(1)), 158.6 (*C*(6)), 159.4 (C(3)Ar*C*(4)OMe), 166.5 (*C*O$_2$Et), 168.5 (*C*(2)); HRMS (NSI$^+$) $C_{21}H_{20}O_5Na$ [M+Na]$^+$, found 375.1208, requires 375.1203 (+1.3 ppm).

Ethyl (*R*)-3-(4-bromophenyl)-2-oxo-6-phenyl-3,4-dihydro-2*H*-pyran-5-carboxylate

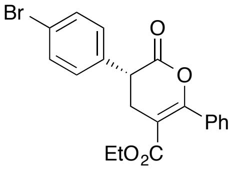

6

Following general procedure D, homoanhydride **5** (80 mg, 0.30 mmol) in CH_2Cl_2 (2 mL), (+)-BTM (3 mg, 0.012 mmol) ethyl 2-benzoylacrylate **1** (50 mg, 0.24 mmol) in CH_2Cl_2 (2 mL) and *i*-Pr_2NEt (52 µL, 0.30 mmol) in CH_2Cl_2 (2 mL) at −78 °C gave crude product. Purification by Biotage® Isolera^{TM} 4 [SNAP 25 g, 75 mL^{-1}, hexane:EtOAc (100:0 1 CV, 100:0 to 70:30 10 CV, 70:30 3 CV)] (hexane:EtOAc 85:15, R_f = 0.38) gave the title

compound (44 mg, 69%) as a white solid. mp 176-177 °C; $[\alpha]_D^{20}$ −42.0 (*c* 0.1 CH_2Cl_2); Chiral HPLC analysis, Chiralpak AD-H (80:20 hexane:IPA, flow rate 1 $mLmin^{-1}$, 254 nm, 30 °C) t_R(*R*): 13.6 min, t_R(*S*): 15.5 min, 90% ee; ν_{max} (ATR) 2984, 2905 (C-H), 1769 (C=O dihydropyranone), 1694 (C=O Ester); ^1H NMR (400 MHz, $CDCl_3$) 0.99 (3H, t, *J* 7.1, $CO_2CH_2CH_3$), 3.06 (1H, dd, *J* 17.2, 11.4, C(4)*H*H), 3.16 (1H, dd, *J* 17.2, 6.8, C(4)H*H*), 3.94 (1H, dd, *J* 11.3, 6.8, C(3)*H*), 4.04 (2H, q, *J* 7.1, $CO_2CH_2CH_3$), 7.20 (2H, d, *J* C(3)Ar(2,6)*H*), 7.37-7.45 (5H, m, C(6)Ar(3,5)*H* and C(6)Ar(2,6)*H* and C(6)Ar(4)*H*), 7.53 (2H, d, *J* 8.4, C(3)Ar(3,5)*H*); $^{13}C\{^1H\}$ NMR (100 MHz, $CDCl_3$) 13.8 ($CO_2CH_2CH_3$), 29.1 (*C*(4)HH), 43.9 (ArO*C*H_3), 61.2 ($CO_2CH_2CH_3$), 108.7 (*C*(5)), 122.3 (C(3)Ar*C*(4)), 128.1 (C(6)Ar*C*(2,6)H), 128.8 (C(6)Ar*C*(3,5)H), 129.9 (C(3)Ar*C* (3,5)H), 130.3 (C(6)Ar*C*(4)H), 132.2 (C(3)Ar*C*(2,6)H), 132.9 (C(6)Ar*C*(1)), 134.8 (C(3)Ar*C*(1)), 158.7 (*C*(6)), 166.3 (*C*O_2Et), 167.7 (*C*(2)); HRMS (NSI^+) $C_{20}H_{17}BrO_4Na$ $[M+Na]^+$, found 423.0198, requires 423.0202 (−1.0 ppm).

Ethyl (*R*)-3-(4-chlorophenyl)-2-oxo-6-phenyl-3,4-dihydro-2*H*-pyran-5-carboxylate

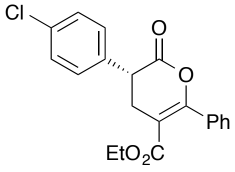

11

Following general procedure D, homoanhydride **37** (197 mg, 0.61 mmol) in CH_2Cl_2 (2 mL), (+)-BTM (5 mg, 0.024 mmol), ethyl 2-benzoylacrylate **1** (100 mg, 0.49 mmol) in CH_2Cl_2 (2 mL) and *i*-Pr_2NEt (106 µL) in CH_2Cl_2 (2 mL) at −78 °C gave crude product. Purification by Biotage® Isolera™ 4 [SNAP 25 g, 75 mL^{-1}, hexane:EtOAc (100:0 1 CV, 100:0 to 70:30 10 CV, 70:30 3 CV)] (hexane:EtOAc 85:15, R_f = 0.38) gave the title compound (148 mg, 85%) as a white solid. mp 168-170 °C; $[\alpha]_D^{20}$ −21.0 (*c* 0.1 CH_2Cl_2); Chiral HPLC analysis, Chiralpak AD-H (95:5 hexane:IPA, flow rate 1 $mLmin^{-1}$, 220 nm, 30 °C) t_R(*R*): 29.9 min, t_R(*S*): 34.3 min, 88% ee; ν_{max} (ATR) 2980 (C-H), 1752 (C=O dihydropyranone), 1697 (C=O Ester); ^1H NMR (500 MHz, $CDCl_3$) 1.00 (3H, t, *J* 7.1, $CO_2CH_2CH_3$), 3.07 (1H, dd, *J* 17.2, 11.4, C(4)*H*H), 3.16 (1H, dd, *J* 17.2, 6.8, C(4)H*H*), 3.96 (1H, dd, *J* 11.3, 6.8, C(3)*H*), 4.04 (2H, q, *J* 7.1, $CO_2CH_2CH_3$), 7.27 (2H, d, *J* 6.9, C(3)Ar(3,5)*H*), 7.37-7.45 (7H, m, C(6)Ar(3,5)*H* and C(3)Ar(2,6)*H* and C(6)Ar(4)*H* and C(6)Ar(2,6)*H*); $^{13}C\{^1H\}$ NMR (125 MHz, $CDCl_3$) 13.8 ($CO_2CH_2CH_3$), 29.2 (*C*(4)HH), 43.8 (*C*(3)H), 61.2 ($CO_2CH_2CH_3$), 108.7 (*C*(5)), 128.1 (C(6)Ar*C*(2,6)H), 128.8 (C(6)Ar*C*(3,5)H), 129.3 (C(3)Ar*C*(3,5)H), 129.6 (C(6)Ar*C*(4)H), 130.3 (C(3)Ar*C*(2,6)), 132.9 (C(3)Ar*C*(4)), 134.2 (C(3)Ar*C*(1)), 134.2

(C(6)Ar*C*(1)), 158.7 (*C*(6)), 166.3 (*C*O_2Et), 167.9 (*C*(2)); HRMS (APCI$^+$) $C_{20}H_{18}Cl^{35}O_4$ $[M+H]^+$, found 357.0888, requires 357.0888 (0.0 ppm).

Ethyl (*R*)-3-(naphthalen-1-yl)-2-oxo-6-phenyl-3,4-dihydro-2*H*-pyran-5-carboxylate

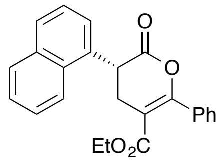

12

Following general procedure D, homoanhydride **38** (216 mg, 0.61 mmol) in CH_2Cl_2 (2 mL), (+)-BTM (5 mg, 0.024 mmol), ethyl 2-benzoylacrylate **1** (100 mg, 0.49 mmol) in CH_2Cl_2 (2 mL) and *i*-Pr_2NEt (106 μL, 0.61 mmol) in CH_2Cl_2 (2 mL) at −78 °C gave crude product. Purification by Biotage® Isolera™ 4 [SNAP 25 g, 75 mL^{-1}, hexane:EtOAc (100:0 1 CV, 100:0 to 80:20 10 CV, 80:20 3 CV)] (hexane:EtOAc 85:15, R_f = 0.55) gave the title compound (147 mg, 81%) as a white solid. mp 124-126 °C; $[\alpha]_D^{20}$ +6.0 (*c* 0.1 CH_2Cl_2); Chiral HPLC analysis, Chiralpak OD-H (80:20 hexane:IPA, flow rate 1 mLmin^{-1}, 211 nm, 30 °C) t_R(*S*): 18.2 min, t_R(*R*): 26.3 min, 97% ee; ν_{max} (ATR) 3052, 2990 (C-H), 1759 (C=O dihydropyranone), 1705 (C=O Ester); ^{1}H NMR (500 MHz, $CDCl_3$) 0.99 (3H, t, *J* 7.1, $CO_2CH_2CH_3$), 3.25-3.34 (2H, m, C(4)*H*H and C(4)H*H*), 4.03 (2H, q, *J* 7.1, $CO_2CH_2CH_3$), 4.73 (1H, dd, *J* 10.3, 8.1, C(3)*H*), 7.41-7.59 (9H, m, Ar*H*), 7.86-7.88 (1H, m, Ar*H*), 7.92 (1H, d, *J* 8.0, Ar*H*), 7.96 (1H, d, *J* 8.4, Ar*H*); $^{13}C\{^1H\}$ NMR (125 MHz, $CDCl_3$) 13.8 ($CO_2CH_2CH_3$), 29.2 (*C*(4)HH), 41.1 (*C*(3)*H*), 61.2 ($CO_2CH_2CH_3$), 109.0 (*C*(5)), 123.0 (Ar*C*H), 125.5 (Ar*C*H), 125.7 (Ar*C*H), 126.1 (Ar*C*H), 126.8 (Ar*C*H), 128.1 (Ar*C*H), 128.9 (Ar*C*H), 129.1 (Ar*C*H), 129.4 (Ar*C*H), 130.2 (Ar*C*H), 131.2 (Ar*C*), 132.1 (Ar*C*), 133.1 (Ar*C*), 134.2 (Ar*C*), 158.6 (*C*(6)), 166.4 (*C*O_2Et), 168.0 (*C*(2)); HRMS (APCI$^+$) $C_{24}H_{21}O_4$ $[M+H]^+$, found 373.1437, requires 373.1434 (+0.7 ppm).

Ethyl (*R*)-2-oxo-6-phenyl-3-(*o*-tolyl)-3,4-dihydro-2*H*-pyran-5-carboxylate

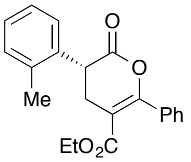

13

Following general procedure D, homoanhydride **39** (178 mg, 0.61 mmol) in CH_2Cl_2 (2 mL), (+)-BTM (5 mg, 0.024 mmol), ethyl 2-benzoylacrylate **1** (100 mg, 0.49 mmol) in CH_2Cl_2 (2 mL) and *i*-Pr_2NEt (106 μL, 0.61 mmol) in CH_2Cl_2 (2 mL) at −78 °C gave crude product. Purification by Biotage® Isolera™ 4 [SNAP 25 g, 75 mL^{-1}, hexane:EtOAc (100:0 1 CV,

100:0 to 80:20 10 CV, 80:20 3 CV)] (hexane:EtOAc 85:15, R_f = 0.55) gave the title compound (134 mg, 81%) as a white solid. mp 100-102 °C; $[\alpha]_D^{20}$ −10.0 (*c* 0.1 CH_2Cl_2); Chiral HPLC analysis, Chiralpak OJ-H (70:30 hexane:IPA, flow rate 1 $mLmin^{-1}$, 220 nm, 30 °C) t_R(*S*): 15.1 min, t_R(*R*): 24.4 min, 50% ee; ν_{max} (ATR) 2978 (C-H), 1767 (C=O dihydropyranone), 1694 (C=O Ester); ^{1}H NMR (500 MHz, $CDCl_3$) 1.01 (3H, t, *J* 7.1, $CO_2CH_2CH_3$), 3.06 (1H, dd, *J* 16.9, 12.6, C(4)*H*H), 3.13 (1H, dd, *J* 17.2, 7.3, C(4)H*H*), 4.04 (2H, q, *J* 7.1, $CO_2CH_2CH_3$), 4.14 (1H, dd, *J* 12.5, 7.3, C(3)*H*), 7.25-7.26 (4H, m, C(3)Ar(3)*H* and C(3)Ar(4)*H* and C(3)Ar(5)*H* and C(3)Ar(6)*H*), 7.39-7.50 (5H, m, C(6)Ar(3,5)*H* and C(6)Ar(2,6)*H* and C(6)Ar(4)*H*); $^{13}C\{^1H\}$ NMR (125 MHz, $CDCl_3$) 13.8 ($CO_2CH_2CH_3$), 19.9 (Ar*C*H_3), 28.9 (*C*(4)HH), 41.3 (*C*(3)*H*), 61.2 ($CO_2CH_2CH_3$), 109.1 (*C*(5)), 126.8 (C(3)Ar*C*(5)H), 127.5 (C(3)Ar*C*(6)H), 128.1 (C(6)Ar*C*(3,5)H), 128.1 (C(3)Ar*C*(4)H), 128.9 (C(6)Ar*C*(2,6)H), 130.2 (C(6)Ar*C*(4)H), 131.1 (C(3)Ar*C*(3)H), 133.1 (C(3)Ar*C*(1)), 134.6 (C(6)Ar*C*(1)), 136.5 (C(3)Ar*C*(2)), 158.7 (*C*(6)), 166.4 (*C*O_2Et), 168.0 (*C*(2)); HRMS ($APCI^+$) $C_{21}H_{21}O_4$ $[M+H]^+$, found 337.1435, requires 337.1434 (+0.2 ppm).

Ethyl (*R*)-6-(4-methoxyphenyl)-2-oxo-3-phenyl-3,4-dihydro-2*H*-pyran-5-carboxylate

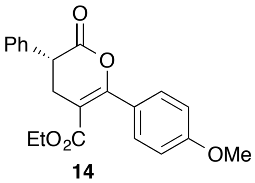

14

Following general procedure D, homoanhydride **2** (140 mg, 0.53 mmol) in CH_2Cl_2 (2 mL), (+)-BTM (5 mg, 0.024 mmol) ethyl 2-(4-methoxybenzoyl)acrylate **44** (100 mg, 0.43 mmol) in CH_2Cl_2 (2 mL) and *i*-Pr_2NEt (93 μL, 0.53 mmol) in CH_2Cl_2 (2 mL) at −78 °C gave crude product. Purification by Biotage® Isolera™ 4 [SNAP 25 g, 75 mL^{-1}, hexane:EtOAc (100:0 1 CV, 100:0 to 70:30 10 CV, 70:30 3 CV)] (hexane:EtOAc 85:15, R_f = 0.25) gave the title compound (116 mg, 77%) as a white solid. mp 96-98 °C; $[\alpha]_D^{20}$ −63.0 (*c* 0.1 CH_2Cl_2); Chiral HPLC analysis, Chiralpak AD-H (80:20 hexane:IPA, flow rate 1 $mLmin^{-1}$, 254 nm, 30 °C) t_R(*S*): 12.1 min, t_R(*R*): 16.0 min, 91% ee; ν_{max} (ATR) 2978, 2968 (C-H), 1773 (C=O dihydropyranone), 1713 (C=O Ester); ^{1}H NMR (500 MHz, $CDCl_3$) 1.08 (3H, t, *J* 7.1, $CO_2CH_2CH_3$), 3.06-3.19 (2H, m, C(4)*H*H and C(4)H*H*), 3.84 (3H, s, ArOC*H*$_3$), 3.96 (1H, dd, *J* 11.1, 6.9, C(3)*H*), 4.08 (2H, q, *J* 7.1, $CO_2CH_2CH_3$), 6.90 (2H, d, *J* 8.8, C(6)Ar(3,5)*H*), 7.31-7.35 (3H, m, C(3)Ar(3,5)*H* and C(3)Ar(4)*H*), 7.39-7.42 (4H, m, C(3)Ar(2,6)*H* and C(6)Ar(2,6)*H*); $^{13}C\{^1H\}$ NMR (125 MHz, $CDCl_3$) 14.0 ($CO_2CH_2CH_3$), 29.5 (*C*(4)HH), 44.5 (ArO*C*H_3), 55.5 (*C*(3)H), 61.1 ($CO_2CH_2CH_3$), 107.8 (*C*(5)), 113.4 (C(6)Ar*C*(3,5)H), 125.2 (C(6)Ar*C*(1)), 128.1 (C(3)Ar*C*(4)H), 128.2 (C(3)Ar*C*(3,5)H), 129.1 (C(6)Ar*C*(2,6)H), 130.6 (C(3)Ar*C*(2,6)H), 136.0 (C(3)Ar*C*(1)), 158.5 (*C*(6)), 161.2 (C(6)Ar*C*(4)), 166.6 (*C*O_2Et),

168.5 (C(2)); HRMS (NSI$^+$) $C_{21}H_{20}O_5Na$ [M+Na]$^+$, found 375.1208, requires 375.1203 (+1.3 ppm).

Ethyl (*R*)-2-oxo-3-phenyl-6-(*p*-tolyl)-3,4-dihydro-2*H*-pyran-5-carboxylate

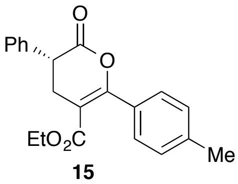

15

Following general procedure D, homoanhydride **2** (153 mg, 0.58 mmol) in CH_2Cl_2 (2 mL), (+)-BTM (5 mg, 0.023 mmol) ethyl 2-(4-methylbenzoyl)acrylate **45** (100 mg, 0.46 mmol) in CH_2Cl_2 (2 mL) and Na_2CO_3 (61 mg, 0.58 mmol) in CH_2Cl_2 (2 mL) at −78 °C gave crude product. Purification by Biotage® Isolera™ 4 [SNAP 25 g, 75 mL^{-1}, hexane:EtOAc (100:0 1 CV, 100:0 to 70:30 10 CV, 70:30 3 CV)] (hexane:EtOAc 85:15, R_f = 0.40) gave the title compound (101 mg, 69%) as a white solid. mp 72-74 °C; $[\alpha]_D^{20}$ −36.0 (c 0.1 CH_2Cl_2); Chiral HPLC analysis, Chiralpak AD-H (90:10 hexane:IPA, flow rate 1 mLmin^{-1}, 220 nm, 30 °C) t_R(*S*): 15.3 min, t_R(*R*): 18.8 min, 84% ee; ν_{max} (ATR) 2998, 2986 (C-H), 1755 (C=O dihydropyranone), 1717 (C=O Ester); ^{1}H NMR (400 MHz, $CDCl_3$) 1.05 (3H, t, J 7.1, $CO_2CH_2CH_3$), 2.39 (3H, s, ArCH_3), 3.06-3.19 (2H, m, C(4)*H*H and C(4)H*H*), 3.97 (1H, dd, J 10.8, 7.1, C(3)*H*), 4.06 (2H, q, J 7.1, $CO_2CH_2CH_3$), 7.18-7.20 (2H, m, C(6)Ar(3,5)*H*), 7.30-7.36 (5H, m, C(3)Ar(3,5)*H* and C(3)Ar(4)*H* and C(6)Ar(2,6)*H*), 7.38-7.42 (2H, m, C(6)Ar(2,6)*H*); $^{13}C\{^1H\}$ NMR (100 MHz, $CDCl_3$) 13.9 ($CO_2CH_2CH_3$), 21.6 (ArCH_3) 29.4 (C(4)HH), 44.4 (ArOCH_3), 61.1 ($CO_2CH_2CH_3$), 108.3 (C(5)), 128.1 (C(3)ArC(4)H), 128.2 (C(6)ArC(3,5)H), 128.7 (C(3)ArC(3,5)H), 128.8 (C(6)ArC(2,6)H), 129.1 (C(3)ArC(2,6)H), 135.9 (C(6)ArC(1)), 140.4 (C(3)ArC(1)), 158.8 (C(6)), 166.5 (CO_2Et), 168.3 (C(2)); HRMS (NSI$^+$) $C_{21}H_{21}O_4$ [M+H]$^+$, found 337.1437, requires 337.1434 (+0.8 ppm).

Ethyl (*R*)-6-(furan-2-yl)-3-(4-methoxyphenyl)-2-oxo-3,4-dihydro-2*H*-pyran-5-carboxylate

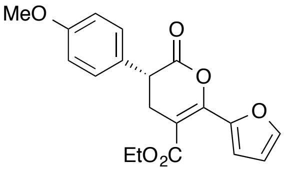

16

Following general procedure D, homoanhydride **36** (208 mg, 0.64 mmol) in CH_2Cl_2 (2 mL), (+)-BTM (5 mg, 0.024 mmol) ethyl 2-(furan-2-carbonyl)acrylate (100 mg, 0.51 mmol) in CH_2Cl_2 (2 mL) and i-Pr_2NEt (111 µL, 0.64 mmol) in CH_2Cl_2 (2 mL) at −78 °C gave crude product. Purification by Biotage® Isolera™ 4 [SNAP 25 g, 75 mL^{-1}, hexane:EtOAc (100:0 1

CV, 100:0 to 70:30 10 CV, 70:30 3 CV)] (hexane:EtOAc 85:15, R_f = 0.25) gave the title compound (107 mg, 61%) as a white solid. mp 92-94 °C; $[\alpha]_D^{20}$ −34.0 (*c* 0.1 CH_2Cl_2); Chiral HPLC analysis, Chiralpak OD-H (90:10 hexane:IPA, flow rate 1 mLmin^{-1}, 254 nm, 30 °C) t_R(*R*): 17.6 min, t_S(*S*): 21.4 min, 99% ee; ν_{max} (ATR) 2980, 2941 (C-H), 1778 (C=O dihydropyranone), 1713 (C=O Ester); ^{1}H NMR (400 MHz, $CDCl_3$) 1.25 (3H, t, *J* 7.1, $CO_2CH_2CH_3$), 3.01-3.13 (2H, m, C(4)*H*H and C(4)H*H*), 3.81 (3H, s, ArOC*H*$_3$), 3.90 (1H, dd, *J* 10.5, 7.6, C(3)*H*), 4.24 (2H, q, *J* 7.1, $CO_2CH_2CH_3$), 6.49 (1H, dd, *J* 3.5, 1.8, C(6)Ar(4)*H*), 6.89-6.93 (3H, m, C(6)Ar(3)*H* and C(3)Ar(3,5)*H*), 7.22 (2H, d, *J* 8.6, C(3)Ar(2,6)*H*), 7.48 (1H, dd, C(6)Ar(5)*H*); ^{13}C{^{1}H} NMR (100 MHz, $CDCl_3$) 14.3 ($CO_2CH_2CH_3$), 29.8 (*C*(4)HH), 43.7 (*C*(3)H), 55.5 (ArO*C*H$_3$), 61.5 ($CO_2CH_2CH_3$), 108.0 (*C*(5)), 111.7 (C(6)Ar*C*(4)H), 113.6 (C(6)Ar*C*(3)H), 114.5 (C(3)Ar*C*(3,5)H), 127.7 (C(3)Ar*C*(1)), 129.3 (C(3)Ar*C*(2,6)H), 144.2 (C(6)Ar*C*(5)H) 145.5 (C(3)Ar*C*(4)), 146.2 (*C*(6)), 149.6 (C(6)Ar*C*(2)) 159.4 (C(3)Ar*C*(4)), 166.4 (*C*O$_2$Et), 168.1 (*C*(2)); HRMS (NSI$^+$) $C_{19}H_{18}O_6Na$ [M+Na]$^+$, found 365.1003, requires 365.0996 (+2.0 ppm).

Ethyl (*R*)-6-(3-bromophenyl)-3-(4-methoxyphenyl)-2-oxo-3,4-dihydro-2*H*-pyran-5-carboxylate

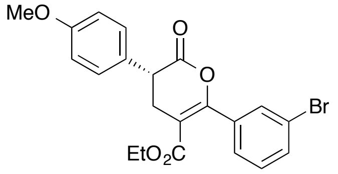

17

Following general procedure D, homoanhydride **36** (243 mg, 0.44 mmol) in CH_2Cl_2 (2 mL), (+)-BTM (5 mg, 0.024 mmol), ethyl 2-(3-bromobenzoyl)acrylate **47** (100 mg 0.35 mmol) in CH_2Cl_2 (2 mL) and *i*-Pr$_2$NEt (77 μL, 0.44 mmol) in CH_2Cl_2 (2 mL) at −78 °C gave crude product. Purification by Biotage® Isolera^{TM} 4 [SNAP 25 g, 75 mL^{-1}, hexane:EtOAc (100:0 1 CV, 100:0 to 70:30 10 CV, 70:30 3 CV)] (hexane:EtOAc 85:15, R_f = 0.25) gave the title compound (92 mg, 61%) as a white solid. mp 96-98 °C; $[\alpha]_D^{20}$ −33.0 (*c* 0.1 CH_2Cl_2); Chiral HPLC analysis, Chiralpak OD-H (90:10 hexane:IPA, flow rate 1 mLmin^{-1}, 211 nm, 30 °C) t_R(*R*): 18.4 min, t_R(*S*): 26.2 min, 68% ee; ν_{max} (ATR) 2982, 2902 (C-H), 1773 (C=O dihydropyranone), 1697 (C=O Ester); ^{1}H NMR (400 MHz, $CDCl_3$) 1.04 (3H, t, *J* 7.1, $CO_2CH_2CH_3$), 3.04-3.18 (2H, m, C(4)*H*H and C(4)H*H*), 3.82 (3H, s, ArOC*H*$_3$), 3.93 (1H, dd, *J* 10.8, 7.0, C(3)*H*), 4.06 (2H, q, *J* 7.1, $CO_2CH_2CH_3$), 6.94 (2H, d, *J* 8.8, C(3)Ar(3,5)*H*), 7.21-7.28 (3H, m, C(6)Ar(5)*H*) and C(3)Ar(2,6)*H*), 7.36 (1H, dt, 7.8, 1.3, C(6)Ar(4)*H*), 7.54-7.58 (2H, m, C(6)Ar(2)*H* and C(6)Ar(6)*H*); ^{13}C{^{1}H} NMR (100 MHz, $CDCl_3$) 13.8 ($CO_2CH_2CH_3$), 29.2 (*C*(4)HH), 43.5 (*C*(3)H), 55.5 (ArO*C*H$_3$), 61.3 ($CO_2CH_2CH_3$), 109.7 (*C*(5)), 114.6

(C(3)Ar*C*(3,5)H), 121.9 (C(6)Ar*C*(3)), 127.5 (C(6)Ar*C*(4)H), 129.2 (C(3)Ar*C*(2,6)H), 129.2 (C(3)Ar*C*(1)), 129.6 (C(6)Ar*C*(5)H), 131.9 (C(6)Ar*C*(5)H), 133.1 (C(6)Ar*C*(2)H), 135.0 (C(6)Ar*C*(1)), 156.8 (*C*(6)), 159.5 (C(3)Ar*C*(4)), 166.0 (*C*O$_2$Et), 168.2 (*C*(2)); HRMS (NSI$^+$) $C_{21}H_{19}Br^{79}O_5Na$ [M+Na]$^+$, found 453.0301, requires 453.0308 (−1.6 ppm).

Ethyl (*R*)-3-(4-methoxyphenyl)-6-(naphthalen-2-yl)-2-oxo-3,4-dihydro-2*H*-pyran-5-carboxylate

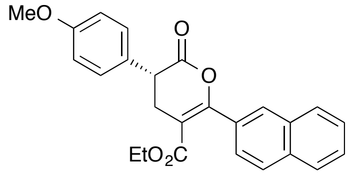

18

Following general procedure D, homoanhydride **36** (140 mg, 0.53 mmol) in CH_2Cl_2 (2 mL), (+)-BTM (5 mg, 0.024 mmol), ethyl 2-(2-naphthoyl)acrylate **48** (100 mg, 0.39 mmol) in CH_2Cl_2 (2 mL) and *i*-Pr$_2$NEt (93 μL, 0.53 mmol) in CH_2Cl_2 (2 mL) at −78 °C gave crude product. Purification by Biotage® Isolera^{TM} 4 [SNAP 25 g, 75 mL^{-1}, hexane:EtOAc (100:0 1 CV, 100:0 to 70:30 10 CV, 70:30 3 CV)] (hexane:EtOAc 85:15, R_f = 0.26) gave the title compound (116 mg, 77%) as a white solid. mp 88-90 °C; $[\alpha]_D^{20}$ −8.0 (*c* 0.1 CH_2Cl_2); Chiral HPLC analysis, Chiralpak OD-H (90:10 hexane:IPA, flow rate 1 mLmin^{-1}, 220 nm, 30 °C) t_R(*S*): 24.1 min, t_R(*R*): 28.2 min, 86% ee; ν_{max} (ATR) 2980, 2902 (C-H), 1771 (C=O dihydropyranone), 1695 (C=O Ester); ^{1}H NMR (500 MHz, $CDCl_3$) 0.94 (3H, t, 7.1, $CO_2CH_2CH_3$), 3.10-3.23 (2H, m, C(4)*H*H and C(4)H*H*), 3.82 (3H, s, ArOC*H$_3$*), 3.97-4.06 (3H, m, C(3)*H* and CO$_2$C*H$_2$*CH$_3$), 6.95 (2H, d, *J* 8.7, C(3)Ar(3,5)*H*), 7.28 (2H, d, *J* 8.7, C(3)Ar(2,6)*H*), 7.49-7.54 (3H, m, Ar*H*), 7.83-7.86 (3H, m, Ar*H*), 7.99 (1H, s, Ar*H*); ^{13}C{^{1}H} NMR (125 MHz, $CDCl_3$) 13.8 ($CO_2CH_2CH_3$), 29.5 (*C*(4)HH), 43.7 (*C*(3)H), 55.5 (ArO*C*H$_3$), 61.2 (CO$_2$*C*H$_2$CH$_3$), 109.1 (*C*(5)), 114.5 (C(3)Ar*C*(3,5)H), 126.0 (C(6)Ar*C*H), 126.6 (C(6)Ar*C*H), 127.4 (C(6)Ar*C*H), 127.5 (C(6)Ar*C*H), 127.8 (C(3)Ar*C*(1)), 127.8 (C(6)Ar*C*H), 128.7 (C(6)Ar*C*H), 128.9 (C(6)Ar*C*H), 129.2 (C(3)Ar*C*(2,6)H), 130.3 (C(6)Ar*C*), 132.6 (C(6)Ar*C*), 134.0 (C(6)Ar*C*(2)), 158.3 (*C*(6)), 159.4 (C(3)Ar*C*(4)), 166.6 (*C*O$_2$Et), 168.6 (*C*(2)); HRMS (NSI$^+$) $C_{25}H_{22}O_5Na$ [M+Na]$^+$, found 425.1355, requires 425.1359 (−1.0 ppm).

Derivatisation of Dihydropyranone 10

Ethyl (2*S*,3*R*,5*R*)-5-(4-methoxyphenyl)-6-oxo-2-phenyltetrahydro-2*H*-pyran-3-carboxylate

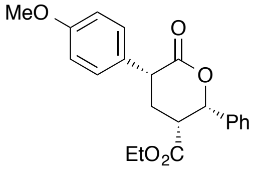

19

To a solution of **10** (150 mg, 0.43 mmol) in EtOAc (11 mL) was added 10% Pd/C (46 mg, 0.043 mmol), a balloon of hydrogen was appended and the reaction stirred at rt for 24 h. Reaction mixture was filtered through celite and concentrated under reduced pressure to give crude reaction product. Purification by Biotage® Isolera™ 4 [SNAP 25 g, 75 mL^{-1}, hexane:EtOAc (100:0 1 CV, 100:0 to 70:30 10 CV, 70:30 3 CV)] (hexane:EtOAc 85:15, R_f = 0.25) gave the title compound gave title compound (91 mg, 60% yield) as white solid. mp 128-130 °C; $[\alpha]_D^{20}$ −51.0 (*c* 0.1 CH_2Cl_2); ν_{max} (ATR) 2984, 2979 (C-H), 1769 (C=O dihydropyranone), 1690 (C=O Ester); ^{1}H NMR (300 MHz, $CDCl_3$) 0.98 (3H, t, *J* 7.15, $CO_2CH_2CH_3$), 2.37-2.60 (2H, m, C(4)*H*H and C(4)H*H*), 3.39 (1H, dd, *J* 8.2, 6.0, 4.5, C(3)*H*), 3.82 (3H, s, ArOC*H₃*) 3.84-4.01 (3H, m, C(5)*H* and $CO_2CH_2CH_3$), 5.81 (1H, d, *J* 4.4, C(2)*H*), 6.93 (2H, d, *J* 8.8, C(5)Ar(3,5)*H*), 7.26-7.30 (2H, m, C(5)Ar(2,6)*H*), 7.33-7.40 (5H, m, C(2)Ar(3,5)*H* and C(2)Ar(4)*H* and C(2)Ar(2,6)*H*); $^{13}C\{^1H\}$ NMR (75 MHz, $CDCl_3$) 13.90 (CO_2CHCH_3), 29.6 (*C*(4)HH), 44.6 (*C*(5)H), 45.7 (*C*(3)*H*), 55.5 (ArO*C*H$_3$), 61.2 ($CO_2CH_2CH_3$), 78.7 (*C*(2)H), 114.4 (C(5)Ar*C*(3,5)H), 125.9 (C(2)Ar*C*(3,5)H), 128.6 (C(2)Ar*C*(2,6)H), 128.6 (C(2)Ar*C*(4)H), 129.5 (C(5)Ar*C*(1)), 129.9 (C(5)Ar*C*(2,6)H), 136.4 (C(2)Ar*C*(1)), 159.2 (C(5)Ar*C*(4)), 171. 3 (*C*(6)), 172.4 (*C*O$_2$Et); HRMS (NSI$^+$) $C_{21}H_{22}O_5Na$ $[M+Na]^+$, found 377.1355, requires 377.1359 (−1.2 ppm).

(1*S*,2*S*,4*R*)-2-(hydroxymethyl)-4-(4-methoxyphenyl)-1-phenylpentane-1,5-diol

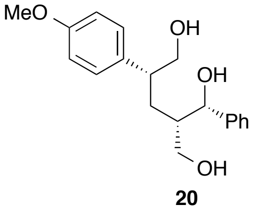

20

To a solution of **19** (42 mg, 0.13 mmol) in anhydrous THF (2 mL), under an N_2 atmosphere, at 0 °C was added dropwise $LiAH_4$ (195 µL, 0.39 mmol, 2 M in THF). The reaction was stirred for 10 min then quenched with 0.1 M HCl (5 ml). The reaction mixture was extracted with EtOAc (×3) and combined organics dried over $MgSO_4$, filtered and concentrated under

reduced pressure to give crude reaction product. Purification by Biotage® Isolera™ 4 [SNAP 10 g, 25 mL^{-1}, hexane:EtOAc (100:0 1 CV, 100:0 to 90:10 10 CV, 90:10 3 CV)] (hexane:EtOAc 85:15, $R_f = 0.25$) gave the title compound gave the title compound (36 mg, 88% yield) as colourless oil. $[\alpha]_D^{20}$ −42.0 (*c* 0.1 CH_2Cl_2); ν_{max} (ATR); 3301 (OH), 2909 (C-H) ^1H NMR (500 MHz, $CDCl_3$) 1.59-1.65 (2H, m, C(3)H_2), 2.32 (1H, tt, *J* 10.4, 4.4, C(2)*H*), 2.77 (1H, br. s. O*H*), 2.82-2.87 (1H, m, C(4)*H*), 3.41-3.43 (2H, m, C(2)C(1')H_2), 3.65-3.68 (3H, m, C(5)H_2 and O*H*), 3.80 (3H, s, ArOCH_3), 4.25 (1H, br. s, O*H*), 4.94-4.96 (1H, C(1)*H*), 6.89 (2H, d, *J* 8.7, C(4)Ar(3,5)*H*), 7.12 (2H, d, *J* 8.7, C(4)Ar(2,6)*H*), 7.20-7.26 (3H, m, C(1)Ar(3,5)*H* and C(1)Ar(4)*H*), 7.30-7.33 (2H, C(1)Ar(2,6)*H*); $^{13}C\{^1H\}$ NMR (125 MHz, $CDCl_3$) 31.0 (*C*(3)H_2), 43.4 (*C*(2)H), 45.1 (*C*(4)H), 55.4 (ArO*C*H_3), 65.1 (C(2)*C*(1')H_2), 68.0 (*C*(5)H_2), 76.1 (*C*(1)H), 114.4 (C(4)Ar*C*(3,5)H), 125.9 (C(1)Ar*C*(4)H), 127.4 (C(1)Ar*C*(3,5)H), 128.5 (C(1)Ar*C*(2,6)H), 129.0 (C(4)Ar*C*(2,6)H), 133.4 (C(4)Ar*C*(1)), 143.4 (C(1)Ar*C*(1)), 158.7 (C(4)Ar*C*(4)); HRMS (CI^+) $C_{19}H_{26}NO_3$ $[M+NH_4]^+$, found 334.2011, requires 334.2013 (−0.6 ppm).

Determination of Relative Stereochemistry for 19

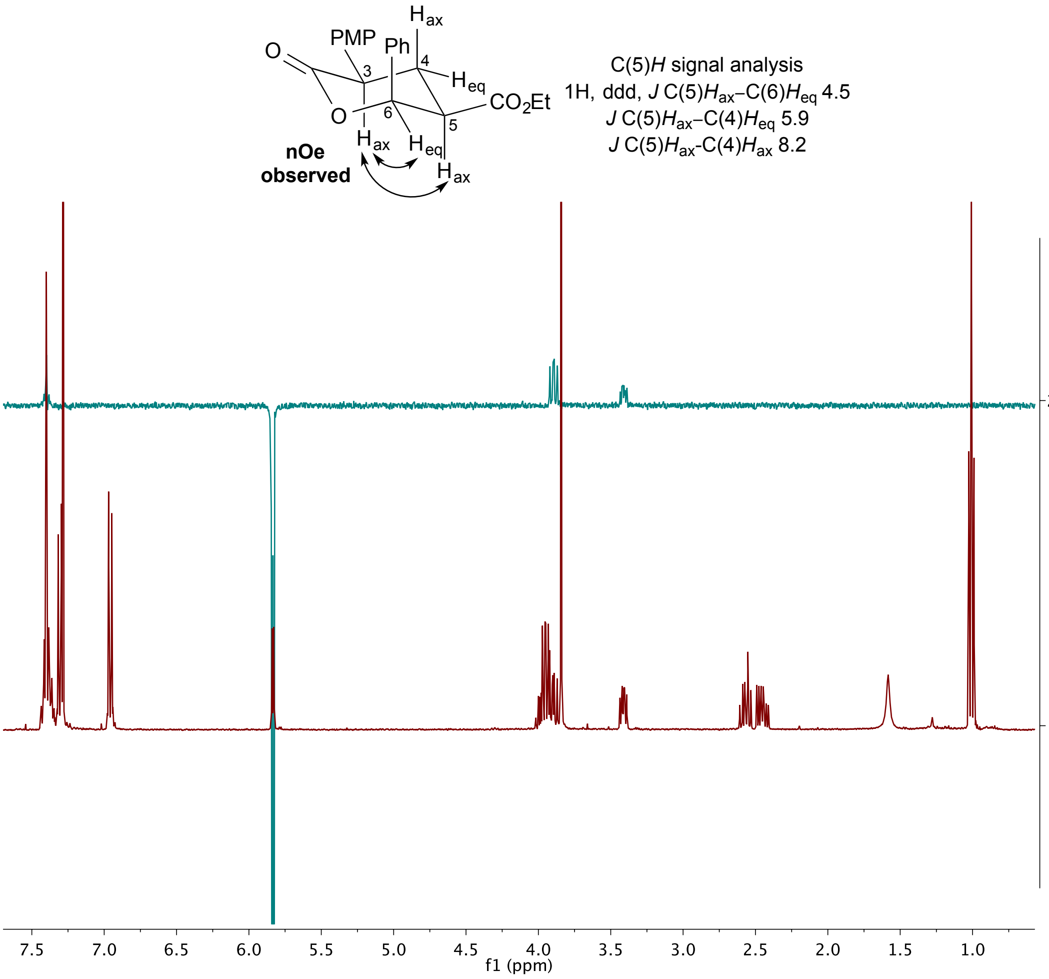

Figure S1. NOESY Experiment for **19**; irradiation of C(2)*H* (5.81 ppm).

3.0 Isothiourea-Catalysed Michael Addition-Lactamisation

Preparation of 2-(aryl(tosylimino)methyl)acrylates

General Procedure E: Preparation of ketimines

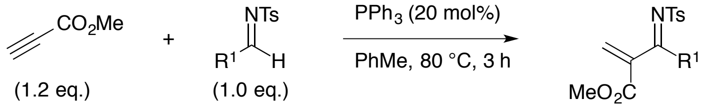

To a solution of requisite aldimine (1.0 equiv) and triphenylphosphine (0.2 equiv) in toluene [0.07 M in aldimine] at 80 °C was added a solution of requisite propiolate (1.2 equiv) in toluene [0.24 M in propiolate] over 3 hours. The reaction was concentrated under reduced pressure to give crude reaction mixture. Products were purified by column chromatography in the solvent system reported. All aldimines used for the preparation of 2-*N*-tosyliminoacrylates were prepared as described in the literature.[11]

Methyl 2-(phenyl(tosylimino)methyl)acrylate

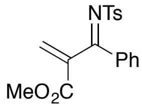

2

Following general procedure E, *N*-benzylidene-4-methylbenzenesulfonamide (3.89 g, 15.0 mmol), triphenylphosphine (0.79 g, 3.00 mmol) in toluene (230 mL) and methyl propiolate (0.37 mL, 4.18 mmol) in toluene (20 mL) for 3 h at 80 °C gave, after column chromatography (eluent: Et_2O:Petrol 80:20, R_f = 0.25) the title compound as a white solid (708 mg, 14%). mp 100-102 °C; ^{1}H NMR (500 MHz, $CDCl_3$) δ_H: 2.45 (3H, s, ArC*H*$_3$), 3.78 (3H, s, CO_2C*H*$_3$), 5.93 (1H, s, =C*H*H), 6.83 (1H, s, =CH*H*), 7.34 (2H, d, *J* 8.1, SO_2ArC(3,5)*H*), 7.41 (2H, t, *J* 7.8, ArC(3,5)*H*), 7.53-7.59 (1H, m, ArC(4)*H*), 7.84-7.92 (4H, m, ArC(2,6)*H* and SO_2ArC(2,6)*H*). All data in accordance with literature.[11]

Methyl (*E*)-2-((4-methoxyphenyl)(tosylimino)methyl)acrylate

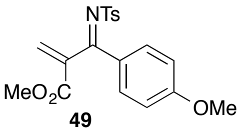

49

Following general procedure E, *N*-(4-methoxybenzylidene)-4-methylbenzenesulfonamide (1.00 g, 3.48 mmol), triphenylphosphine (184 mg, 0.70 mmol) in toluene (50 mL) and methyl propiolate (0.37 mL, 4.18 mmol) in toluene (20 mL) for 3 h at 80 °C gave, after column

chromatography (eluent: EtOAc:isohexane 5:95, R_f = 0.20) the title compound as yellow oil (910 mg, 70%); ^{1}H NMR (400 MHz, $CDCl_3$) δ_H; 2.43 (3H, s, SO_2ArC*H3*), 3.78 (3H, s, ArOCH_3), 3.85 (3H, s, CO_2CH_3), 5.87 (1H, s, =C*H*H), 6.80 (1H, s, =CH*H*), 6.88 (2H, d, *J* 9.0, Ar(3,5)*H*), 7.32 (2H, d, *J* 8.0, SO_2Ar(3,5)*H*), 7.82-7.88 (4H, m, SO_2Ar(2,6)*H* and Ar(2,6)*H*). All data in accordance with literature.[11]

Methyl 2-(4-tolyl(tosylimino)methyl)acrylate

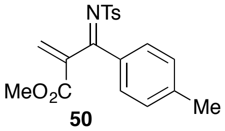

50

Following general procedure E, *N*-(4-methylbenzylidene)-4-methylbenzenesulfonamide (1.00 g, 3.69 mmol), triphenylphosphine (194 mg, 0.74 mmol) in toluene (54 mL) and methyl propiolate (0.39 mL, 4.43 mmol) in toluene (20 mL) for 3 h at 80 °C gave, after column chromatography (eluent: EtOAc:isohexane 5:95, R_f = 0.20) the title compound as yellow oil (791 mg, 60%); ^{1}H NMR (400 MHz, $CDCl_3$) δ_H; 2.39, (3H, s, ArCH_3), 2.44 (3H, s, SO_2ArC*H3*), 3.78 (3H, s, ArCH_3), 3.77 (3H, s, CO_2CH_3), 5.89 (1H, s, =C*H*H), 6.81 (1H, s, =CH*H*), 7.20 (2H, d, *J* 8.1, Ar(3,5)*H*), 7.33 (2H, d, *J* 7.9, SO_2Ar(3,5)*H*), 7.76 (2H, d, *J* 8.3, Ar(2,6)*H*), 7.88 (2H, d, *J* 8.30, SO_2Ar(2,6)*H*). All data in accordance with literature.[11]

Methyl 2-((3,5-dimethylphenyl)(tosylimino)methyl)acrylate

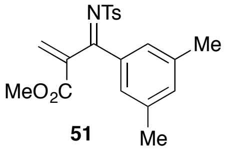

51

Following general procedure E, *N*-(3,5-dimethylbenzylidene)-4-methylbenzenesulfonamide (1.00 g, 3.51 mmol), triphenylphosphine (184 mg, 0.70 mmol) in toluene (50 mL) and methyl propiolate (0.37 mL, 4.21 mmol) in toluene (20 mL) for 3 h at 80 °C gave, after column chromatography (eluent: EtOAc:isohexane 5:95, R_f = 0.20) the title compound as yellow oil (833 mg, 64%); ^{1}H NMR (400 MHz, $CDCl_3$) δ_H; 2.31 (6H, s, Ar(3)CH_3 and Ar(5)CH_3), 2.46 (3H, s, SO_2ArCH_3), 3.78 (3H, s, CO_2CH_3), 5.87 (1H, s, =C*H*H), 6.81 (1H, s, =CH*H*), 7.19 (1H, br. s, Ar(4)*H*), 7.34 (2H, d, *J* 7.8, SO_2Ar(3,5)*H*), 7.46 (2H, br. s, Ar(2,6)*H*), 7.88 (2H, d, *J* 8.3, SO_2Ar(2,6)*H*). All data in accordance with literature.[11]

Methyl 2-(naphthalen-2-yl(tosylimino)methyl)acrylate

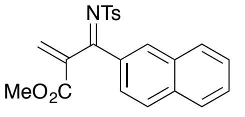

52

Following general procedure E, 4-methyl-*N*-(naphthalen-2-ylmethylene)benzenesulfonamide (1.50 g, 4.85 mmol), triphenyls phosphine (254 mg, 0.97 mmol) in toluene (79 mL) and methyl propiolate (0.52 mL, 5.82 mmol) in toluene (20 mL) for 3 h at 80 °C gave, after column chromatography (eluent: EtOAc:isohexane 5:95, R_f = 0.20) the title compound as white solid (788 mg, 42%); mp 128-131 °C ν_{max} (ATR)/cm^{-1} 1149 (S=O), 1304 (C-O), 1551 (C=N), 1736 (C=O), 2947, 3055 (C-H); ^{1}H NMR (400 MHz, $CDCl_3$) δ_H; 2.45 (3H, s, SO_2Ar(C*H₃*)), 3.79 (3H, s, CO_2CH_3), 5.99 (1H, s, =C*H*H), 6.92 (1H, s, =CH*H*), 7.36 (2H, d, *J* 8.1, SO_2Ar(3,5)*H*), 7.53 (1H, ddd, *J* 8.2, 6.9, 1.3, Ar(7)*H*), 7.60 (1H, ddd, *J* 8.2, 6.8, 1.4, Ar(8)*H*), 7.82-7.90 (3H, m, Ar(6)*H* and Ar(9)*H* and Ar(4)*H*), 7.94 (2H, d, *J* 8.3, SO_2Ar(2,6)*H*), 8.03 (1H, dd, *J* 8.8, 1.9, Ar(3)*H*), 8.27 (1H, s, Ar(1)*H*). All data in accordance with literature.[11]

General Procedure F: Isothiourea-catalysed Michael addition-lactamisation

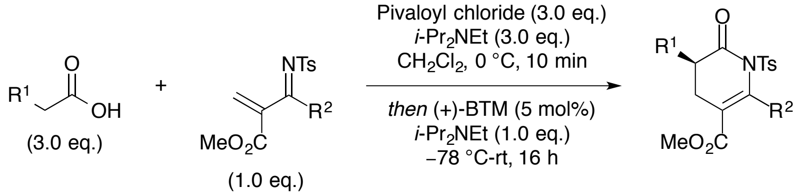

To a solution of requisite carboxylic acid (2.0 equiv) in CH_2Cl_2 (0.1 M in carboxylic acid) at 0 °C was added *i*-Pr_2NEt (3.0 equiv) and pivaloyl chloride (3.0 equiv). The reaction was left to stir for 10 min before being cooled to −78 °C at which point Lewis base catalyst (5 mol%) and Michael acceptor (1.0 equiv) and *i*-Pr_2NEt (1.0 equiv) were added and reaction was warmed to room temperature over 16 h. The reaction was quenched with HCl (1 M in H_2O), extracted with CH_2Cl_2 (×3), dried over $MgSO_4$ and concentrated under reduced pressure to give crude residue. Products were purified by column chromatography in the solvent system reported.

Methyl (*S*)-6-oxo-2,5-diphenyl-1-tosyl-1,4,5,6-tetrahydropyridine-3-carboxylate

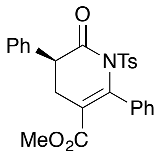

22

Following general procedure F, phenylacetic acid (37 mg, 0.27 mmol), *i*-Pr_2NEt (70 µL, 0.40 mmol) and pivaloyl chloride (49 µL, 0.40 mmol) in CH_2Cl_2 (3 mL), (−)-tetramisole **9** (3 mg, 0.013 mmol), methyl 2-(phenyl(tosylimino)methyl)acrylate **2** (50 mg, 0.13 mmol) and *i*-Pr_2NEt (23 µL, 0.13 mmol) were warmed from −78 °C to rt over 16 h to give crude product. Column chromatography (12:88 Petrol:EtOAc, R_f = 0.25) gave the title compound (44 mg,

72%) as a white solid. mp 172-174 °C; $[\alpha]_D^{20}$ −23.3 (*c* 0.15 CH_2Cl_2); Chiral HPLC analysis, Chiralpak AD-H (80:20 hexane:IPA, flow rate 1 $mLmin^{-1}$, 211 nm, 30 °C) t_R(*S*): 15.8 min, t_R(*R*): 23.0 min, 91% ee; ν_{max} (ATR) 3028, 2955 (C-H), 1711 (C=O dihydropyridone), 1701 (C=O Ester); ^{1}H NMR (500 MHz, $CDCl_3$) δ_H: 2.40 (3H, s, ArC*H*$_3$), 3.01 (1H, dd, *J* 15.2, 5.0, C(4)*H*H), 3.14 (1H, dd, *J* 15.2, 11.5, C(4)H*H*), 3.52 (3H, s, CO_2C*H*$_3$), 3.83 (1H, dd, *J* 11.4, 5.0, C(5)*H*), 7.14-7.20 (6H, m, ArC*H*), 7.26-7.40 (6H, m, ArC*H*), 7.47 (2H, d, *J* 8.0, ArC*H*); ^{13}C{^{1}H} NMR (125 MHz, $CDCl_3$) δ_C: 21.8 (Ar*C*H$_3$), 30.3 (*C*(4)), 51.3 (*C*(5)), 52.0 (CO_2*C*H$_3$), 120.7 (*C*(3)), 127.6 (Ar*C*), 128.0 (Ar*C*), 128.2 (Ar*C*), 129.0 (Ar*C*), 129.0 (Ar*C*), 129.1 (Ar*C*), 129.2 (Ar*C*), 129.6 (Ar*C*), 133.9 (4ry Ar*C*), 136.1 (Ar*C*), 136.8 (Ar*C*), 145.1 (Ar*C*), 145.3 (Ar*C*), 166.6 (*C*O$_2$CH$_3$), 173.4 (*C*(6)); HRMS (NSI^+) $C_{26}H_{24}NO_5S$ $[M+H]^+$, found 462.1369, requires 462.1370 (−0.2 ppm).

Methyl (*S*)-5-(4-methoxyphenyl)-6-oxo-2-phenyl-1-tosyl-1,4,5,6-tetrahydropyridine-3-carboxylate

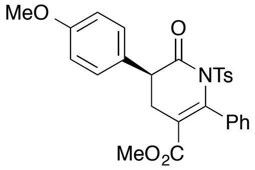

23

Following general procedure F, 4-methoxyphenylacetic acid (45 mg, 0.27 mmol), *i*-Pr_2NEt (70 µL, 0.40 mmol), pivaloyl chloride (49 µL, 0.40 mmol) in CH_2Cl_2 (3 mL), (−)-tetramisole **9** (3 mg, 0.013 mmol), methyl 2-(phenyl(tosylimino)methyl)acrylate **2** (50 mg, 0.13 mmol) and *i*-Pr_2NEt (23 µL, 0.13 mmol) were warmed from −78 °C to rt over 16 h to give crude product. Column chromatography (15:75 Petrol:EtOAc, R_f = 0.25) to give the title compound (50 mg, 76%) as a colourless oil; $[\alpha]_D^{20}$ −31.6 (*c* 0.25 CH_2Cl_2); Chiral HPLC analysis, Chiralpak OD-H (80:20 hexane:IPA, flow rate 1 $mLmin^{-1}$, 211 nm, 30 °C) t_R(*S*): 12.7 min, t_R(*R*): 25.4 min, 95% ee; ν_{max} (ATR) 2951, 2918 (C-H), 1719 (C=O dihydropyridone), 1699 (C=O Ester); ^{1}H NMR (500 MHz, $CDCl_3$) δ_H: 2.39 (3H, s, SO_2ArC*H*$_3$), 2.98 (1H, dd, *J* 15.2, 5.0 C(4)*H*H), 3.10 (1H, dd, *J* 15.2, 11.6 C(4)H*H*), 3.52 (3H, s, CO_2C*H*$_3$), 3.76-3.79 (4H, m, C(5)*H* and ArOC*H*$_3$), 6.87 (2H, d, *J* 8.7, C(5)Ar(3,5)*H*), 7.09 (2H, d, *J* 8.7, SO_2Ar(3,5)*H*), 7.14 (2H, d, *J* 8.1, C(2)Ar(3,5)*H*), 7.18-7.19 (2H, m, C(2)Ar(2,6)*H*), 7.28 (2H, d, *J* 7.8, C(5)Ar(2,6)*H*), 7.37 (1H, t, *J* 7.41, C(2)Ar(4)*H*), 7.46 (2H, d, *J* 8.4, SO_2Ar(2,6)*H*); ^{13}C{^{1}H} NMR (125 MHz, $CDCl_3$) δ_C: 21.8 (SO_2Ar*C*H$_3$), 30.4 (*C*(4)HH), 50.6 (*C*(5)H), 52.1 (CO_2*C*H$_3$), 55.4 (ArO*C*H$_3$), 114.4 (C(5)Ar*C*(3,5)H), 120.8 (*C*(3)), 127.6 (C(5)Ar*C*(2,6)H), 128.9 (C(5)Ar*C*(1)), 129.1 (SO_2Ar*C*(2,6)H), 129.1 (C(2)Ar*C*(3,5)H), 129.2 (C(2)Ar*C*(4)H), 129.3 (SO_2Ar*C*(3,5)H), 129.6 (C(2)Ar*C*(2,6)H), 133.9 (C(2)Ar*C*(1)), 136.2 (SO_2Ar*C*(4)),

145.1 (C(2)), 145.2 (SO_2ArC(1)), 159.2 (C(5)ArC(4)), 166.7 (CO$_2$Me), 173.7 (C(6)); HRMS (NSI^+) $C_{26}H_{24}NO_6S$ $[M+H]^+$, found 492.1466, requires 492.1475 (−1.9 ppm).

Methyl (*S*)-5-(4-(dimethylamino)phenyl)-6-oxo-2-phenyl-1-tosyl-1,4,5,6-tetrahydropyridine-3-carboxylate

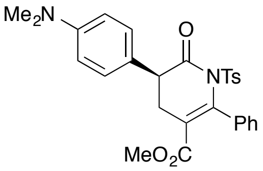

24

Following general procedure F, 4-(dimethylamino)phenyl acetic acid (104 mg, 0.58 mmol), *i*-Pr_2NEt (152 µL, 0.87 mmol), pivaloyl chloride (107 µL, 0.87 mmol) in CH_2Cl_2 (5 mL), (−)-tetramisole (4 mg, 0.015 mmol), methyl 2-(phenyl(tosylimino)methyl)acrylate **2** (100 mg, 0.29 mmol) and *i*-Pr_2NEt (51 µL, 0.29 mmol) were warmed −78 °C to rt over 16 h to give crude product. Column chromatography (12:88 Petrol:EtOAc, R_f = 0.25) to give the title compound (100 mg, 68%) as a white solid; mp 164-166 °C : $[\alpha]_D^{20}$ −27.1 (c 0.25 CH_2Cl_2); Chiral HPLC analysis, Chiralpak AD-H (80:20 hexane:IPA, flow rate 1 mLmin^{-1}, 254 nm, 30 °C) $t_R(S)$: 23.4 min, $t_R(R)$: 26.2 min, 94% ee; ν_{max} (ATR) 2883 (C-H), 1722(C=O dihydropyridinone), 1690 (C=O Ester) 1155 (C-O ester); ^{1}H NMR (400 MHz, $CDCl_3$) δ_H: 2.39 (3H, s, SO_2ArCH_3), 2.93-3.00 (7H, m, ArN(CH_3)$_2$ and C(4)HH), 3.10 (1H, dd, J 15.2, 11.3, C(4)HH), 3.52 (3H, s, CO_2CH_3), 3.73 (1H, dd, J 11.1, 5.0, C(5)H), 6.68 (2H, d, J 8.6, C(5)Ar(3,5)H), 7.03 (2H, J 8.59, SO_2Ar(3,5)H), 7.13 (2H, d, J 8.2, C(2)Ar(3,5)H), 7.19 (2H, d, J 7.3, C(5)Ar(2,6)H), 7.26-7.29 (2H, m, C(2)Ar(3,5)H), 7.37 (1H, t, J 7.32, C(2)Ar(4)H), 7.47 (2H, d, J 8.3, SO_2Ar(2,6)H); ^{13}C{^{1}H} NMR (125 MHz, $CDCl_3$) δ_C: 21.8 (SO_2ArCH$_3$), 30.3 (C(4)HH), 40.7 (ArN(CH$_3$)$_2$), 50.5 (C(5)H), 52.0 ($CO_2$$CH_3$), 112.9 (C(5)Ar$C$(3,5)H), 120.9 ($C$(3)), 124.3 (C(5)Ar$C$(1)), 127.6 (C(2)Ar$C$(3,5)H), 128.8 ($SO_2ArC$(3,5)H), 129.0 (C(2)Ar$C$(4)H), 129.1 (C(2)Ar$C$(3,5)H), 129.1 ($SO_2ArC$(2,6)H), 129.5 (C(5)Ar$C$(2,6)H), 134.1 ($SO_2ArC$(4)), 136.3 (C(2)$C$(1)), 145.0 ($C$(2)), 145.1 ($SO_2ArC$(1)), 150.2 (C(5)Ar$C$(4)NMe$_2$), 166.8 ($CO_2$Me), 174.0 ($C$(6)); HRMS ($NSI^+$) $C_{28}H_{29}N_2O_5S$ $[M+H]^+$, found 505.1780, requires 505.1792 (−2.3 ppm).

Methyl (*S*)-6-oxo-2-phenyl-1-tosyl-5-(4-(trifluoromethyl)phenyl)-1,4,5,6-tetrahydropyridine-3-carboxylate

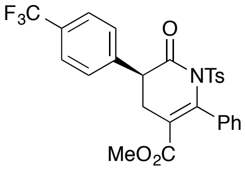

25

Following general procedure F, phenylacetic acid (45 mg, 0.27 mmol), *i*-Pr_2NEt (70 µL, 0.40 mmol), pivaloyl chloride (49 µL, 0.40 mmol) in CH_2Cl_2 (3 mL), (−)-tetramisole **9** (3 mg, 0.013 mmol), methyl 2-(phenyl(tosylimino)methyl)acrylate **2** (50 mg, 0.13 mmol) and *i*-Pr_2NEt (23 µL, 0.13 mmol) were warmed from −78 °C to rt over 16 h to give crude product. Column chromatography (15:75 Petrol:EtOAc, R_f = 0.25) to give the title compound (50 mg, 76%) as a white solid: mp 138-140 °C; $[\alpha]_D^{20}$ −66.0 (*c* 0.10 CH_2Cl_2); Chiral HPLC analysis, Chiralpak AD-H (80:20 hexane:IPA, flow rate 1 mLmin^{-1}, 211 nm, 30 °C) t_R(*R*): 17.2 min, t_R(*S*): 21.5 min, 97% ee; ν_{max} (ATR) 2957, 2930 (C-H), 1730 (C=O dihydropyridone), 1721 (C=O Ester); ^{1}H NMR (500 MHz, $CDCl_3$) δ_H: 2.40 (3H, s, SO_2ArCH_3), 3.02 (1H, dd, *J* 15.2, 5.0 C(4)*H*H), 3.14 (1H, dd, *J* 15.1, 12.1 C(4)H*H*), 3.53 (3H, s, CO_2CH_3), 3.89 (1H, dd, *J* 11.9, 4.9, C(5)*H*), 7.15 (2H, d, *J* 8.2, SO_2Ar(3,5)*H*), 7.19 (2H, d, *J* 7.4, C(5)Ar(2,6)*H*), 7.26-7.32 (4H, m, C(2)Ar(3,5)*H* and C(2)Ar(2,6)*H*), 7.39 (1H, t, *J* 7.4, C(2)Ar(4)*H*), 7.45 (2H, d, *J* 8.3, SO_2Ar(2,6)*H*), 7.61 (2H, d, *J* 8.0, C(5)Ar(3,5)*H*); ^{13}C{^{1}H} NMR (100 MHz, $CDCl_3$) δ_C: 21.8 (SO_2ArCH_3), 30.2 (*C*(4)HH), 51.2 (*C*(5)H), 52.1 (CO_2CH_3), 120.5 (*C*(3)), 124.0 (q, $^1J_{CF}$ 271, Ar*C*F_3), 125.9 (q, $^3J_{CF}$ 2.75, C(5)Ar*C*(3,5)H), 127.7 (C(2)Ar*C*(3,5)H), 128.8 (C(2)Ar*C*(2,6)H), 129.1 (SO_2Ar*C*(2,6)H), 129.2 (C(5)Ar*C*(2,6)H), 129.3 (C(2)Ar*C*(4)H), 129.6 (SO_2Ar*C*(3,5)H), 130.3 (q, $^2J_{CF}$ 32.3, C(5)Ar*C*(4)), 133.7 (C(2)Ar*C*(1)), 136.0 (SO_2Ar*C*(4)), 140.8 (C(5)Ar*C*(1)), 145.4 (*C*(2)), 145.6 (SO_2Ar*C*(1)), 166.4 (*C*O_2Me), 172.7 (*C*(6)); ^{19}F{^{1}H} (470 MHz) −62.8 (ArC*F₃*); HRMS (NSI$^+$) $C_{27}H_{23}NO_5F_3S$ [M+H]$^+$, found 530.1235, requires 530.1244 (−1.6 ppm).

Methyl (*S*)-6-oxo-2-phenyl-5-(*m*-tolyl)-1-tosyl-1,4,5,6-tetrahydropyridine-3-carboxylate

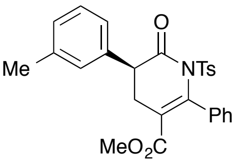

26

Following general procedure F, *m*-tolylacetic acid (40 mg, 0.27 mmol), *i*-Pr_2NEt (70 µL, 0.40 mmol), pivaloyl chloride (49 µL, 0.40 mmol), in CH_2Cl_2 (3 mL), (−)-tetramisole **9** (3 mg, 0.013 mmol), methyl 2-(phenyl(tosylimino)methyl)acrylate **2** (50 mg, 0.13 mmol) and *i*-

Pr_2NEt (23 μL, 0.13 mmol) were warmed −78 °C to rt over 16 h to give crude product. Column chromatography (12.5:87.5 Petrol:EtOAc, $R_f = 0.25$) to give the title compound (44 mg, 69%) as a colourless oil; $[\alpha]_D^{20}$ −32.1 (*c* 0.10 CH_2Cl_2); Chiral HPLC analysis, Chiralpak AD-H (80:20 hexane:IPA, flow rate 1 mLmin^{-1}, 211 nm, 30 °C) t_R(*S*): 14.7 min, t_R(*R*): 28.7 min, 95% ee; ν_{max} (ATR) 2953 (C-H), 1717 (C=O dihydropyridinone), 1703 (C=O Ester); ^{1}H NMR (500 MHz, $CDCl_3$) δ_H: 2.32 (3H, s, C(5)ArC*H₃*), 2.40 (3H, s, SO_2ArC*H*$_3$), 2.98 (1H, dd, *J* 15.2, 5.0 C(4)*H*H), 3.12 (1H, dd, *J* 15.2, 11.8 C(4)H*H*), 3.52 (3H, s, CO_2C*H*$_3$), 3.78 (1H, dd, *J* 11.7, 5.0, C(5)*H*), 6.93-6.97 (2H, m, C(5)Ar(2)*H* and C(5)Ar(4)*H*), 7.10 (1H, d, *J* 7.6, C(5)Ar(6)*H*), 7.16 (2H, d, *J* 8.2, SO_2Ar(3,5)*H*), 7.20-7.24 (3H, m, C(5)Ar(5)*H* and C(2)Ar(3,5)*H*), 7.27-7.30 (2H, m, C(2)Ar(2,6)*H*), 7.38 (1H, t, *J* 7.4, C(2)Ar(4)*H*), 7.50 (2H, d, *J* 8.4, SO_2Ar(2,6)*H*); ^{13}C{^{1}H} NMR (125 MHz, $CDCl_3$) δ_C: 21.7 (C(5)Ar*C*H$_3$), 21.8 (SO_2Ar*C*H$_3$), 30.4 (*C*(4)HH), 51.3 (*C*(5)H), 52.0 (CO_2*C*H$_3$), 120.7 (*C*(3)), 125.3 (C(5)Ar*C*(2)H), 127.6 (C(2)Ar*C*(2,6)H), 128.8 (C(5)Ar*C*(4)H), 128.9 (C(5)Ar*C*(6)H), 128.9 (C(5)Ar*C*(5)H), 129.1 (SO_2Ar*C*(2,6)H), 129.1 (C(2)Ar*C*(4)H), 129.2 (SO_2Ar*C*(3,5)H), 129.5 (C(2)Ar*C*(3,5)H), 134.0 (C(5)Ar*C*(3)), 136.2 (SO_2Ar*C*(4)), 136.8 (C(2)Ar*C*(1)), 138.6 (C(5)Ar*C*(1)), 145.1 (*C*(2)), 145.3 (SO_2Ar*C*(1)), 166.7 (*C*O_2Me), 173.5 (*C*(6)); HRMS (NSI$^+$) $C_{27}H_{26}NO_5S$ $[M+H]^+$, found 476.1517, requires 476.1526 (−1.9 ppm).

Methyl (*S*)-5-(4-bromophenyl)-6-oxo-2-phenyl-1-tosyl-1,4,5,6-tetrahydropyridine-3-carboxylate

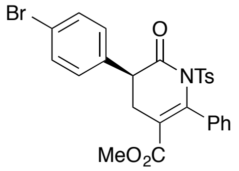

27

Following general procedure F, 4-bromophenylacetic acid (86 mg, 0.40 mmol), *i*-Pr_2NEt (105 μL, 0.60 mmol), pivaloyl chloride (74 μL, 0.60 mmol) in CH_2Cl_2 (5 mL), (−)-tetramisole **9** (3 mg, 0.013 mmol), methyl 2-(phenyl(tosylimino)methyl)acrylate **2** (75 mg, 0.20 mmol) and *i*-Pr_2NEt (35 μL, 0.20 mmol) were warmed −78 °C to rt over 16 h to give crude product. Column chromatography (10:90 Petrol:EtOAc, $R_f = 0.20$) to give the title compound (44 mg, 69%) as a white solid: mp 136-138 °C; $[\alpha]_D^{20}$ −12.0 (*c* 0.1 CH_2Cl_2); Chiral HPLC analysis, Chiralpak AD-H (80:20 hexane:IPA, flow rate 1 mLmin^{-1}, 254 nm, 30 °C) t_R(*R*): 23.3 min, t_R(*S*): 28.3 min, 72% ee; ν_{max} (ATR) 2949 (C-H), 1730 (C=O dihydropyridinone), 1730 (C=O Ester) 1138 (C-O ester); ^{1}H NMR (500 MHz, $CDCl_3$) δ_H: 2.40 (3H, s, SO_2ArC*H*$_3$), 2.99 (1H, dd, *J* 15.2, 5.1 C(4)*H*H), 3.09 (1H, dd, *J* 15.2, 11.7 C(4)H*H*), 3.52 (3H, s, CO_2C*H*$_3$), 3.78 (1H, dd, *J* 11.7, 5.1, C(5)*H*), 7.05 (2H, d, *J* 8.4, C(5)Ar(2,5)*H*), 7.15 (2H, d, *J* 8.2, SO_2Ar(3,5)*H*), 7.18 (2H, d, *J* 7.2, C(2)Ar(3,5)*H*), 7.29 (2H, d, *J* 7.9, C(2)Ar(2,6)*H*), 7.38 (1H, t, *J* 7.5,

C(2)Ar(4)*H*), 7.44-7.48 (4H, m, SO_2Ar(2,6)*H* and C(5)Ar(3,5)*H*); ^{13}C NMR (125 MHz, $CDCl_3$) 21.8 (SO_2Ar*C*H_3), 30.1 (*C*(4)HH), 50.9 (*C*(5)H), 52.1 (CO_2*C*H_3), 120.5 (*C*(3)), 122.1 (C(5)Ar*C*(4)Br), 127.6 (C(2)Ar*C*(2,6)H), 129.1 (SO_2Ar*C*(2,6)H), 129.2 (SO_2Ar*C*(3,5)H), 129.2 (C(2)Ar*C*(4)H), 129.6 (C(2)Ar*C*(3,5)H), 130.0 (C(5)Ar*C*(3,5)H), 132.2 (C(5)Ar*C*(3,5)H), 133.7 (SO_2Ar*C*(4)), 135.8 (C(2)Ar*C*(1)), 136.0 (C(5)Ar*C*(1)), 145.3 (*C*(2)), 145.4 (SO_2Ar*C*(1)), 166.5 (*C*O_2Me), 172.9 (*C*(6)); HRMS (NSI^+) $C_{26}H_{22}Br^{79}O_5SNa$ $[M+Na]^+$, found 562.0283, requires 562.0294 (−2.0 ppm).

Methyl (*S*)-6-oxo-2-phenyl-5-(thiophen-3-yl)-1-tosyl-1,4,5,6-tetrahydropyridine-3-carboxylate

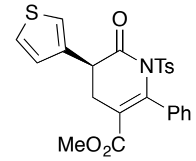

28

Following general procedure F, 3-thiophene acetic acid (57 mg, 0.40 mmol), *i*-Pr_2NEt (105 µL, 0.60 mmol), pivaloyl chloride (74 µL, 0.60 mmol) in CH_2Cl_2 (5 mL), (−)-tetramisole **9** (3 mg, 0.013 mmol), methyl 2-(phenyl(tosylimino)methyl)acrylate **2** (75 mg, 0.20 mmol) and *i*-Pr_2NEt (35 µL, 0.20 mmol) were warmed −78 °C to rt over 16 h to give crude product. Column chromatography (10:90 Petrol:EtOAc, R_f = 0.19) to give the title compound (61 mg, 65%) as a white solid: mp 159-152 °C; $[\alpha]_D^{20}$ −28.0 (*c* 0.1 CH_2Cl_2); Chiral HPLC analysis, Chiralpak IB (92.5:7.5 hexane:IPA, flow rate 1.5 mLmin^{-1}, 220 nm, 30 °C) t_R(*S*): 13.9 min, t_R(*R*): 15.8 min, 91% ee; ν_{max} (ATR) 3096, 2957 (C-H), 1720 (C=O dihydropyridone), 1699 (C=O Ester) 1150 (C-O); ^{1}H NMR (400 MHz, $CDCl_3$) δ_H: 2.39 (3H, s, SO_2ArC*H_3*), 3.05-3.16 (2H, m, C(4)*H*H and C(4)H*H*), 3.52 (3H, s, CO_2C*H_3*), 4.00 (1H, dd, *J* 8.6, 5.6, C(5)*H*), 6.98 (1H, dd, *J* 5.0, 1.3, C(5)Ar(4)*H*), 7.12-7.15 (5H, m, SO_2Ar(3,5)*H* and C(2)Ar(3,5)*H* and C(5)Ar(2)*H*), 7.24-7.27 (2H, m, C(2)Ar(2,6)*H*), 7.30 (1H, dd, *J* C(5)Ar(5)*H*), 7.36 (1H, tt, *J* 6.8, 1.2, C(2)Ar(4)*H*), 7.45 (2H, d, *J* 8.4, SO_2Ar(2,6)*H*); ^{13}C NMR (125 MHz, $CDCl_3$) 21.8 (SO_2Ar*C*H_3), 29.3 (*C*(4)HH), 46.6 (*C*(5)H), 52.1 (CO_2*C*H_3), 120.1 (*C*(3)), 122.8 (C(5)Ar*C*(2)H), 126.6 (C(5)Ar*C*(5)H), 127.1 (C(5)Ar*C*(4)H), 127.6 (C(2)Ar*C*(2,6)H), 129.0 (SO_2Ar*C*(2,6)H), 129.1 (C(2)Ar*C*(4)H), 129.2 (SO_2Ar*C*(3,5)H), 129.6 (C(2)Ar*C*(3,5)H), 133.9 (SO_2Ar*C*(4)), 136.1 (C(2)Ar*C*(1)), 136.2 (C(5)*C*(3)), 145.2 (*C*(2)), 145.2 (SO_2Ar*C*(1)), 166.7 (*C*O_2Me), 172.4 (*C*(6)); HRMS (NSI^+) $C_{24}H_{21}NO_5S_2Na$ $[M+Na]^+$, found 490.0739, requires 490.0753 (−2.9 ppm).

Methyl (*S*)-2-(4-methoxyphenyl)-6-oxo-5-phenyl-1-tosyl-1,4,5,6-tetrahydropyridine-3-carboxylate

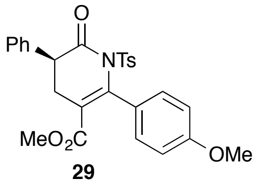

29

Following general procedure F, phenylacetic acid (74 mg, 0.54 mmol), *i*-Pr_2NEt (141 μL, 0.81 mmol), pivaloyl chloride (100 μL, 0.81 mmol) in CH_2Cl_2 (5 mL), (−)-tetramisole **9** (3 mg, 0.014 mmol), methyl (*E*)-2-((4-methoxyphenyl)(tosylimino)methyl)acrylate **49** (100 mg, 0.27 mmol) and *i*-Pr_2NEt (47 μL, 0.27 mmol) were warmed −78 °C to rt over 16 h to give crude product. Column chromatography (12.5:87.5 Petrol:EtOAc, R_f = 0.25) to give the title compound (44 mg, 69%) as a colourless oil: $[\alpha]_D^{20}$ −16.0 (*c* 0.25 CH_2Cl_2); Chiral HPLC analysis, Chiralpak OD-H (80:20 hexane:IPA, flow rate 1 mLmin^{-1}, 254 nm, 30 °C) t_R(*S*): 15.5 min, t_R(*R*): 25.8 min, 98% ee; ν_{max} (ATR) 3001 (C-H), 1727 (C=O dihydropyridone), 1705 (C=O Ester); ^{1}H NMR (500 MHz, $CDCl_3$) δ_H: 2.40 (3H, s, SO_2ArCH_3), 2.99 (1H, dd, *J* 15.2, 5.0 C(4)*H*H), 3.10 (1H, dd, *J* 15.2, 11.6 C(4)H*H*), 3.55 (3H, s, CO_2CH_3), 3.80 (1H, dd, *J* 11.5, 5.0, C(5)*H*), 3.85 (3H, s, $ArOCH_3$), 6.80 (2H, d, *J* 8.8, C(2)Ar(3,5)*H*), 7.12-7.18 (6H, m, SO_2Ar(3,5)*H*, C(5)Ar(3,5)*H* and C(5)Ar(2,6)*H*), 7.28-7.34 (3H, m, C(2)Ar(2,6)*H* and C(5)Ar(4)*H*), 7.54 (2H, d, *J* 8.4, SO_2Ar(2,6)*H*); ^{13}C NMR (125 MHz, $CDCl_3$) δ_C: 21.8 (SO_2ArCH_3), 30.4 (*C*(4)HH), 51.6 (*C*(5)H), 52.1 (CO_2CH_3), 55.4 ($ArOCH_3$), 113.1 (C(2)Ar*C*(3,5)), 119.9 (*C*(3)), 126.3 (C(2)Ar*C*(1)), 128.0 (C(5)Ar*C*(4)H), 128.3 (C(5)Ar*C*(3,5)H), 129.0 (SO_2Ar*C*(2,6)H), 129.1 (C(5)Ar*C*(2,6)H), 129.2 (C(2)Ar*C*(2,6)H), 131.0 (SO_2Ar*C*(3,5)H), 136.4 (SO_2Ar*C*(4)), 137.0 (C(5)Ar*C*(1)), 145.1 (*C*(2)), 145.5 (SO_2Ar*C*(1)), 160.3 (C(2)Ar*C*(4)), 166.8 (*C*O_2Me), 173.5 (*C*(6)); HRMS (NSI^+) $C_{27}H_{26}NO_6S$ $[M+H]^+$, found 492.1467, requires 492.1475 (−1.7 ppm).

Methyl (*S*)-6-oxo-5-phenyl-2-(*p*-tolyl)-1-tosyl-1,4,5,6-tetrahydropyridine-3-carboxylate

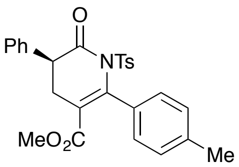

30

Following general procedure F, phenylacetic acid (76 mg, 0.56 mmol), *i*-Pr_2NEt (146 μL, 0.84 mmol), pivaloyl chloride (103 μL, 0.84 mmol) in CH_2Cl_2 (5 mL), (−)-tetramisole **9** (3 mg, 0.014 mmol), methyl 2-((4-methylphenyl)(tosylimino)methyl)acrylate **50** (100 mg, 0.28 mmol) and *i*-Pr_2NEt (49 μL, 0.28 mmol) were warmed −78 °C to rt over 16 h to give crude product. Column chromatography (15:85 Petrol:EtOAc, R_f = 0.25) to give the title compound

(79 mg, 59%) as a white solid: mp 134-136 °C; $[\alpha]_D^{20}$ −14.8 (*c* 0.25 CH_2Cl_2); Chiral HPLC analysis, Chiralpak AD-H (80:20 hexane:IPA, flow rate 1 $mLmin^{-1}$, 211 nm, 30 °C) t_R(*R*): 13.3 min, t_R(*S*): 25.6 min, 90% ee; ν_{max} (ATR) 3032, 2953 (C-H), 1738 (C=O dihydropyridinone), 1717 (C=O Ester); ^{1}H NMR (500 MHz, $CDCl_3$) δ_H: 2.56 (3H, s, SO_2ArC*H*$_3$), 2.57 (3H, s, C(2)ArC*H*$_3$), 3.15 (1H, dd, *J* 15.2, 5.0 C(4)*H*H), 3.27 (1H, dd, *J* 15.2, 11.6 C(4)H*H*), 3.71 (3H, s, CO_2C*H*$_3$), 3.97 (1H, dd, *J* 11.5, 5.0, C(5)*H*), 7.26-7.33 (6H, m, SO_2Ar(3,5)*H* and C(2)Ar(3,5)*H*), 7.42-7.51 (5H, m, C(5)Ar(3,5)*H* and C(5)Ar(2,6)*H* and C(5)Ar(5)*H*), 7.68 (2H, d, *J* 8.4, SO_2Ar(2,6)*H*); ^{13}C NMR (125 MHz, $CDCl_3$) δ_C: 21.7 (C(2)Ar*C*H$_3$), 21.8 (SO_2Ar*C*H$_3$), 30.4 (*C*(4)HH), 51.4 (*C*(5)H), 52.1 (CO_2*C*H$_3$), 120.2 (*C*(3)), 128.0 (C(5)Ar*C*(4)H), 128.3 (C(2)Ar*C*(3,5)H), 128.4 (SO_2Ar*C*(3,5)H), 129.0 (C(5)Ar*C*(3,5)H), 129.1 (SO_2Ar*C*(2,6)H), 129.2 (C(2)Ar*C*(2,6)H), 129.4 (C(5)Ar*C*(2,6)H), 131.1 (C(2)Ar*C*(1)), 136.3 (SO_2Ar*C*(4)), 136.9 (C(2)Ar*C*(4)), 139.2 (C(5)Ar*C*(1)), 145.1 (*C*(2)), 145.6 (SO_2Ar*C*(1)), 166.7 (*C*O$_2$Me), 173.4 (*C*(6)); HRMS (NSI$^+$) $C_{27}H_{25}NO_5SNa$ $[M+Na]^+$, found 498.1338, requires 498.1346 (−1.5 ppm).

Methyl (*S*)-2-(3,5-dimethylphenyl)-6-oxo-5-phenyl-1-tosyl-1,4,5,6-tetrahydropyridine-3-carboxylate

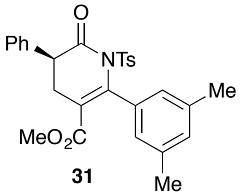

31

Following general procedure F, phenylacetic acid (74 mg, 0.54 mmol), *i*-Pr$_2$NEt (141 μL, 0.81 mmol), pivaloyl chloride (100 μL, 0.81 mmol) in CH_2Cl_2 (5 mL), (−)-tetramisole **9** (3 mg, 0.014 mmol), methyl 2-((3,5-dimethylphenyl)(tosylimino)methyl)acrylate **51** (100 mg, 0.27 mmol) and *i*-Pr$_2$NEt (47 μL, 0.27 mmol) were warmed −78 °C to rt over 16 h to give crude product. Column chromatography (10:90 Petrol:EtOAc, R_f = 0.19) to give the title compound (44 mg, 69%) as a white solid: mp 182-184 °C; $[\alpha]_D^{20}$ −15.0 (*c* 0.1 CH_2Cl_2); Chiral HPLC analysis, Chiralpak AD-H (90:10 hexane:IPA, flow rate 1 $mLmin^{-1}$, 211 nm, 30 °C) t_R(*R*: 11.8 min, t_R(*S*): 17.6 min, 97% ee; ν_{max} (ATR) 2950 (C-H), 1730 (C=O dihydropyridone), 1700 (C=O Ester) 1150 (C-O ester); ^{1}H NMR (400 MHz, $CDCl_3$) δ_H: 2.22 (6H, s, C(2)Ar(3,5)C*H*$_3$) 2.40 (3H, s, SO_2ArC*H*$_3$), 2.96 (1H, dd, *J* 15.2, 5.1 C(4)*H*H), 3.12 (1H, dd, *J* 15.2, 11.9 C(4)H*H*), 3.54 (3H, s, CO_2C*H*$_3$), 3.80 (1H, dd, *J* 11.9, 5.0, C(5)*H*), 6.74 (2H, br. s, C(2)Ar(2,6)*H*), 6.98 (1H, br. s, C(2)Ar(4)*H*), 7.14-7.20 (4H, m, SO_2Ar(3,5)*H* and C(5)Ar(3,5)*H*), 7.28-7.37 (3H, m, C(2)Ar(2,6)*H* and C(5)Ar(4)*H*), 7.50 (2H, d, *J* 8.4, SO_2Ar(2,6)*H*); ^{13}C NMR (125 MHz, $CDCl_3$) 21.3 (C(2)Ar(3,5)*C*H$_3$), 21.8 (SO_2Ar*C*H$_3$), 30.7 (*C*(4)HH), 51.7 (*C*(5)H), 52.0 (CO_2*C*H$_3$), 120.4 (*C*(3)), 127.3 (C(2)Ar*C*(2,6)H), 128.0

(C(5)Ar*C*(3,5)H), 128.4 (SO_2Ar*C*(3,5)H), 129.0 (C(5)Ar*C*(4)H), 129.1 (C(5)Ar*C*(2,6)H), 129.1 (SO_2Ar*C*(2,6)H), 130.9 (C(2)Ar*C*(4)H), 133.4 (SO_2Ar*C*(4)), 136.3 (C(5)Ar*C*(1)), 137.0 (C(2)Ar*C*(1)), 137.1 (C(2)Ar*C*(3,5)), 145.0 (*C*(2)), 145.4 (SO_2Ar*C*(1)), 166.9 (*C*O_2Me), 173.7 (*C*(6)); HRMS (NSI^+) $C_{28}H_{26}NO_5SNa$ $[M+Na]^+$, found 512.1488, requires 512.1502 (−2.8 ppm).

Methyl (*S*)-2-(naphthalen-2-yl)-6-oxo-5-phenyl-1-tosyl-1,4,5,6-tetrahydropyridine-3-carboxylate

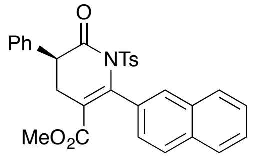

32

Following general procedure F, phenylacetic acid (68 mg, 0.50 mmol), *i*-Pr_2NEt (131 µL, 0.75 mmol), pivaloyl chloride (93 µL, 0.75 mmol) in CH_2Cl_2 (5 mL), (−)-tetramisole **9** (3 mg, 0.014 mmol), Methyl 2-(naphthalen-2-yl(tosylimino)methyl)acrylate **52** (100 mg, 0.25 mmol) and *i*-Pr_2NEt (44 µL, 0.25 mmol) were warmed −78 °C to rt over 16 h to give crude product. Column chromatography (15:85 Petrol:EtOAc, R_f = 0.23) to give the title compound (88 mg, 69%) as a white solid: mp 157-159 °C; $[\alpha]_D^{20}$ +46.0 (*c* 0.25 CH_2Cl_2); Chiral HPLC analysis, Chiralpak AD-H (80:20 hexane:IPA, flow rate 1 mLmin^{-1}, 254 nm, 30 °C) t_R(*R*): 15.3 min, t_R(*S*): 23.7 min, 91% ee; ν_{max} (ATR) 3037, 2952 (C-H), 1735 (C=O dihydropyridone), 1718 (C=O Ester); ^1H NMR (400 MHz, $CDCl_3$) δ_H: 2.31 (3H, s, SO_2ArC*H*$_3$), 3.08 (1H, dd, *J* 15.2, 5.1 C(4)*H*H), 3.20 (1H, dd, *J* 15.2, 11.6 C(4)H*H*), 3.50 (3H, s, CO_2C*H*$_3$), 3.89 (1H, dd, *J* 11.5, 5.1, C(5)*H*), 6.91 (2H, d, *J* 8.1, Ar*H*), 7.22-7.26 (2H, m, Ar*H*), 7.29-7.38 (6H, m, Ar*H*), 7.44-7.55 (4H, m, Ar*H*), 7.80 (1H, d, *J* 8.5, Ar*H*). 7.86 (1H, d, *J* 8.0, Ar*H*); ^{13}C NMR (125 MHz, $CDCl_3$) δ_C: 21.7 (SO_2ArCH_3), 30.5 (*C*(4)HH), 51.6 (*C*(5)H), 52.1 (CO_2CH_3), 120.7 (*C*(3)), 126.3 (Ar*C*H), 127.0 (Ar*C*H), 127.0 (Ar*C*H), 127.9 (Ar*C*H), 127.9 (Ar*C*H), 128.1 (Ar*C*H), 128.1 (Ar*C*H), 128.3 (Ar*C*H), 128.4 (Ar*C*), 128.9 (Ar*C*H), 129.1 (Ar*C*H), 131.4 (Ar*C*), 132.4 (Ar*C*), 133.4 (Ar*C*), 136.2 (Ar*C*), 137.0 (Ar*C*), 145.1 (*C*(2)), 145.7 (SO_2Ar*C*(1)), 166.5 (*C*O_2Me), 173.5 (*C*(2)); HRMS (NSI^+) $C_{30}H_{25}NO_5SNa$ $[M+Na]^+$, found 534.1339, requires 534.1346 (−1.2 ppm).

References:

[1] L. C. Morrill, L. A. Ledingham, J-P. Couturier, J. Bickel, A. D. Harper, C. Fallan, A. D. Smith, *Org. Biomol. Chem.* **2014**, *12*, 624-636.

[2] W. J. L. Wood, A. W. Patterson, H. J. Hirojuki, K. Rishi, J. A. Ellman, *J. Am. Chem. Soc.* **2005**, *127*, 15521.

[3] N. J. Turro, G. C. Weed, *J. Am. Chem. Soc.* **1983**, *105*, 1861-1868

[4] P. Borowiecki, M. Bretner, *Tetrahedron: Asymmetry* **2013**, *24*, 925-936

[5] P. Baburajan, K. P. Elango, *Tetrahedron Lett.* **2014**, *55*, 3525-3528

[6] V. Gasparotto, I. Castagliuolo, G. Chlarelotto, V. Pezzi, D. Montanaro, P. Brun, G. Palù, G. Viola, M. G. Ferlin, *J. Med. Chem.* **2006**, *49*, 1910-1915

[7] M. A. Ariger, E. M. Carreira, *Org. Lett.* **2012**, *14*, 4522-4524

[8] A. Bugarin, B. T. Connell and K. D. Jones, *Chem. Commun*. **2010**, *46*, 1715-1717

[9] L. H, Merwin, W. P. Norris, G. S. Ostrom and R. D. Gilardi, *J. Org. Chem.* **1997**, *62*, 9070-9075

[10] C. De Fusco, T. Fuoco, G. Croce, A. Lattanzi, *Org. Lett.* **2012**, *14*, 4078-4081

[11] D. G. Stark, T. J. C. O'Riordan, A. D. Smith, *Org. Lett.* **2014**, *16*, 6496-6499

4.0 ^{1}H NMR and {^{1}H}^{13}C NMR spectra for novel compounds

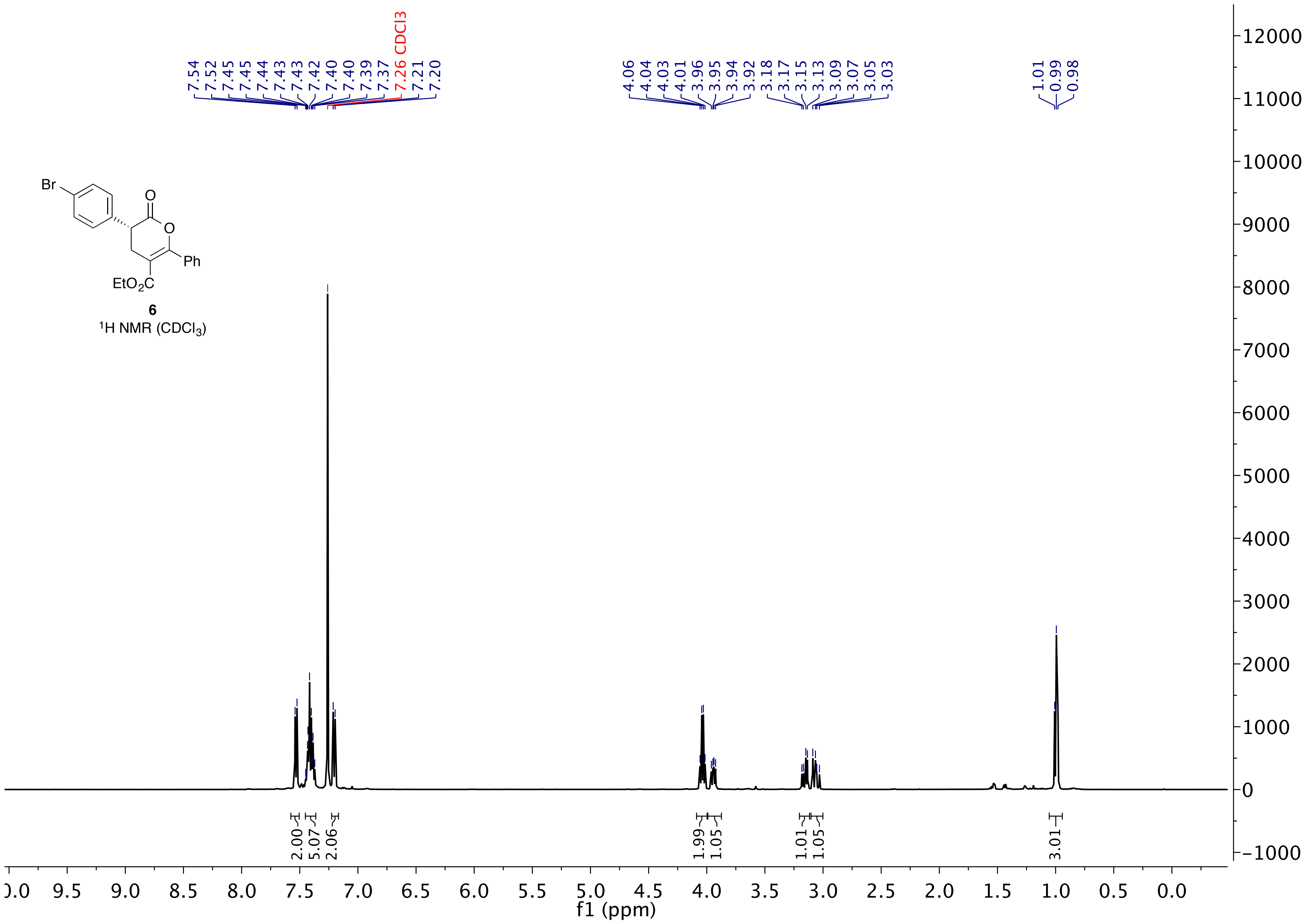

Br
O
O
Ph
EtO2C
6
1H NMR (CDCl3)
7.54
7.52
7.45
7.45
7.44
7.43
7.43
7.42
7.40
7.40
7.39
7.37
7.26 CDCl3
7.21
7.20
4.06
4.04
4.03
4.01
3.96
3.95
3.94
3.92
3.18
3.17
3.15
3.13
3.09
3.07
3.05
3.03
1.01
0.99
0.98
2.00
5.07
2.06
1.99
1.05
1.01
1.05
3.01
f1 (ppm)

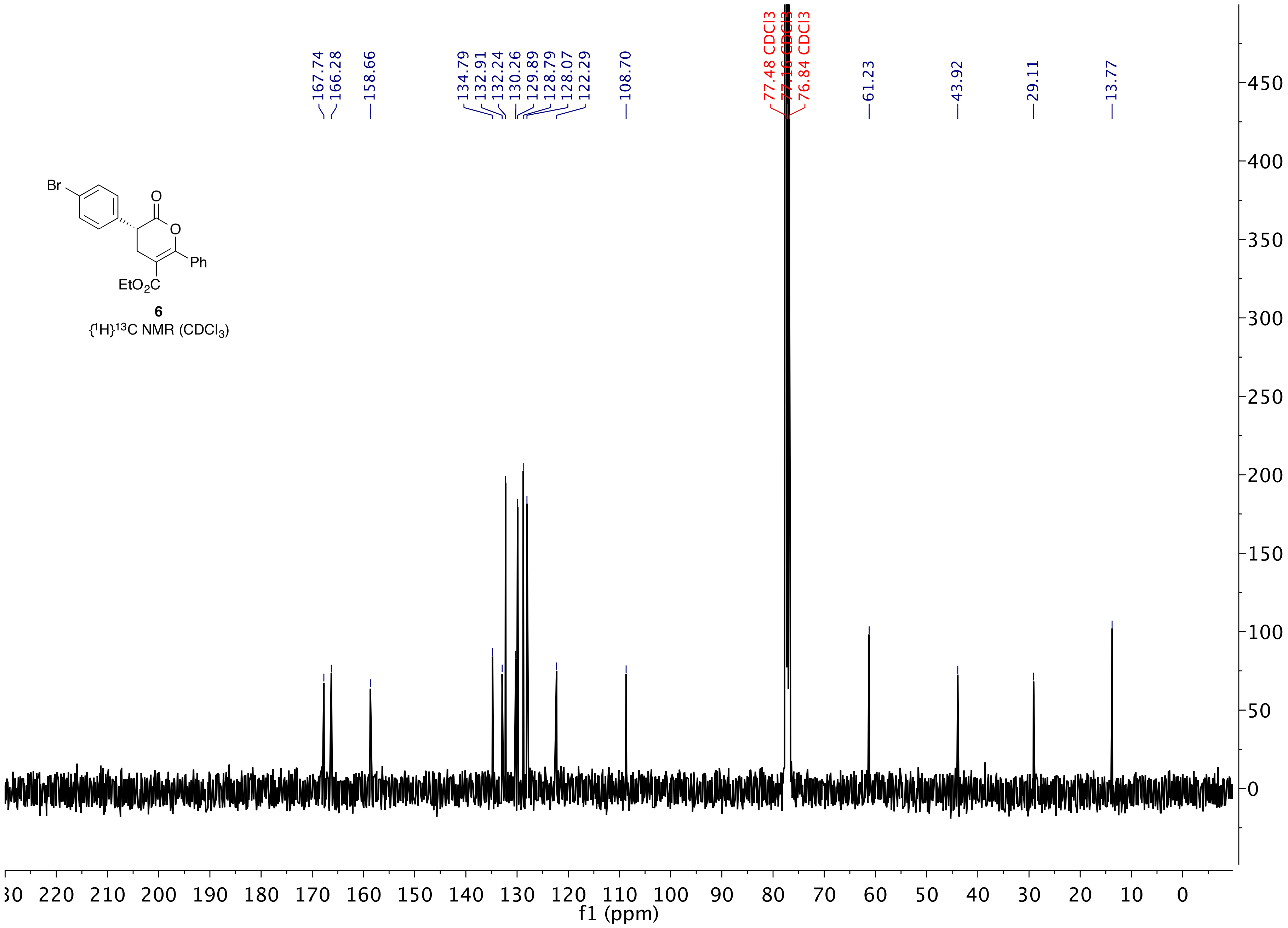
Br
O
O
Ph
EtO2C
6
{1H}13C NMR (CDCl3)
167.74
166.28
158.66
134.79
132.91
132.24
130.26
129.89
128.79
128.07
122.29
108.70
77.48 CDCl3
77.16 CDCl3
76.84 CDCl3
61.23
43.92
29.11
13.77
f1 (ppm)

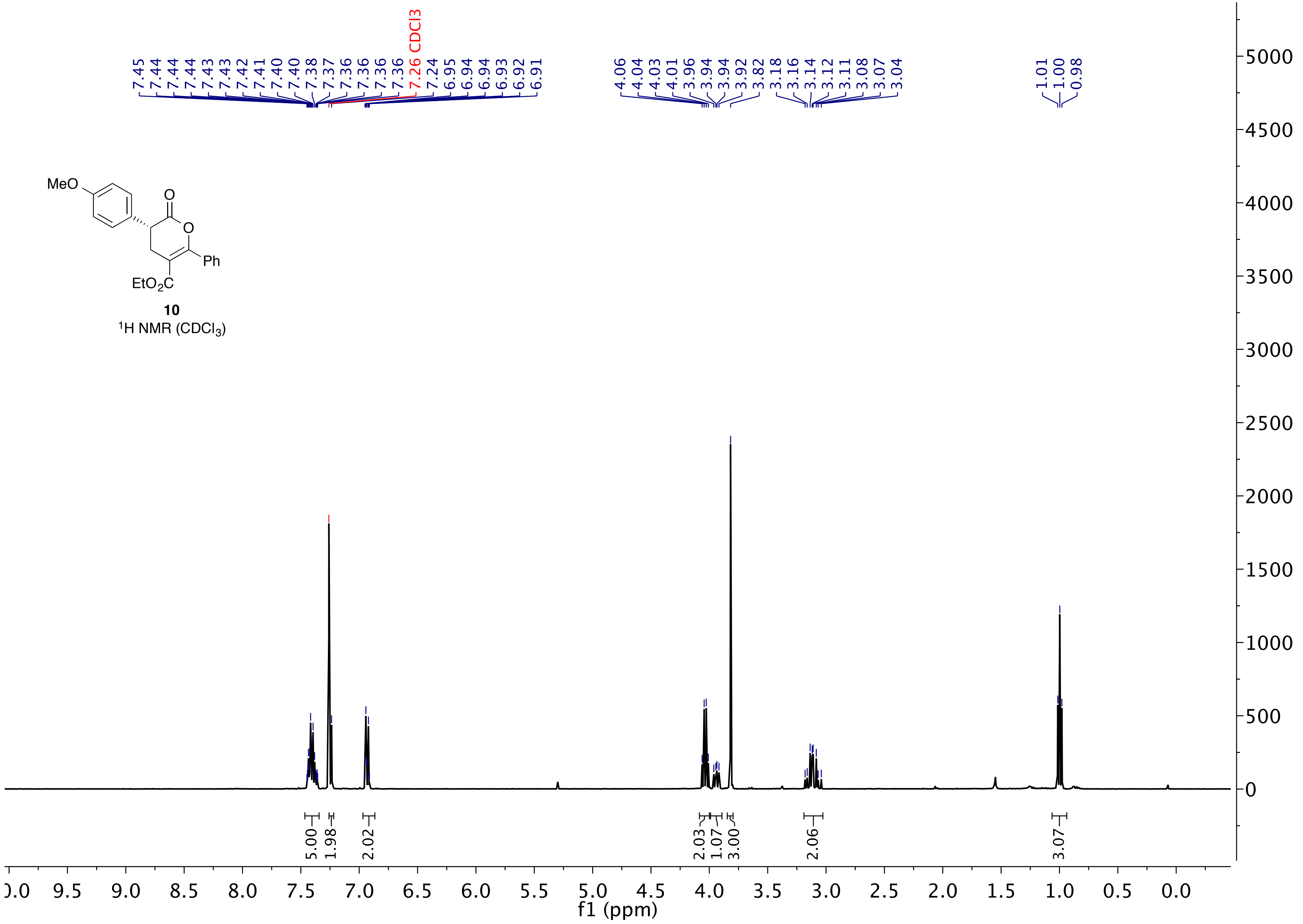
MeO
O
O
Ph
EtO2C
10
^{1}H NMR ($CDCl_3$)
7.45
7.44
7.44
7.44
7.43
7.43
7.42
7.41
7.40
7.40
7.38
7.37
7.36
7.36
7.36
7.36
7.26 CDCl3
7.24
6.95
6.94
6.94
6.93
6.92
6.91
4.06
4.04
4.03
4.01
3.96
3.94
3.94
3.92
3.82
3.18
3.16
3.14
3.12
3.11
3.08
3.07
3.04
1.01
1.00
0.98
5.00
1.98
2.02
2.03
1.07
3.00
2.06
3.07
f1 (ppm)

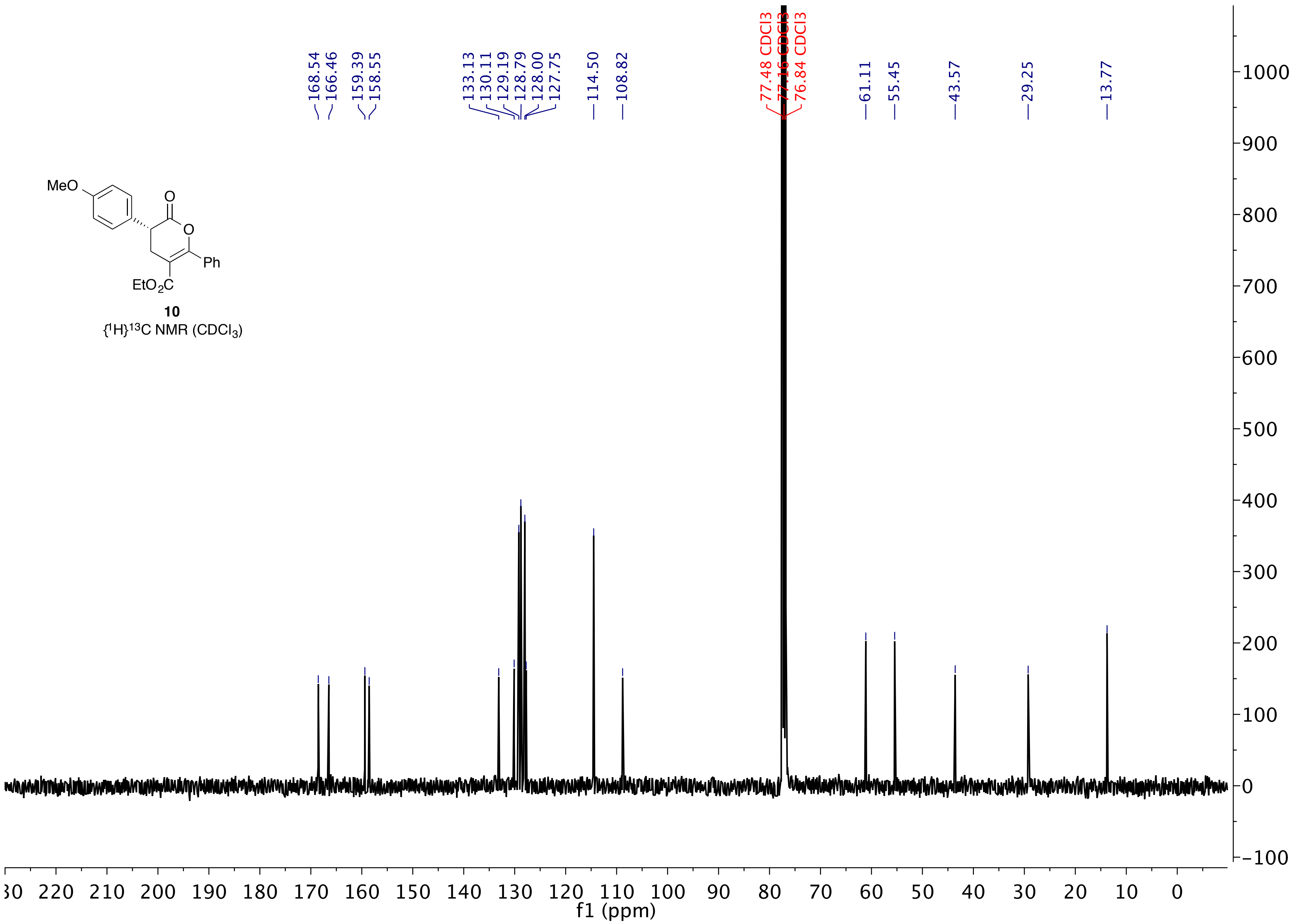
MeO
O
O
Ph
EtO2C
10
{1H}13C NMR (CDCl3)
168.54
166.46
159.39
158.55
133.13
130.11
129.19
128.79
128.00
127.75
114.50
108.82
77.48 CDCl3
77.16 CDCl3
76.84 CDCl3
61.11
55.45
43.57
29.25
13.77
f1 (ppm)

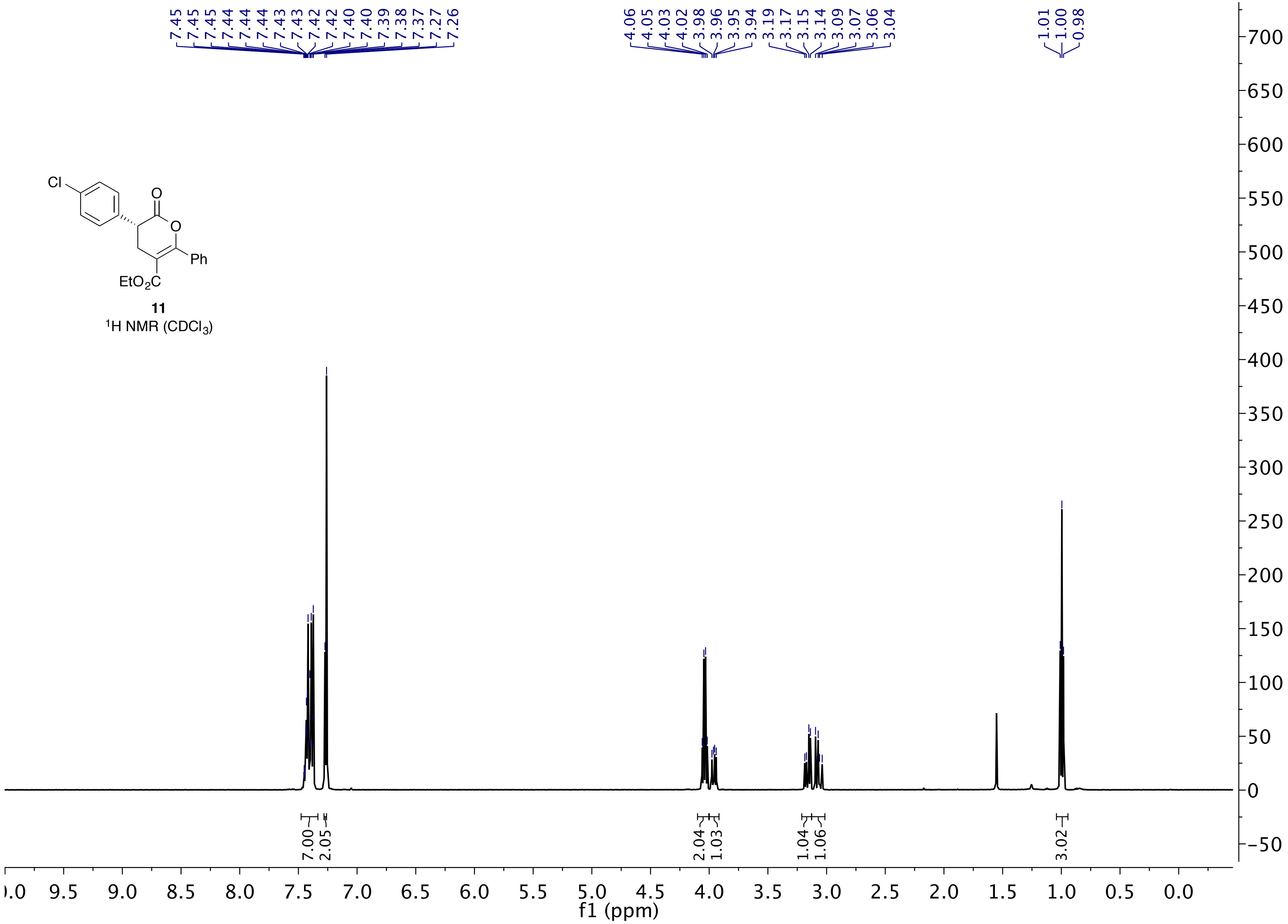

11
¹H NMR (CDCl3)
7.45 7.45 7.45 7.44 7.44 7.44 7.43 7.43 7.42 7.42 7.40 7.40 7.39 7.38 7.37 7.27 7.26
4.06 4.05 4.03 4.02 3.98 3.96 3.95 3.94 3.19 3.17 3.15 3.14 3.09 3.07 3.06 3.04
1.01 1.00 0.98
7.00 2.05 2.04 1.03 1.04 1.06 3.02
f1 (ppm)

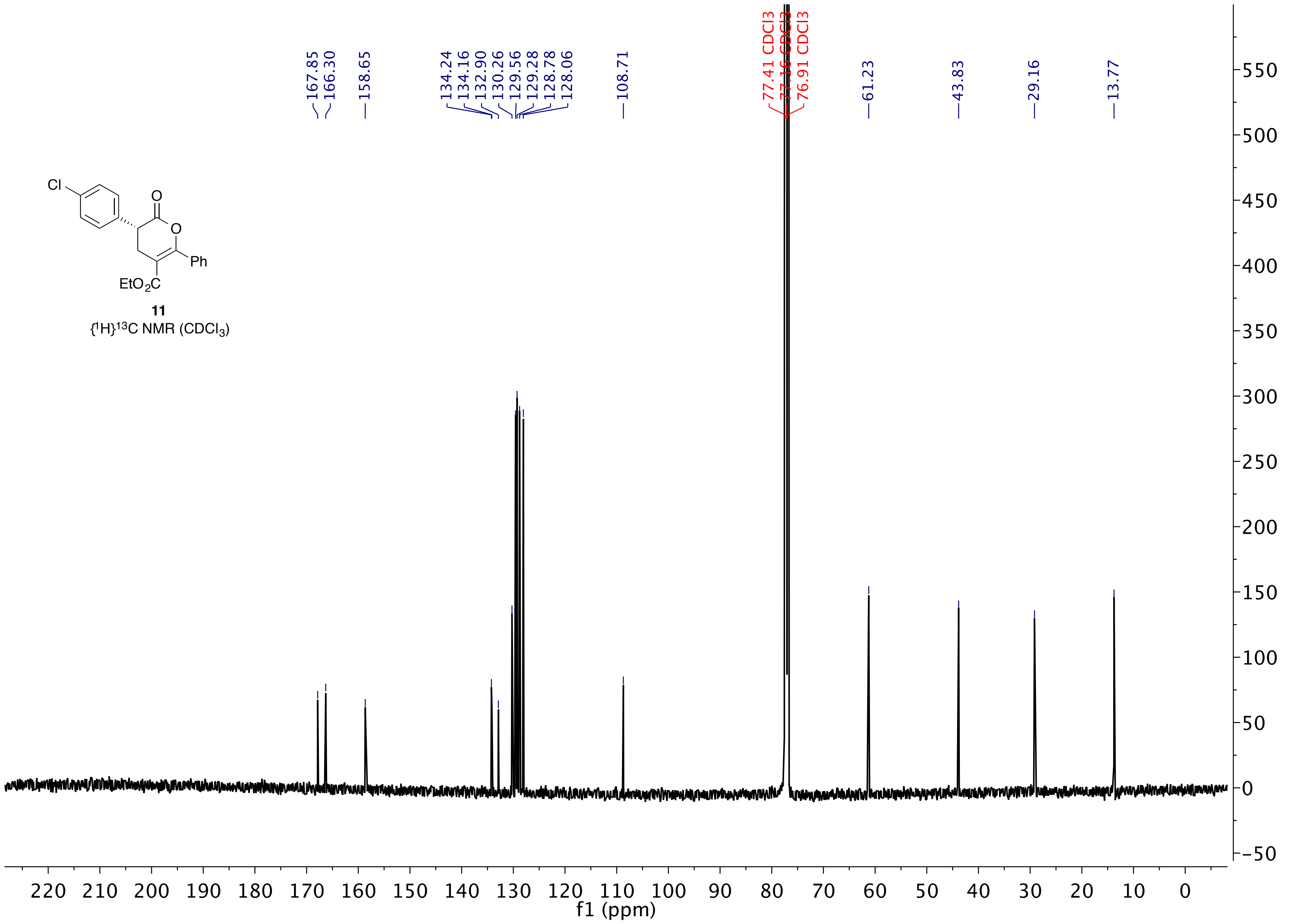
Cl
O
O
Ph
EtO2C
11
{1H}13C NMR (CDCl3)
167.85
166.30
158.65
134.24
134.16
132.90
130.26
129.56
129.28
128.78
128.06
108.71
77.41 CDCl3
77.16 CDCl3
76.91 CDCl3
61.23
43.83
29.16
13.77
f1 (ppm)

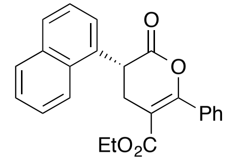

12
^{1}H NMR (CDCl$_3$)

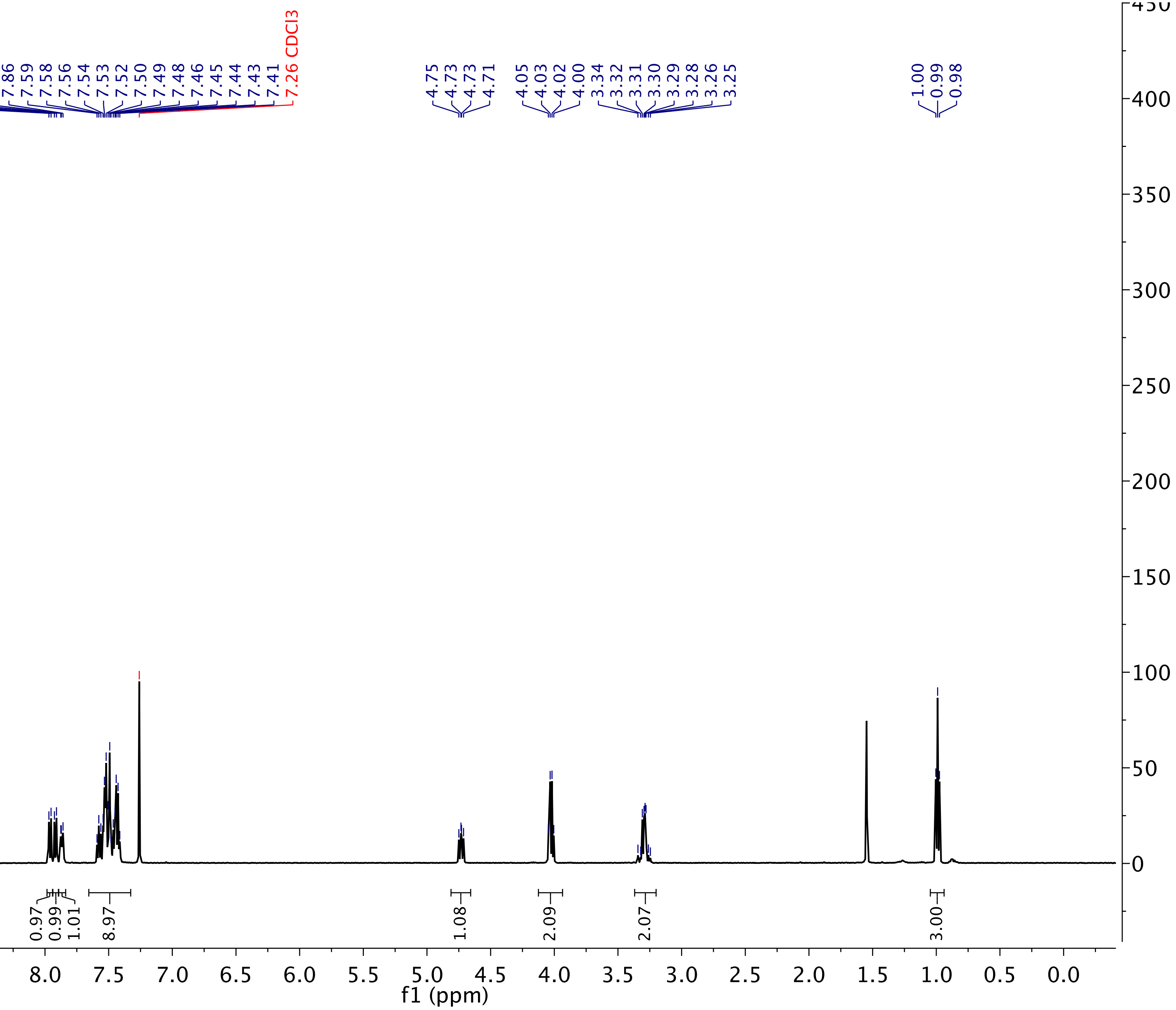

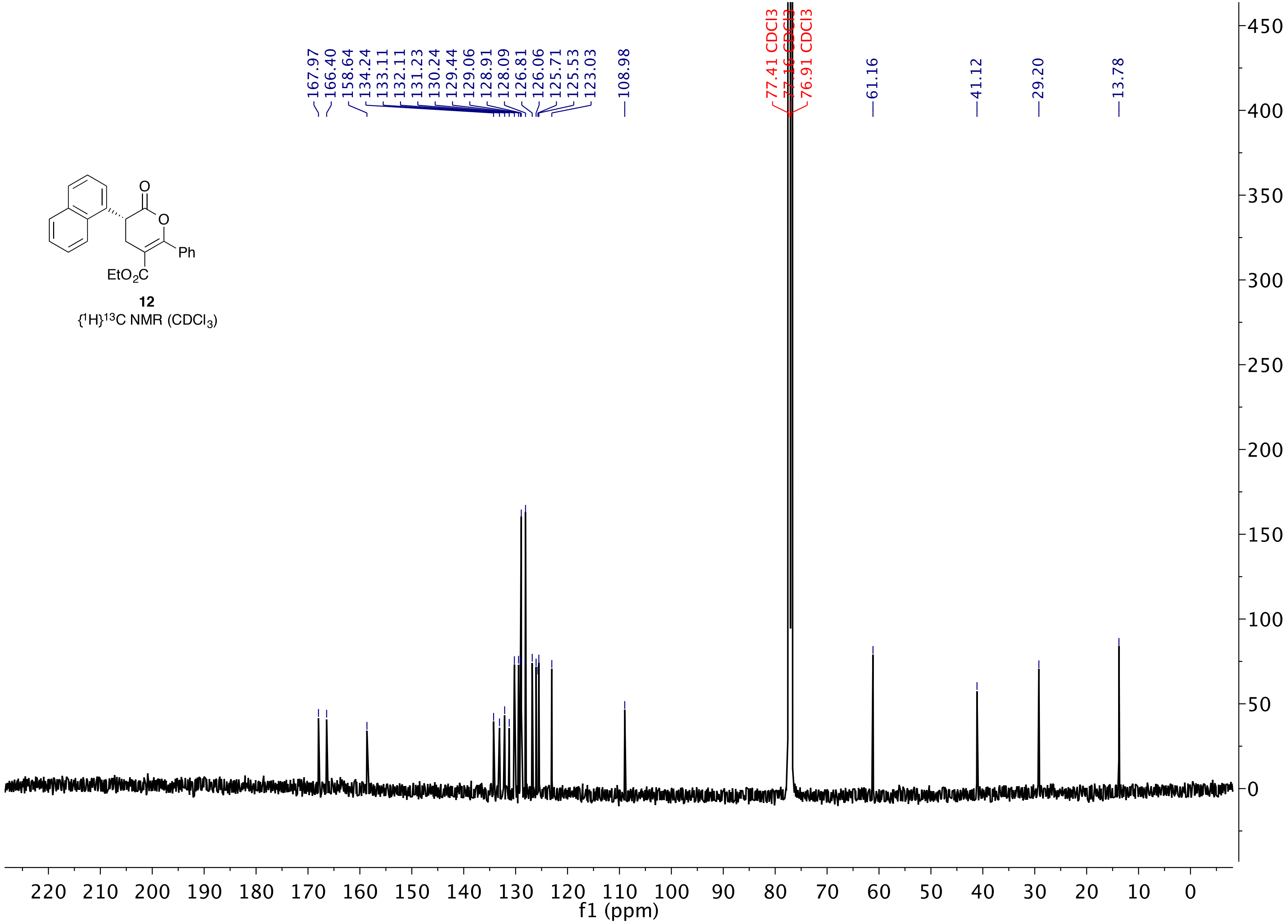

12

$\{^1H\}^{13}C$ NMR ($CDCl_3$)

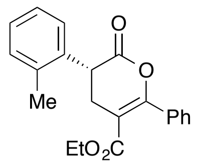

13

^{1}H NMR ($CDCl_3$)

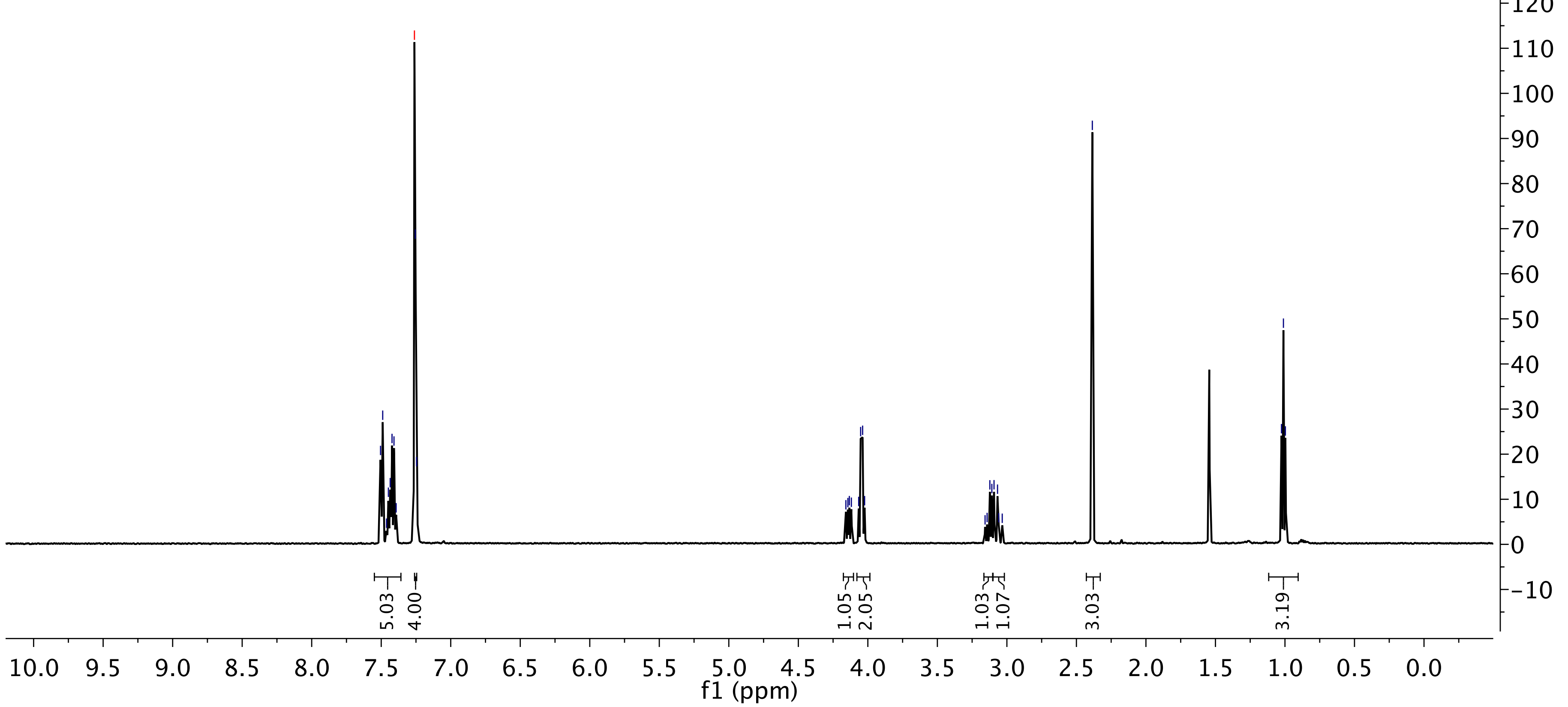

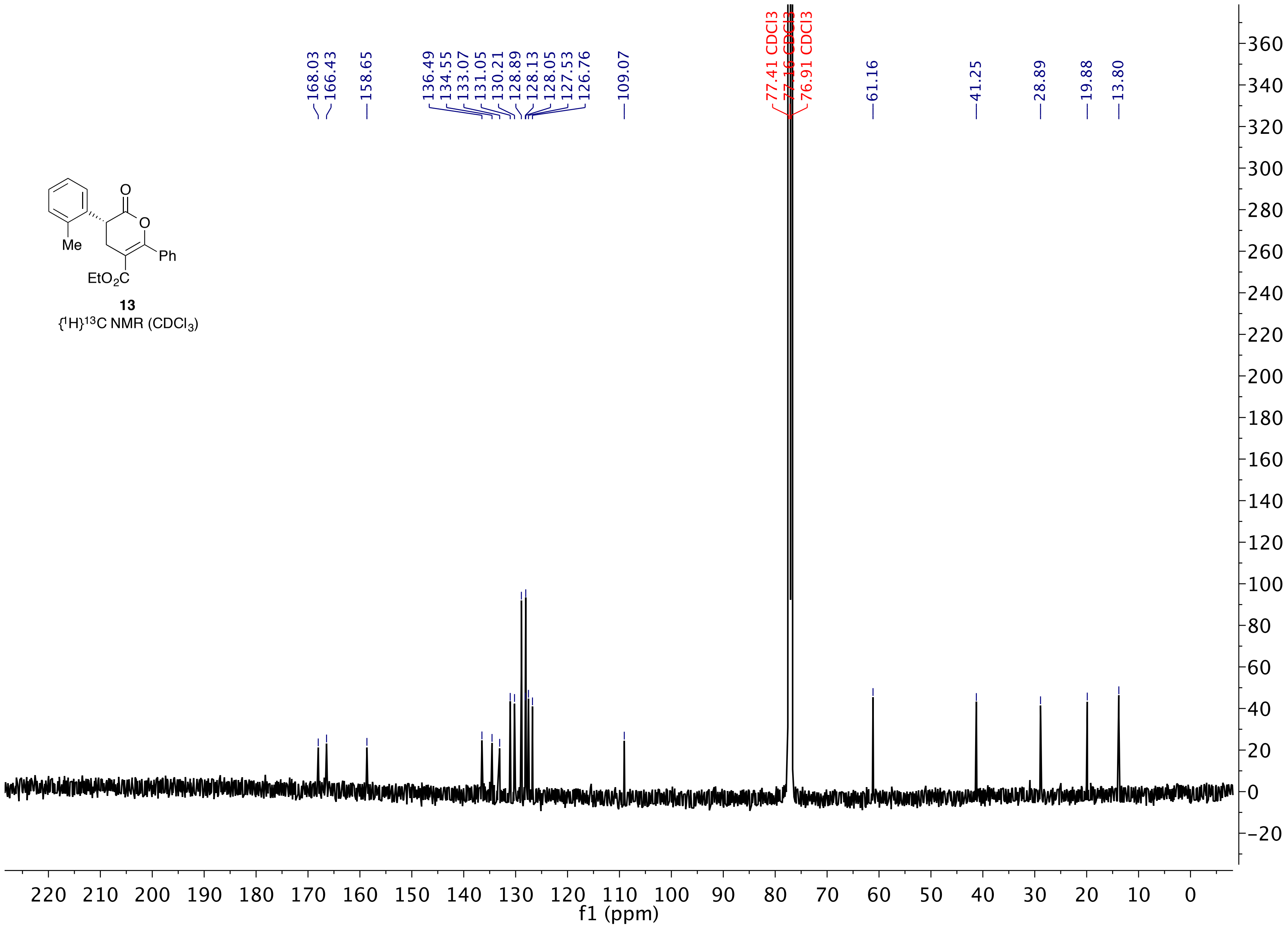

Me
O
O
Ph
EtO2C
13
{1H}13C NMR (CDCl3)
168.03
166.43
158.65
136.49
134.55
133.07
131.05
130.21
128.89
128.13
128.05
127.53
126.76
109.07
77.41 CDCl3
77.16 CDCl3
76.91 CDCl3
61.16
41.25
28.89
19.88
13.80
f1 (ppm)

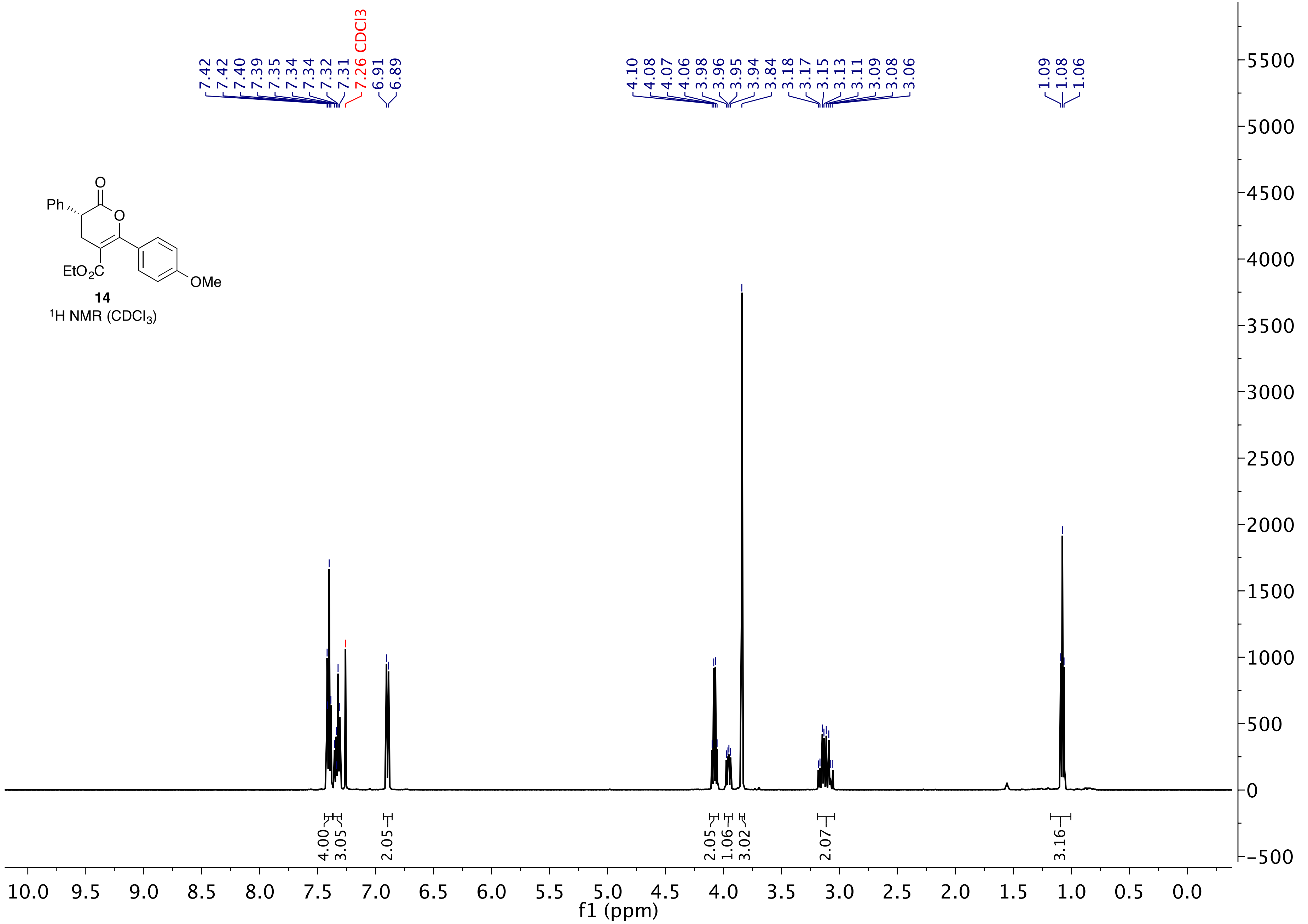
Ph
O
O
EtO2C
OMe
14
1H NMR (CDCl3)
7.42 7.42 7.40 7.39 7.35 7.34 7.34 7.32 7.31
7.26 CDCl3
6.91 6.89
4.10 4.08 4.07 4.06 3.98 3.96 3.95 3.94 3.84
3.18 3.17 3.15 3.13 3.11 3.09 3.08 3.06
1.09 1.08 1.06
4.00 3.05 2.05 2.05 1.06 3.02 2.07 3.16
f1 (ppm)

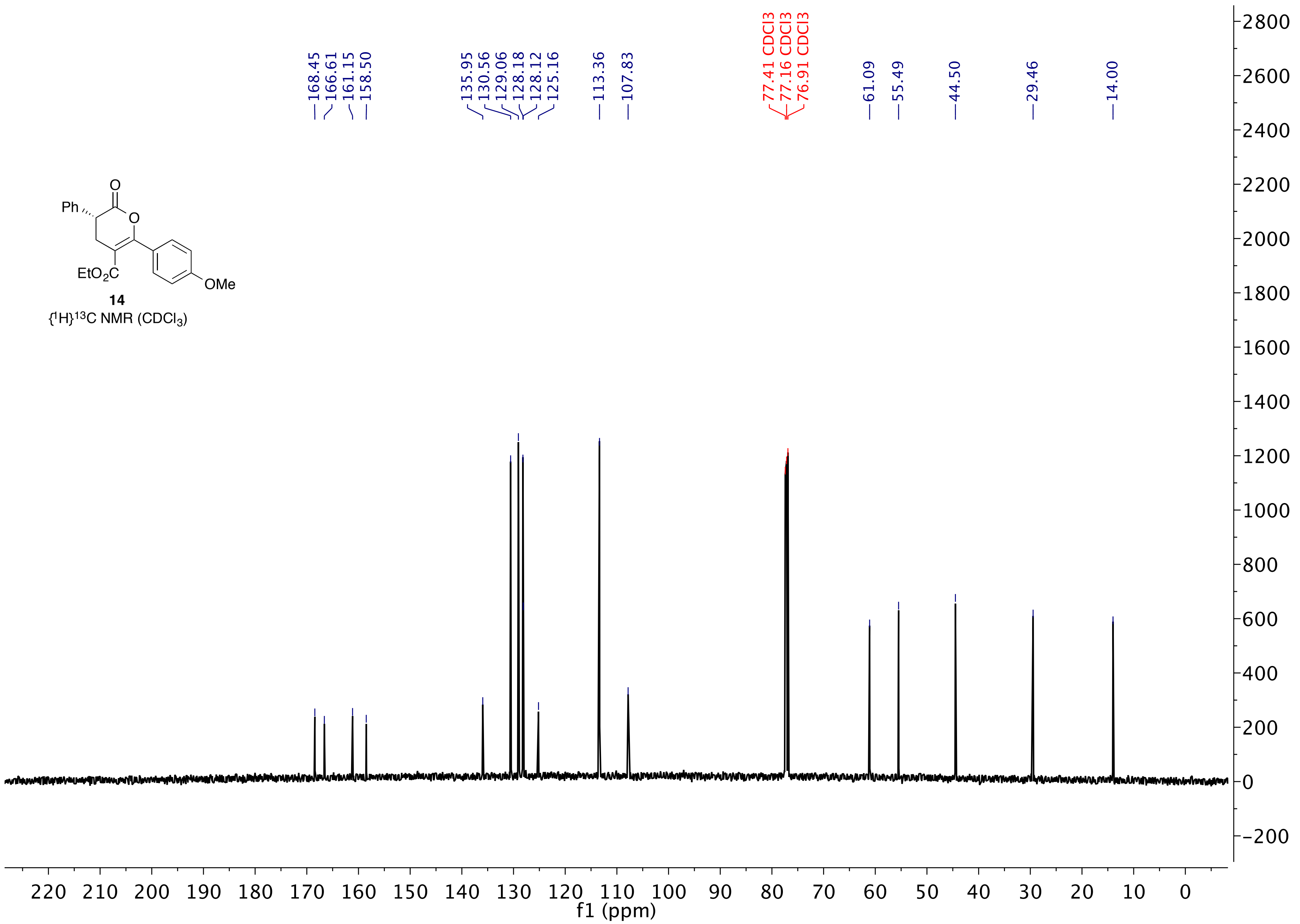

Ph
O
O
EtO2C
OMe
14
{1H}13C NMR (CDCl3)
168.45
166.61
161.15
158.50
135.95
130.56
129.06
128.18
128.12
125.16
113.36
107.83
77.41 CDCl3
77.16 CDCl3
76.91 CDCl3
61.09
55.49
44.50
29.46
14.00
f1 (ppm)

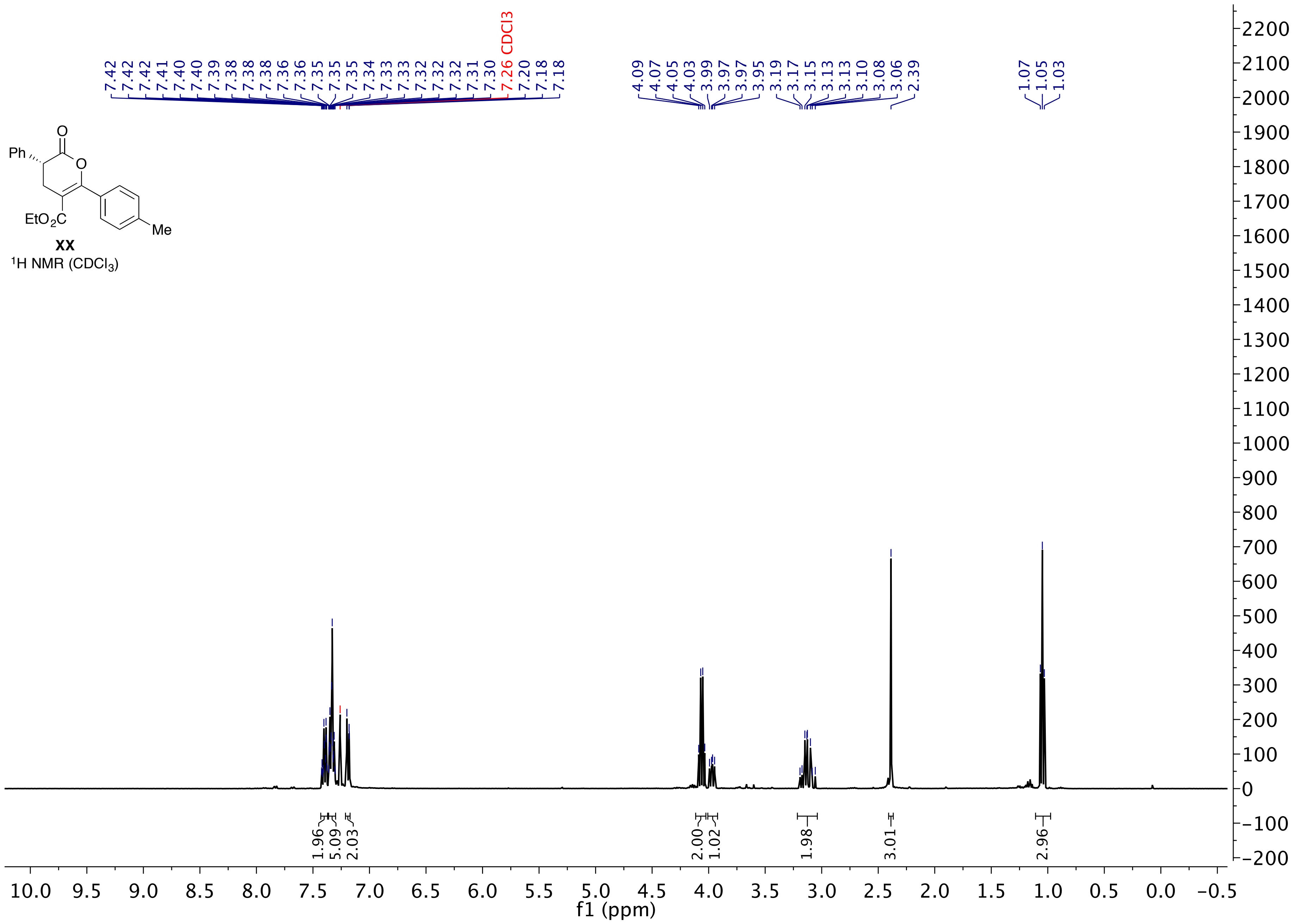

Ph
O
O
EtO2C
Me
XX
1H NMR (CDCl3)
7.42 7.42 7.42 7.41 7.40 7.40 7.39 7.38 7.38 7.38 7.36 7.36 7.35 7.35 7.35 7.34 7.33 7.33 7.32 7.32 7.32 7.31 7.30 7.26 CDCl3 7.20 7.18 7.18
4.09 4.07 4.05 4.03 3.99 3.97 3.97 3.95 3.19 3.17 3.15 3.13 3.13 3.10 3.08 3.06 2.39
1.07 1.05 1.03
1.96 5.09 2.03 2.00 1.02 1.98 3.01 2.96
f1 (ppm)

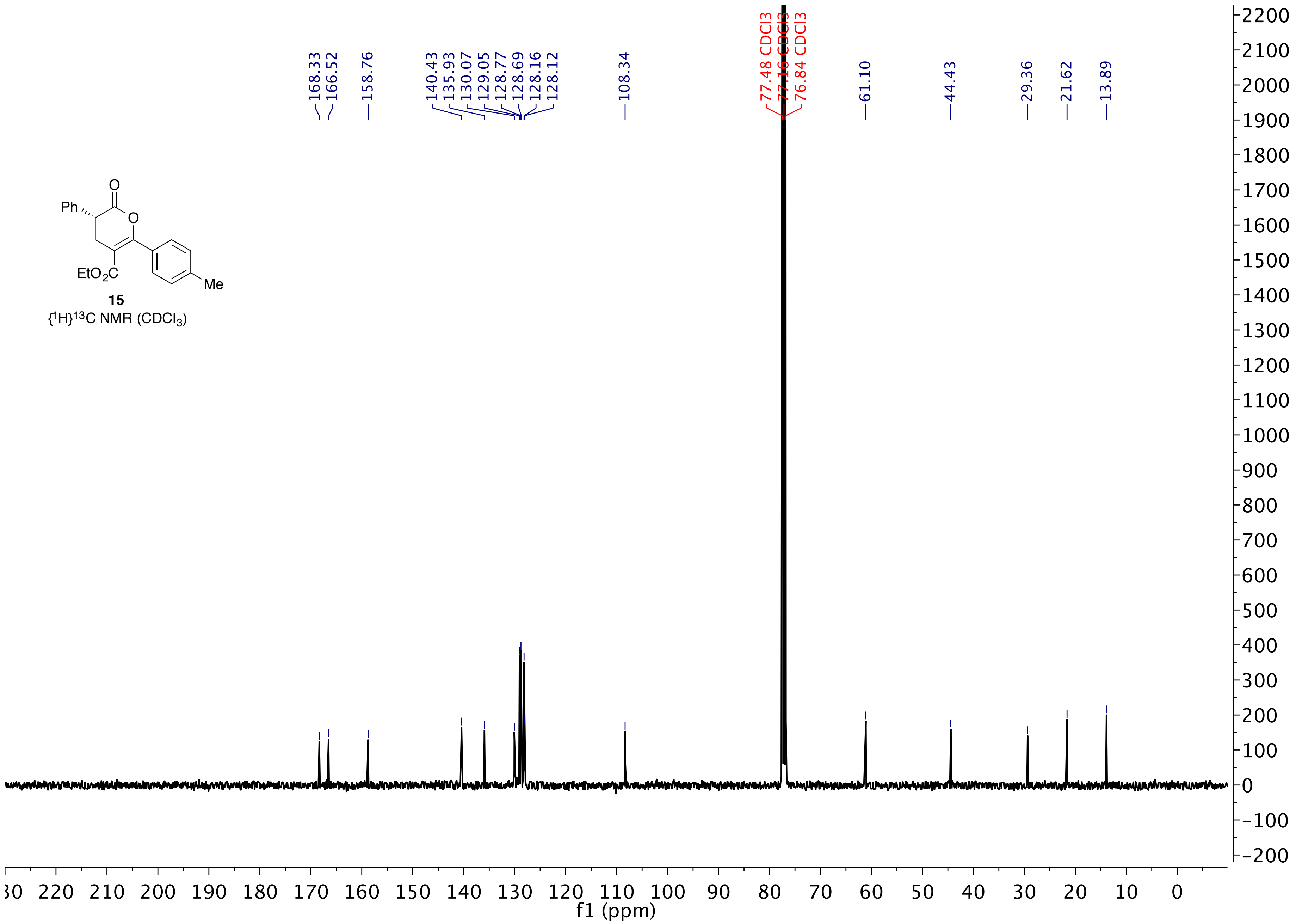
168.33
166.52
158.76
140.43
135.93
130.07
129.05
128.77
128.69
128.16
128.12
108.34
77.48 CDCl3
77.16 CDCl3
76.84 CDCl3
61.10
44.43
29.36
21.62
13.89
Ph
O
O
EtO2C
Me
15
{1H}13C NMR (CDCl3)
f1 (ppm)

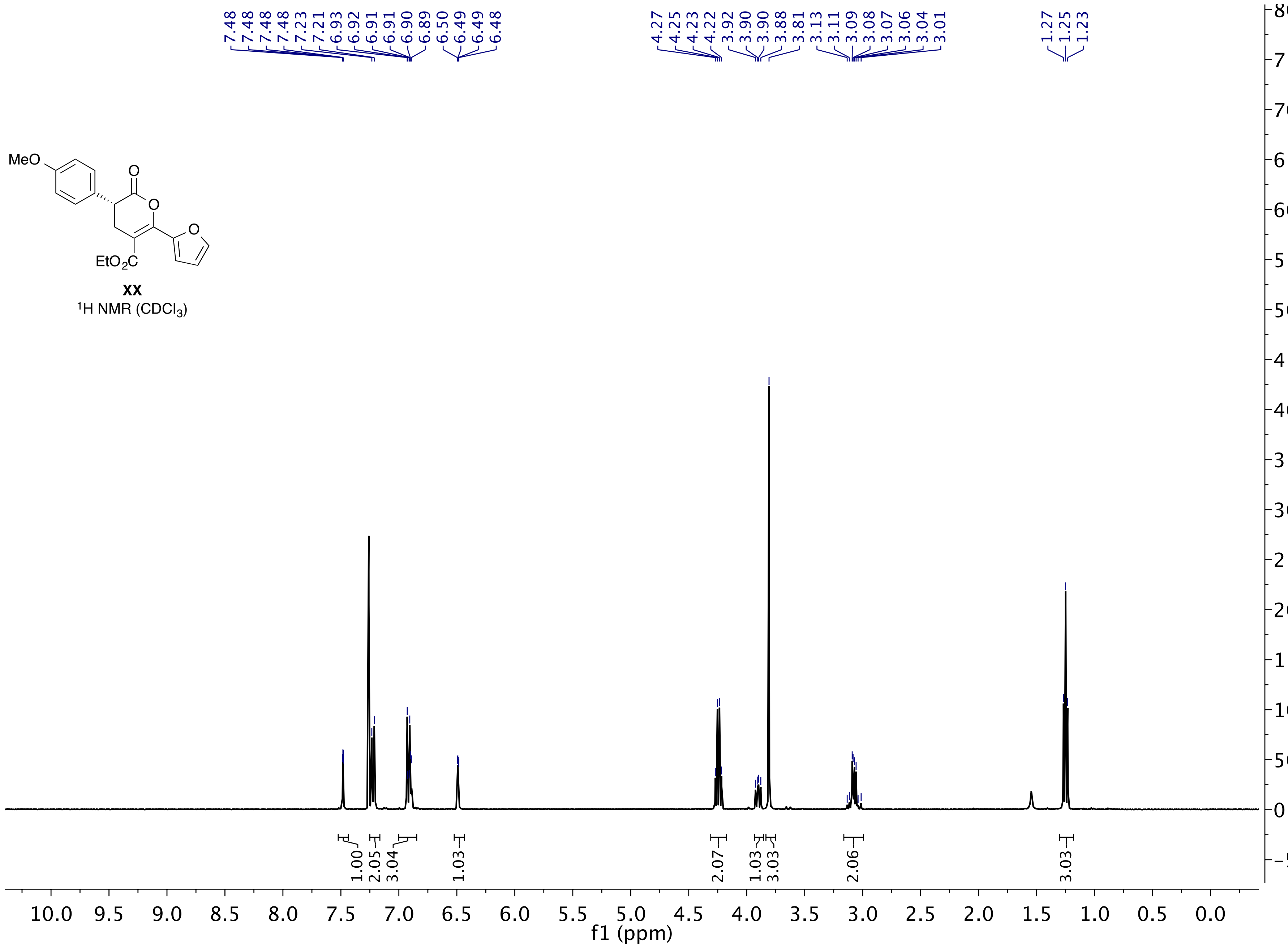

MeO
O
O
O
EtO2C
XX
1H NMR (CDCl3)
7.48
7.48
7.48
7.48
7.23
7.21
6.93
6.92
6.91
6.91
6.90
6.89
6.50
6.49
6.49
6.48
4.27
4.25
4.23
4.22
3.92
3.90
3.90
3.88
3.81
3.13
3.11
3.09
3.08
3.07
3.06
3.04
3.01
1.27
1.25
1.23
1.00
2.05
3.04
1.03
2.07
1.03
3.03
2.06
3.03
10.0 9.5 9.0 8.5 8.0 7.5 7.0 6.5 6.0 5.5 5.0 4.5 4.0 3.5 3.0 2.5 2.0 1.5 1.0 0.5 0.0
f1 (ppm)
800 750 700 650 600 550 500 450 400 350 300 250 200 150 100 50 0 −50

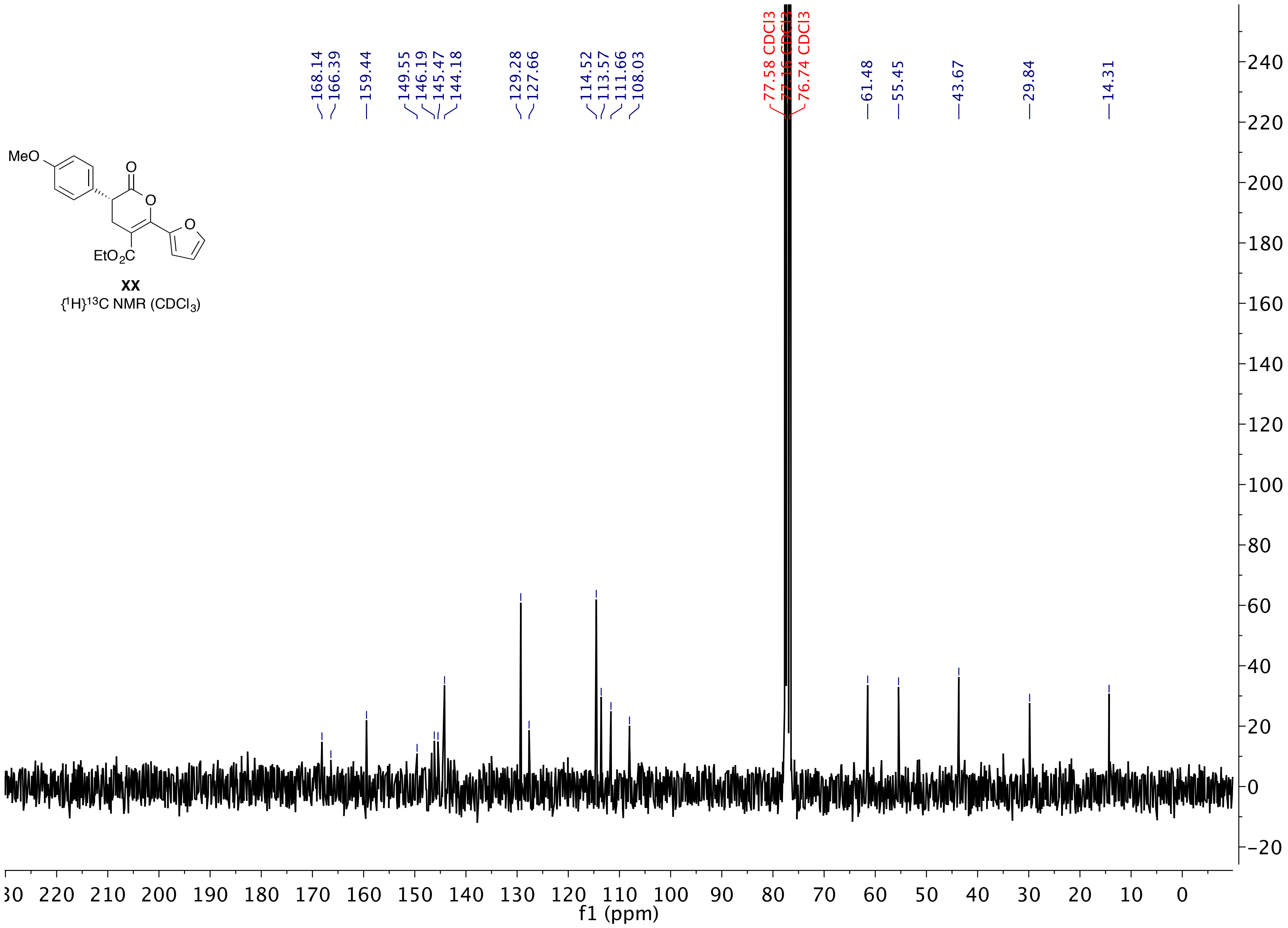
MeO
O
O
O
EtO2C
XX
{1H}13C NMR (CDCl3)
168.14
166.39
159.44
149.55
146.19
145.47
144.18
129.28
127.66
114.52
113.57
111.66
108.03
77.58 CDCl3
77.16 CDCl3
76.74 CDCl3
61.48
55.45
43.67
29.84
14.31
f1 (ppm)

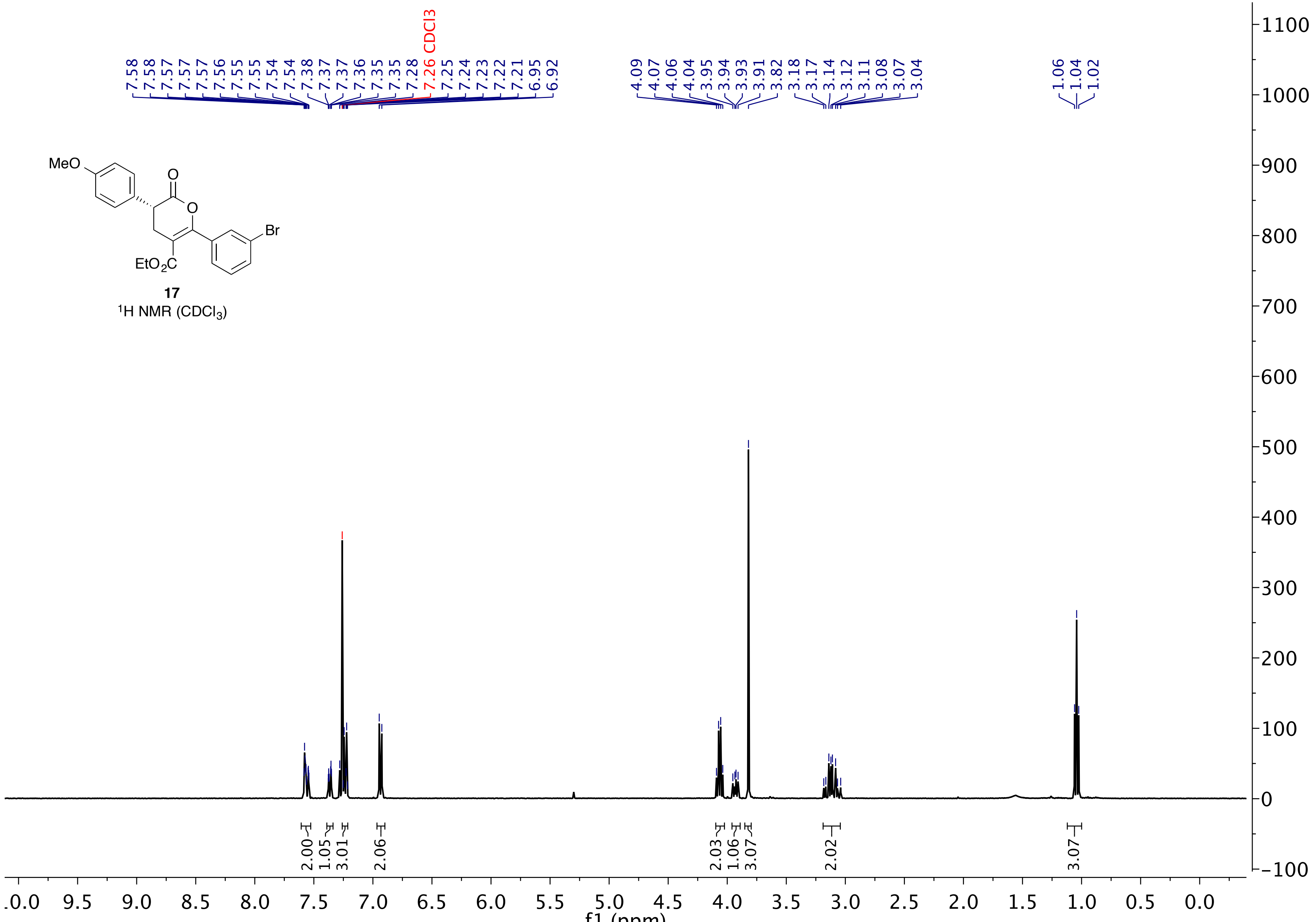

17
^1H NMR ($CDCl_3$)

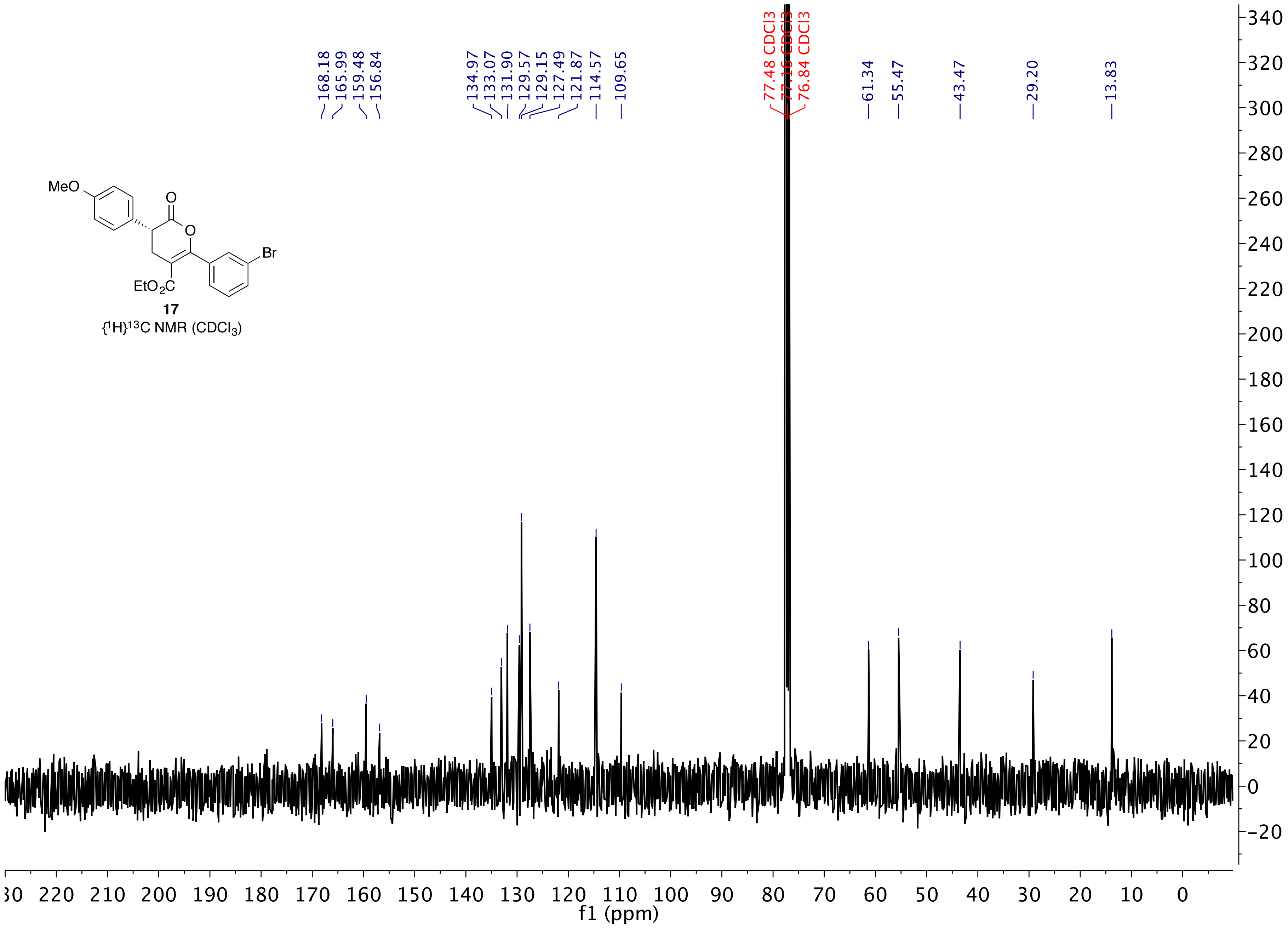
168.18
165.99
159.48
156.84
134.97
133.07
131.90
129.57
129.15
127.49
121.87
114.57
109.65
77.48 CDCl3
77.16 CDCl3
76.84 CDCl3
61.34
55.47
43.47
29.20
13.83
MeO
O
O
Br
EtO2C
17
{1H}13C NMR (CDCl3)
f1 (ppm)

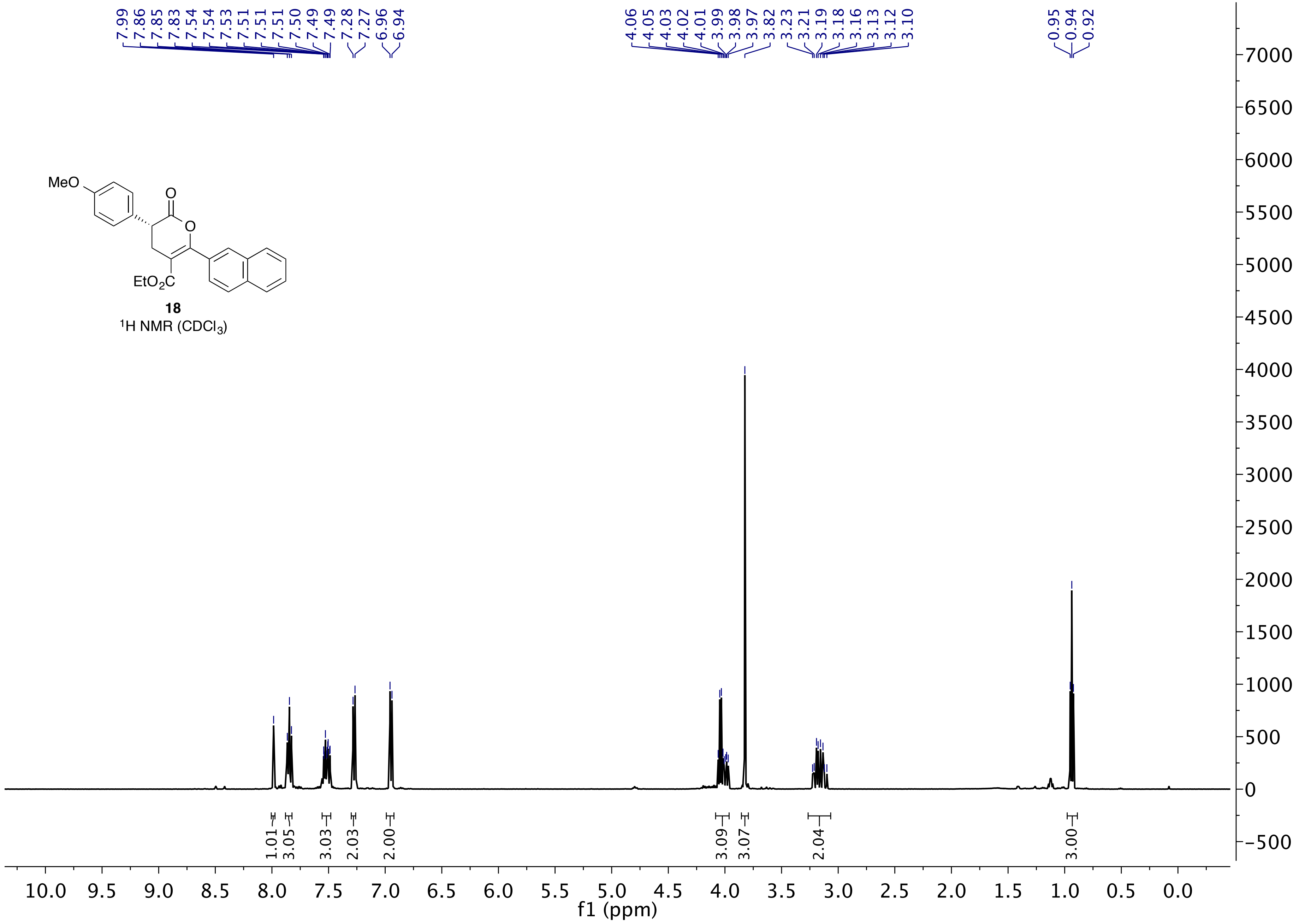
MeO
O
O
EtO2C
18
1H NMR (CDCl3)
7.99
7.86
7.85
7.83
7.54
7.54
7.53
7.51
7.51
7.51
7.50
7.49
7.49
7.28
7.27
6.96
6.94
4.06
4.05
4.03
4.02
4.01
3.99
3.98
3.97
3.82
3.23
3.21
3.19
3.18
3.16
3.13
3.12
3.10
0.95
0.94
0.92
1.01
3.05
3.03
2.03
2.00
3.09
3.07
2.04
3.00
10.0
9.5
9.0
8.5
8.0
7.5
7.0
6.5
6.0
5.5
5.0
4.5
4.0
3.5
3.0
2.5
2.0
1.5
1.0
0.5
0.0
f1 (ppm)
7000
6500
6000
5500
5000
4500
4000
3500
3000
2500
2000
1500
1000
500
0
-500

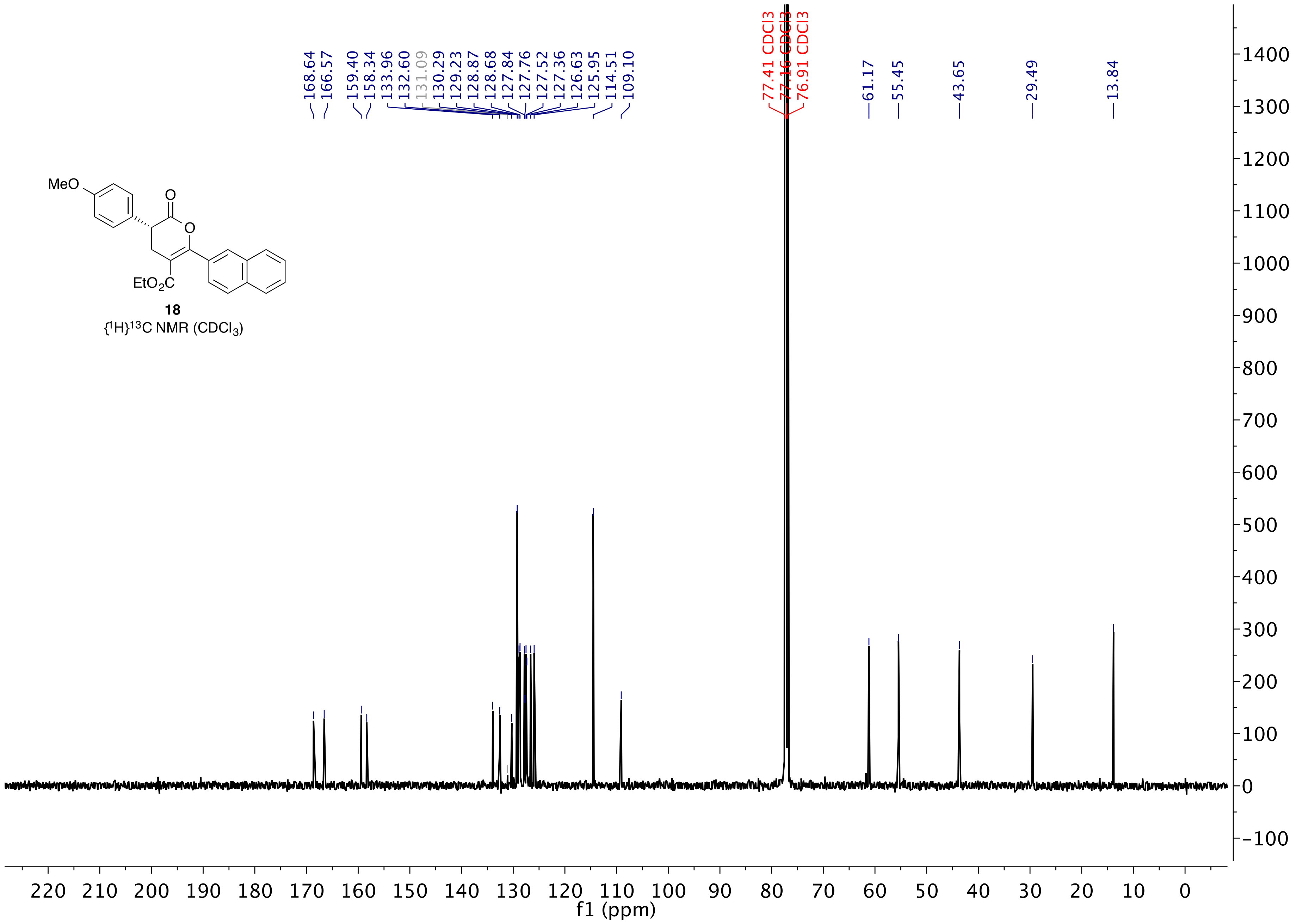

18

{^{1}H}^{13}C NMR ($CDCl_3$)

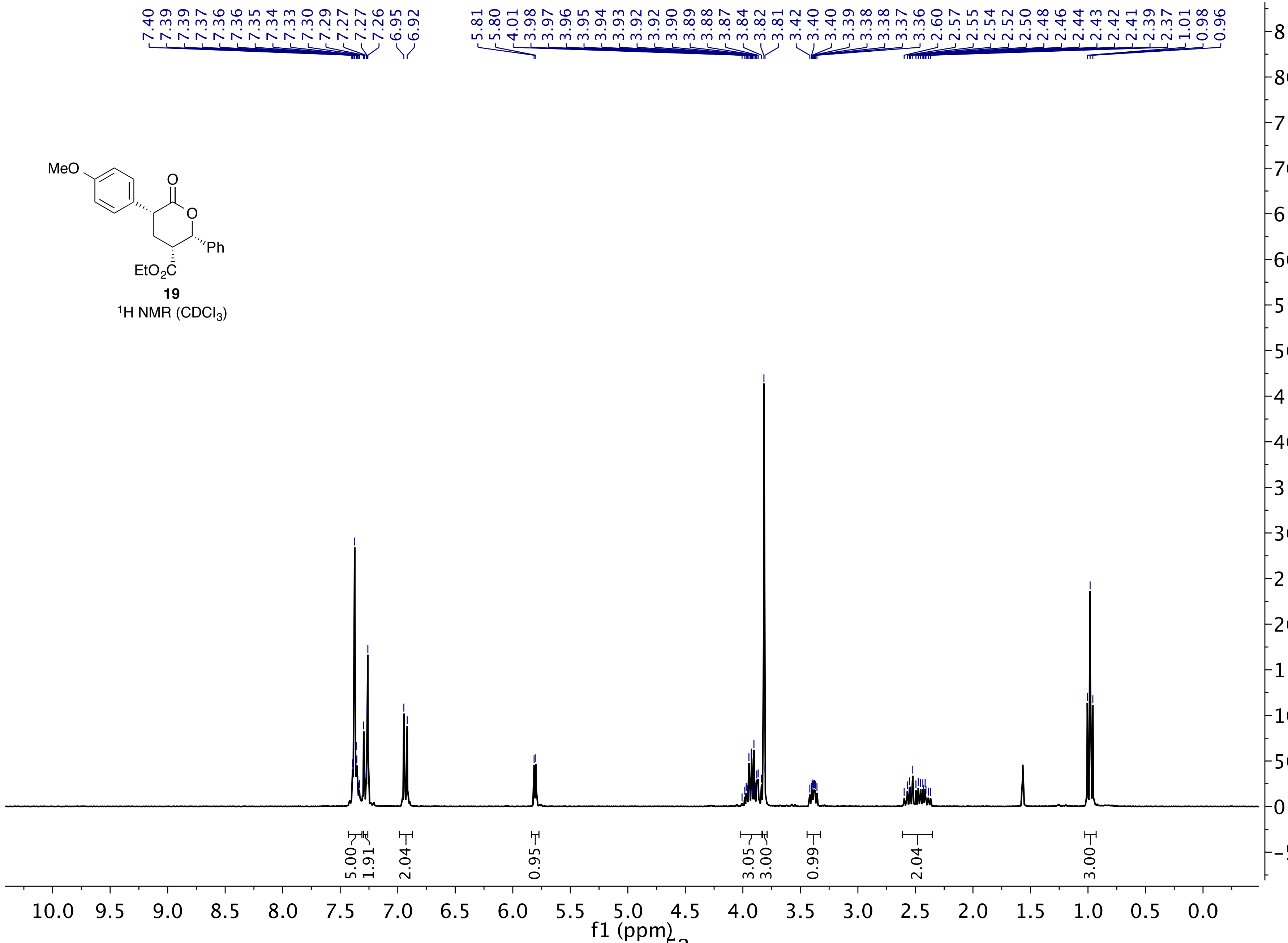

MeO
O
O
Ph
EtO2C
19
1H NMR (CDCl3)
7.40 7.39 7.39 7.37 7.36 7.36 7.35 7.34 7.33 7.30 7.29 7.27 7.27 7.26 6.95 6.92
5.81 5.80 4.01 3.98 3.97 3.96 3.95 3.94 3.93 3.92 3.92 3.90 3.89 3.88 3.87 3.84 3.82 3.81 3.42 3.40 3.40 3.39 3.38 3.38 3.37 3.36 2.60 2.57 2.55 2.54 2.52 2.50 2.48 2.46 2.44 2.43 2.42 2.41 2.39 2.37 1.01 0.98 0.96
5.00 1.91 2.04 0.95 3.05 3.00 0.99 2.04 3.00
f1 (ppm)

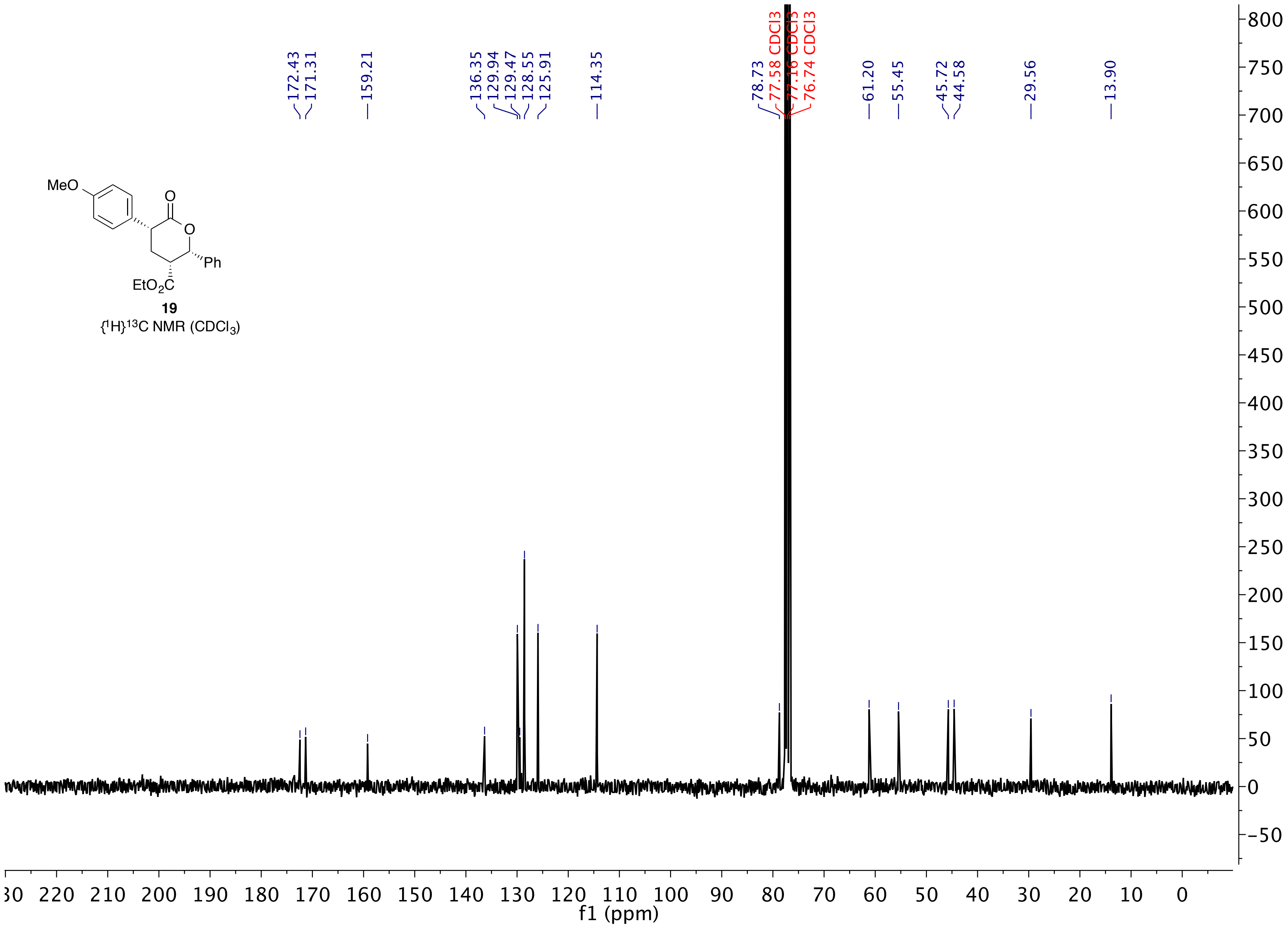

MeO
O
O
Ph
EtO2C
19
{1H}13C NMR (CDCl3)
172.43
171.31
159.21
136.35
129.94
129.47
128.55
125.91
114.35
78.73
77.58 CDCl3
77.16 CDCl3
76.74 CDCl3
61.20
55.45
45.72
44.58
29.56
13.90
f1 (ppm)

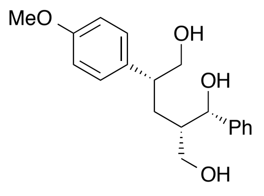

20
^{1}H NMR ($CDCl_3$)

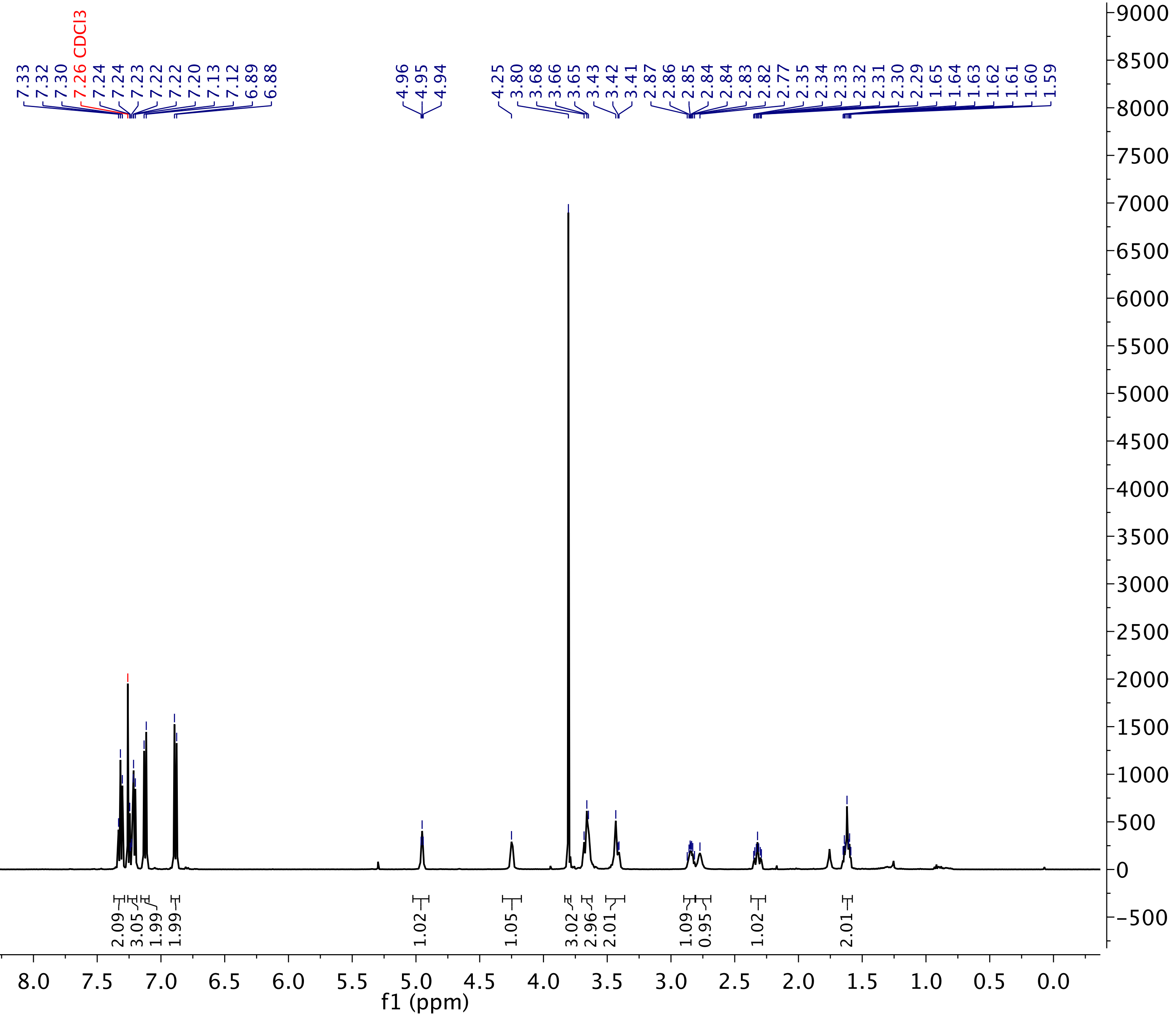

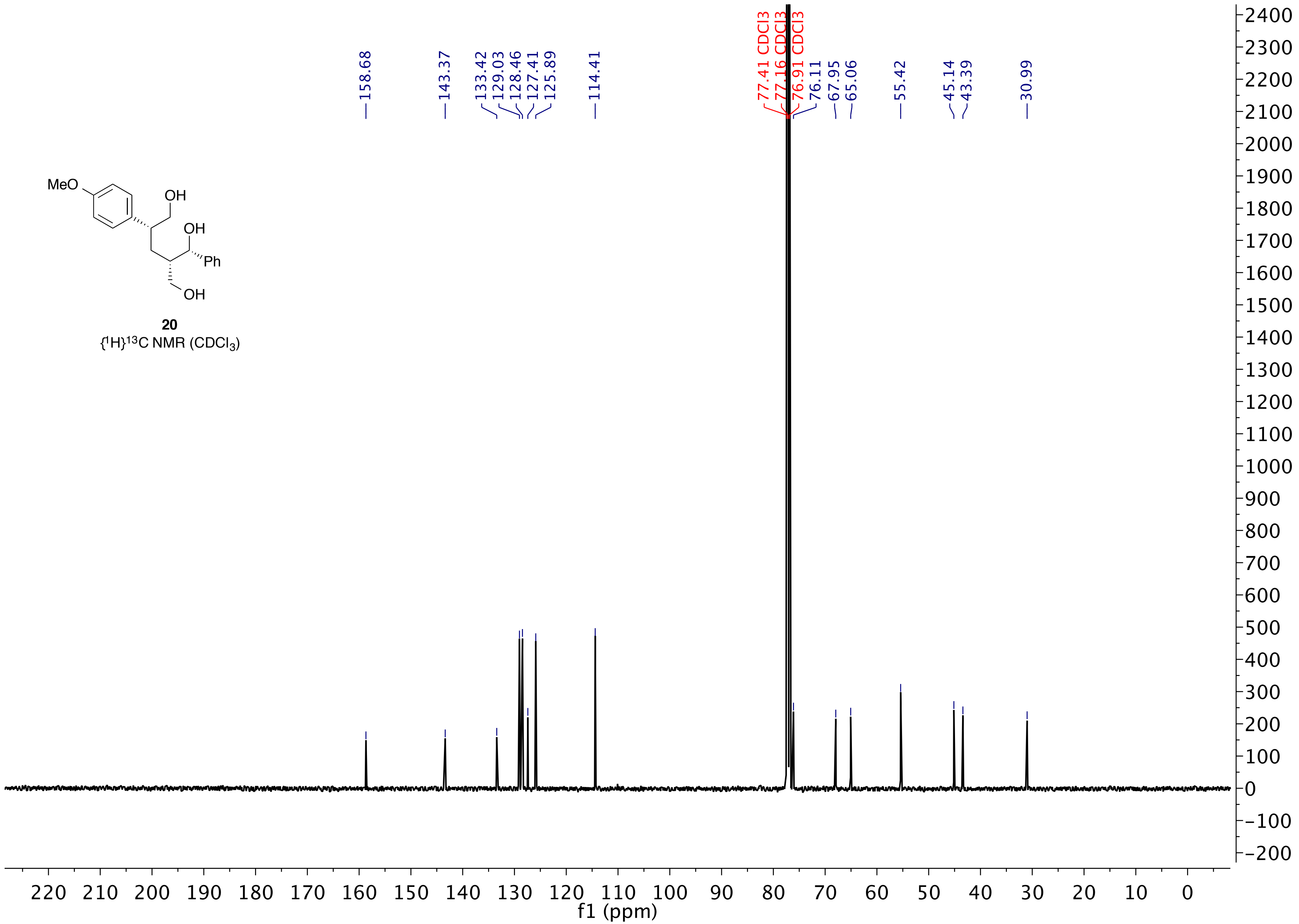

20
{^{1}H}^{13}C NMR (CDCl$_3$)

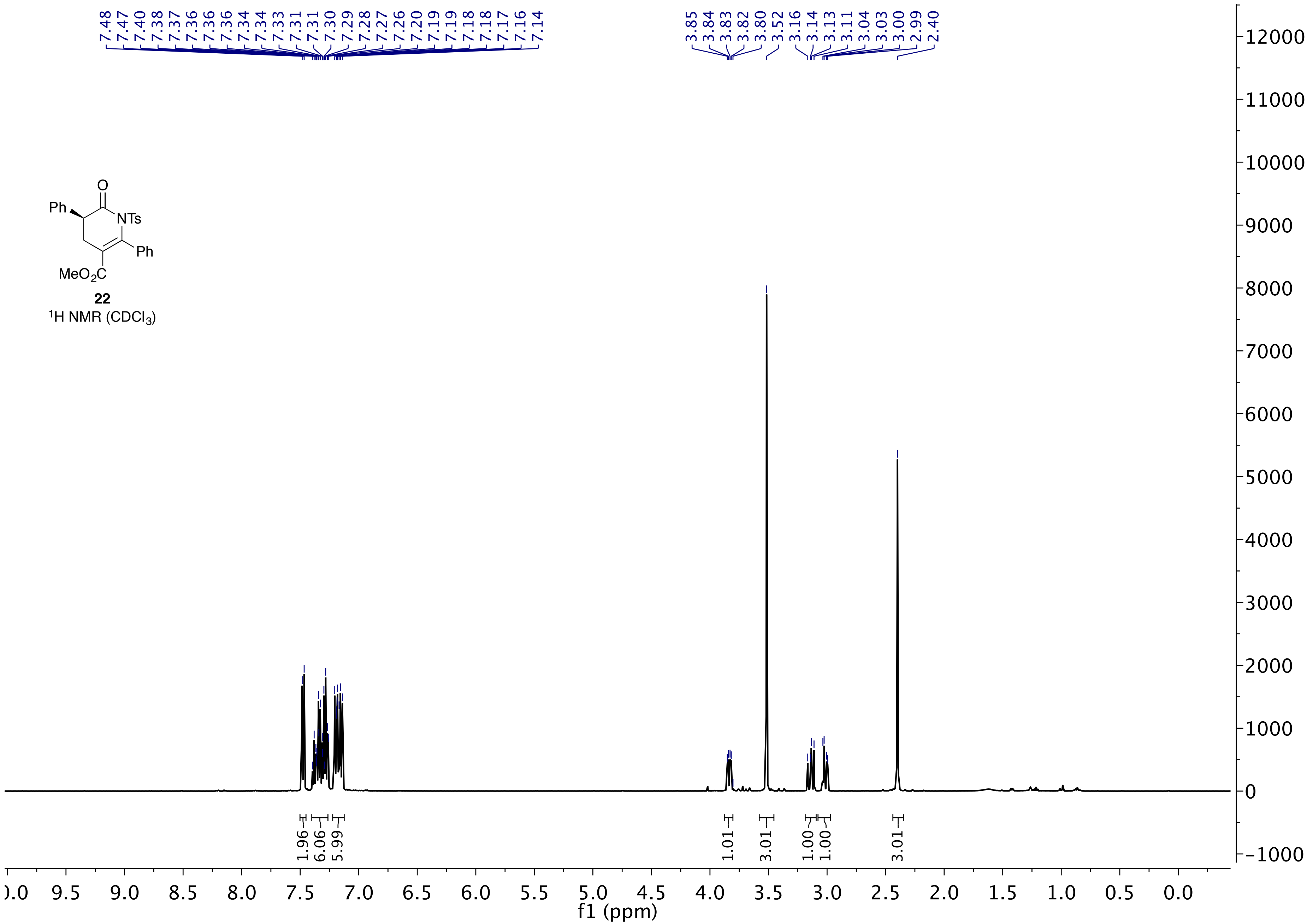

22

^{1}H NMR ($CDCl_3$)

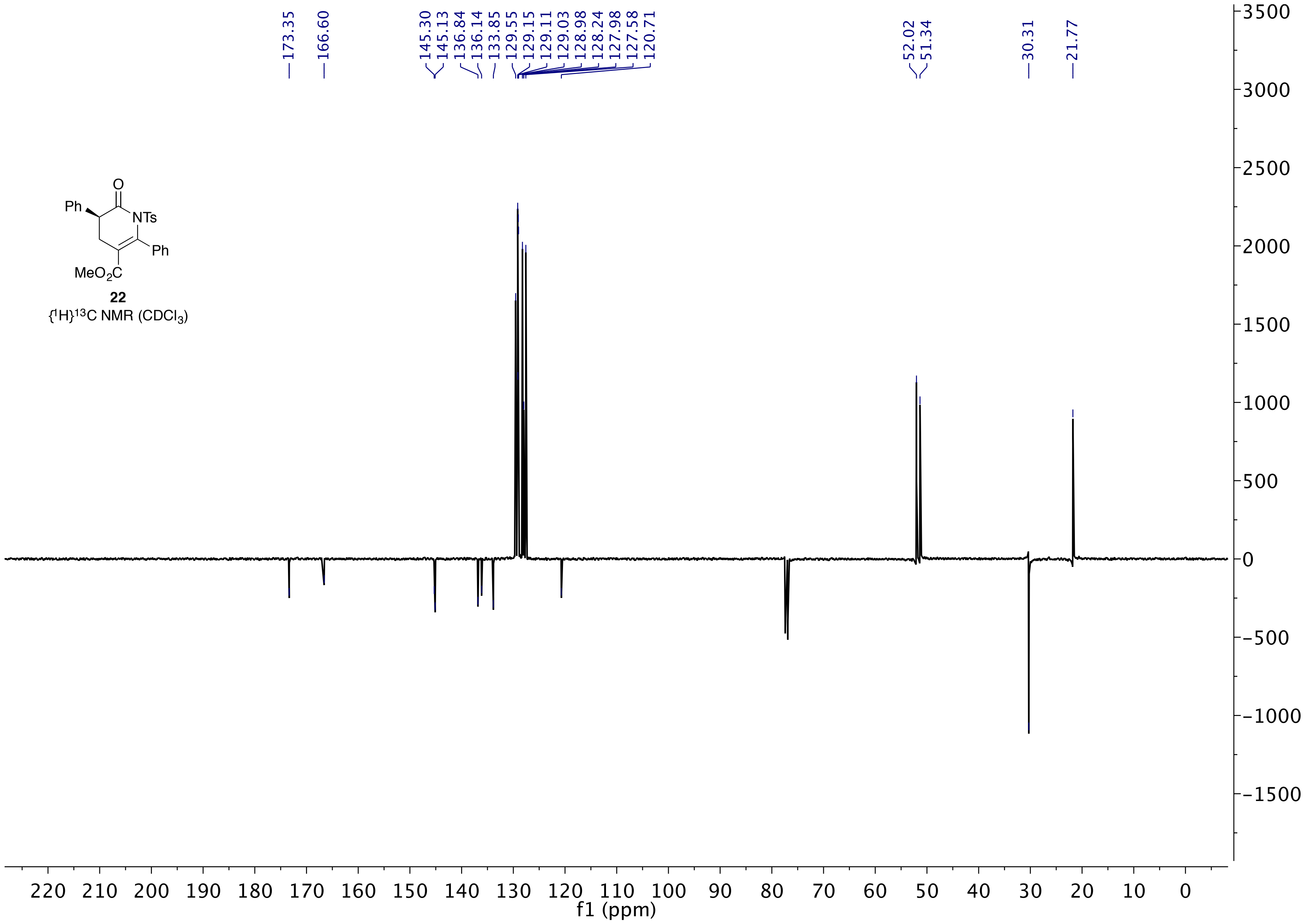

22

{¹H}¹³C NMR ($CDCl_3$)

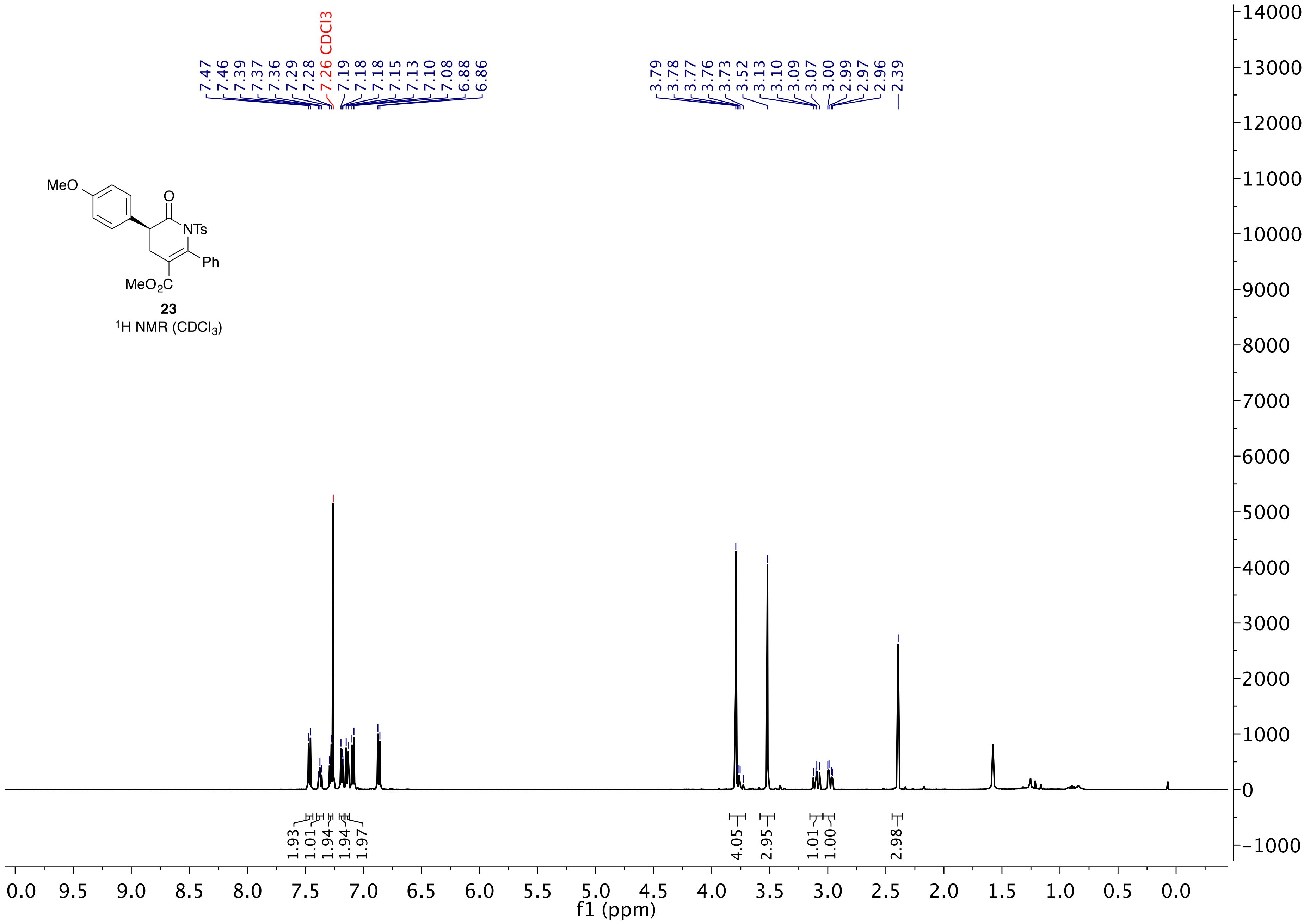
MeO
O
NTs
Ph
MeO2C
23
1H NMR (CDCl3)
7.47 7.46 7.39 7.37 7.36 7.29 7.28 7.26 CDCl3 7.19 7.18 7.18 7.15 7.13 7.10 7.08 6.88 6.86
3.79 3.78 3.77 3.76 3.73 3.52 3.13 3.10 3.09 3.07 3.00 2.99 2.97 2.96 2.39
1.93 1.01 1.94 1.94 1.97
4.05 2.95 1.01 1.00 2.98
f1 (ppm)

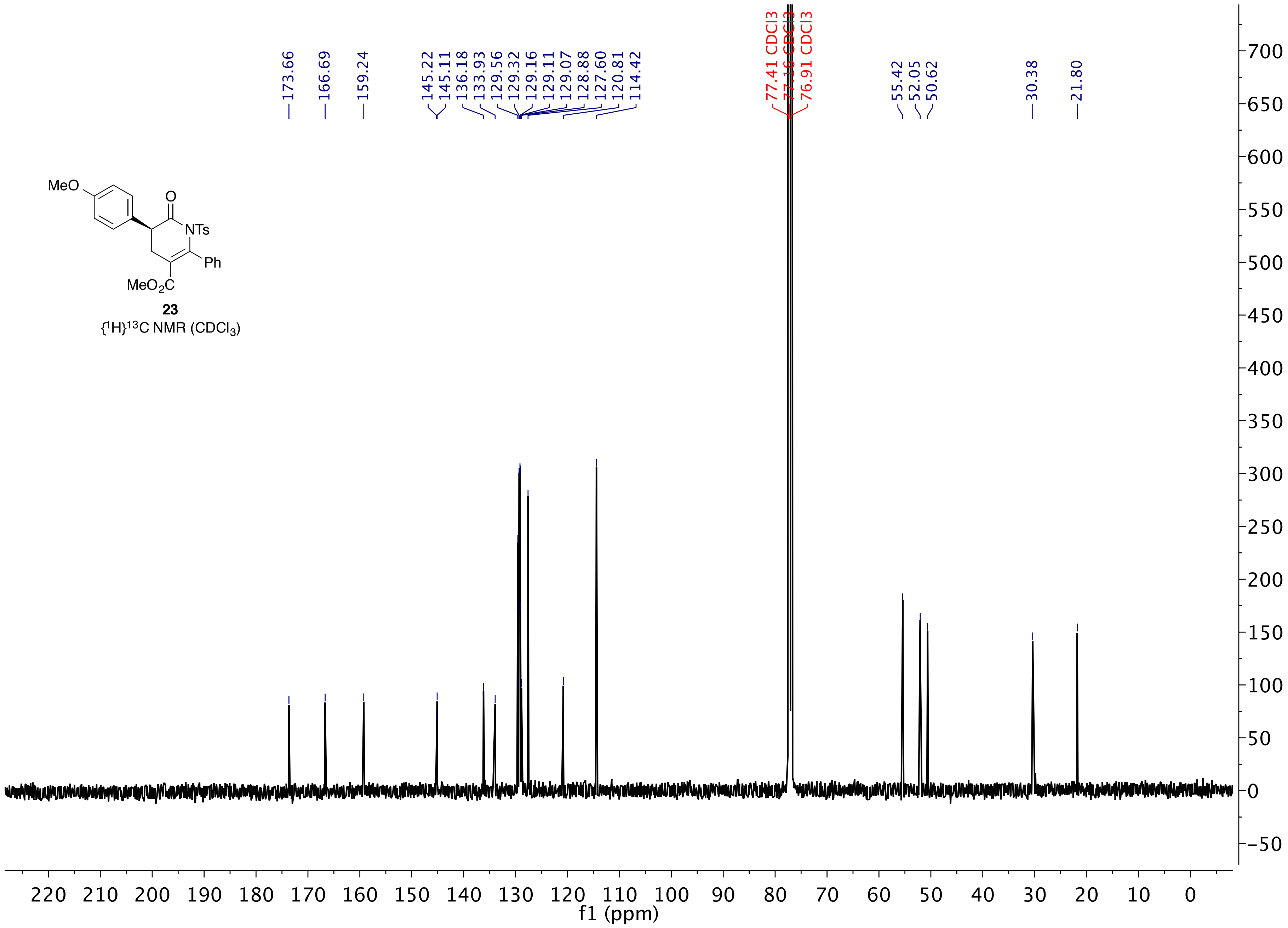

23

{^{1}H}^{13}C NMR ($CDCl_3$)

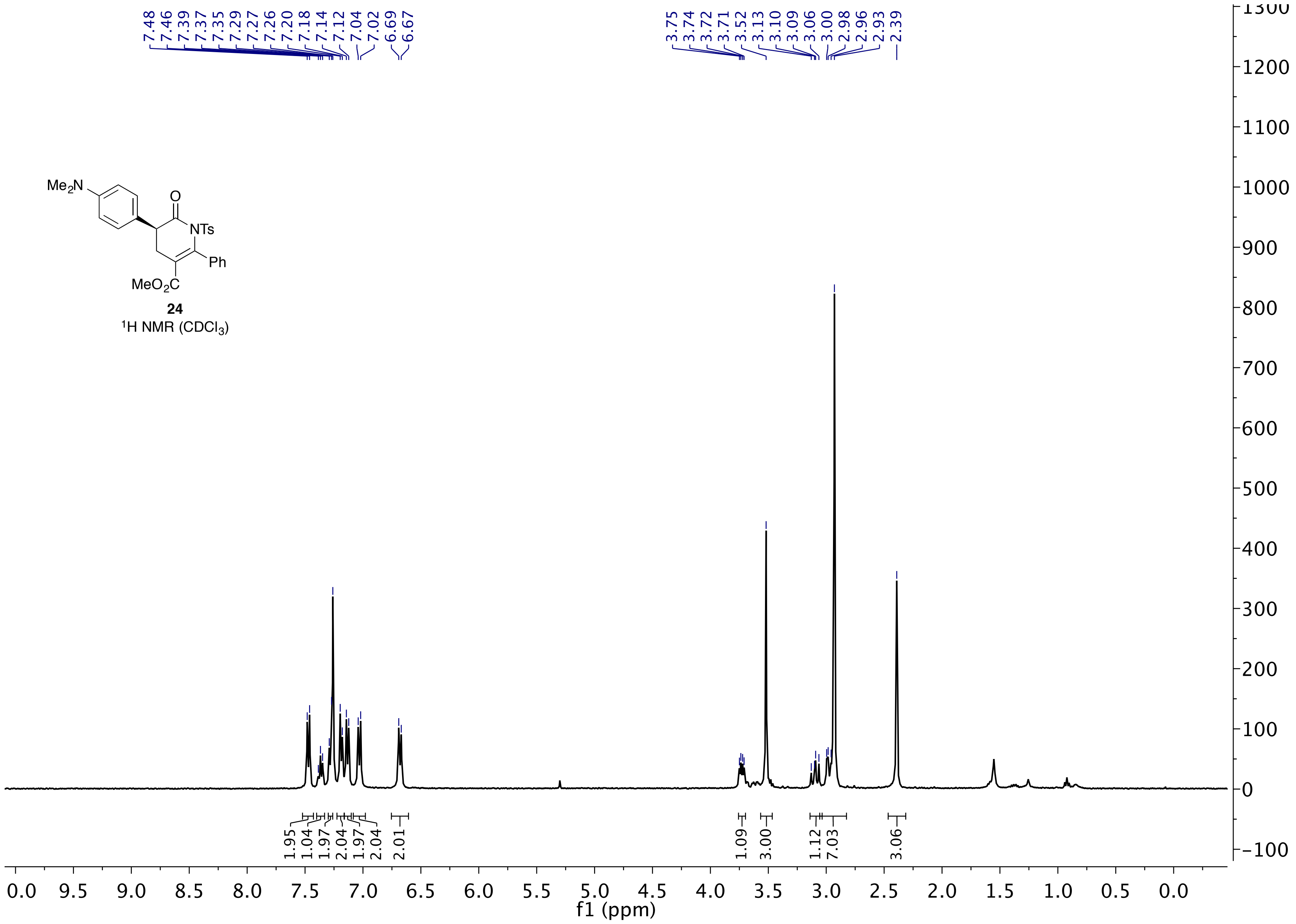

24
^{1}H NMR ($CDCl_3$)

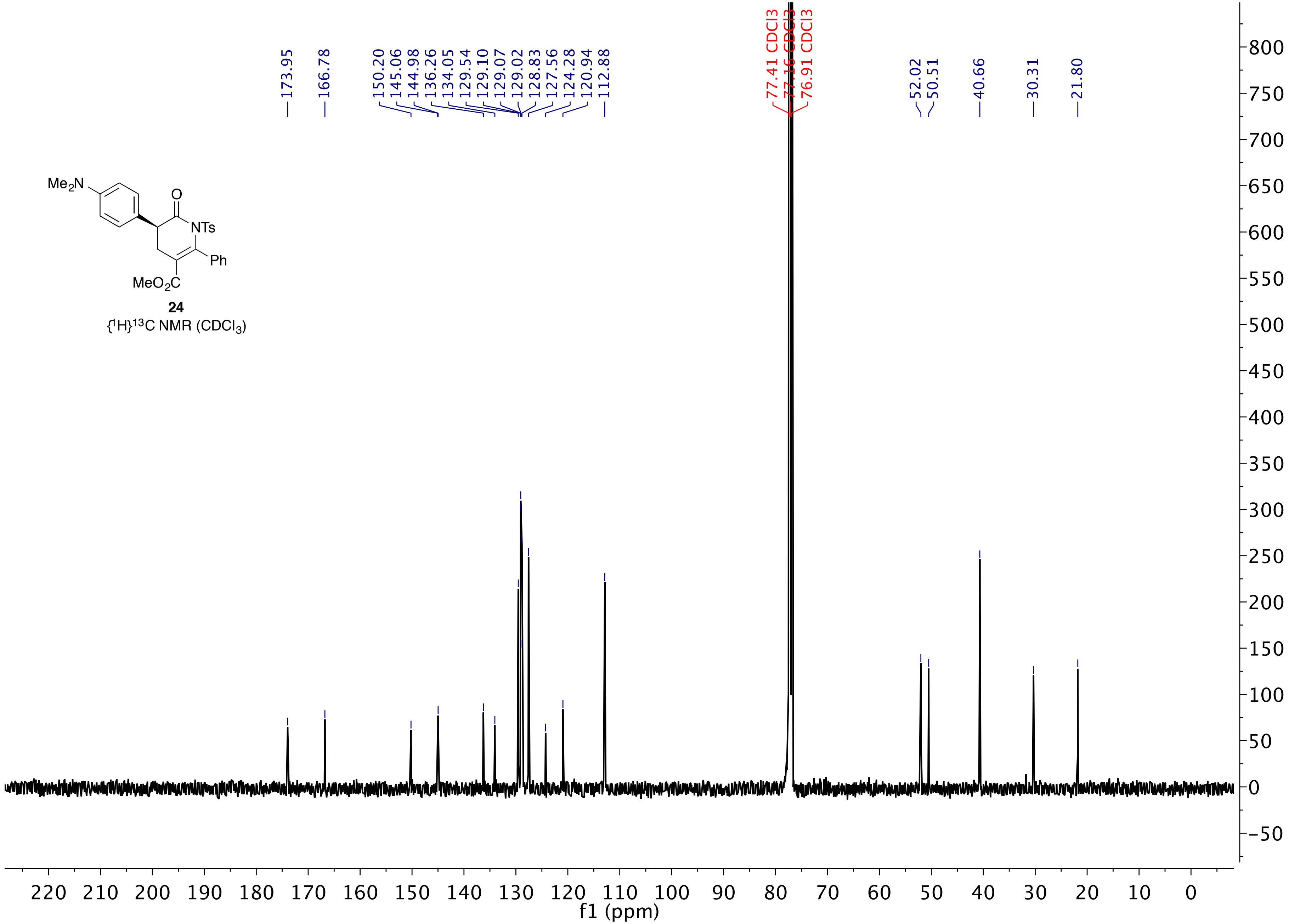

24

{^{1}H}^{13}C NMR ($CDCl_3$)

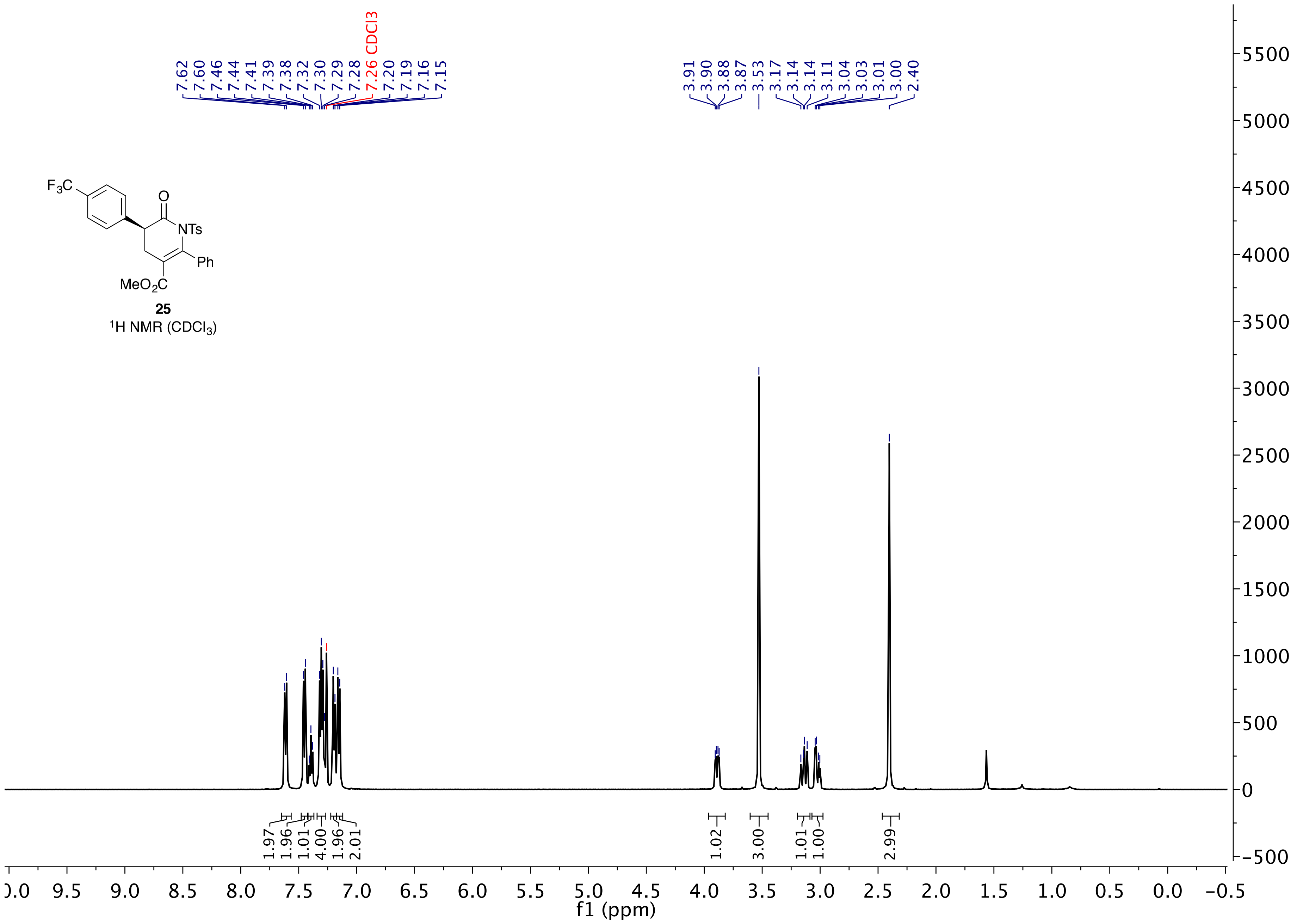

F3C
O
NTs
Ph
MeO2C
25
1H NMR (CDCl3)
7.62 7.60 7.46 7.44 7.41 7.39 7.38 7.32 7.30 7.29 7.28 7.26 CDCl3 7.20 7.19 7.16 7.15
3.91 3.90 3.88 3.87 3.53 3.17 3.14 3.14 3.11 3.04 3.03 3.01 3.00 2.40
1.97 1.96 1.01 4.00 1.96 2.01
1.02 3.00 1.01 1.00 2.99
f1 (ppm)

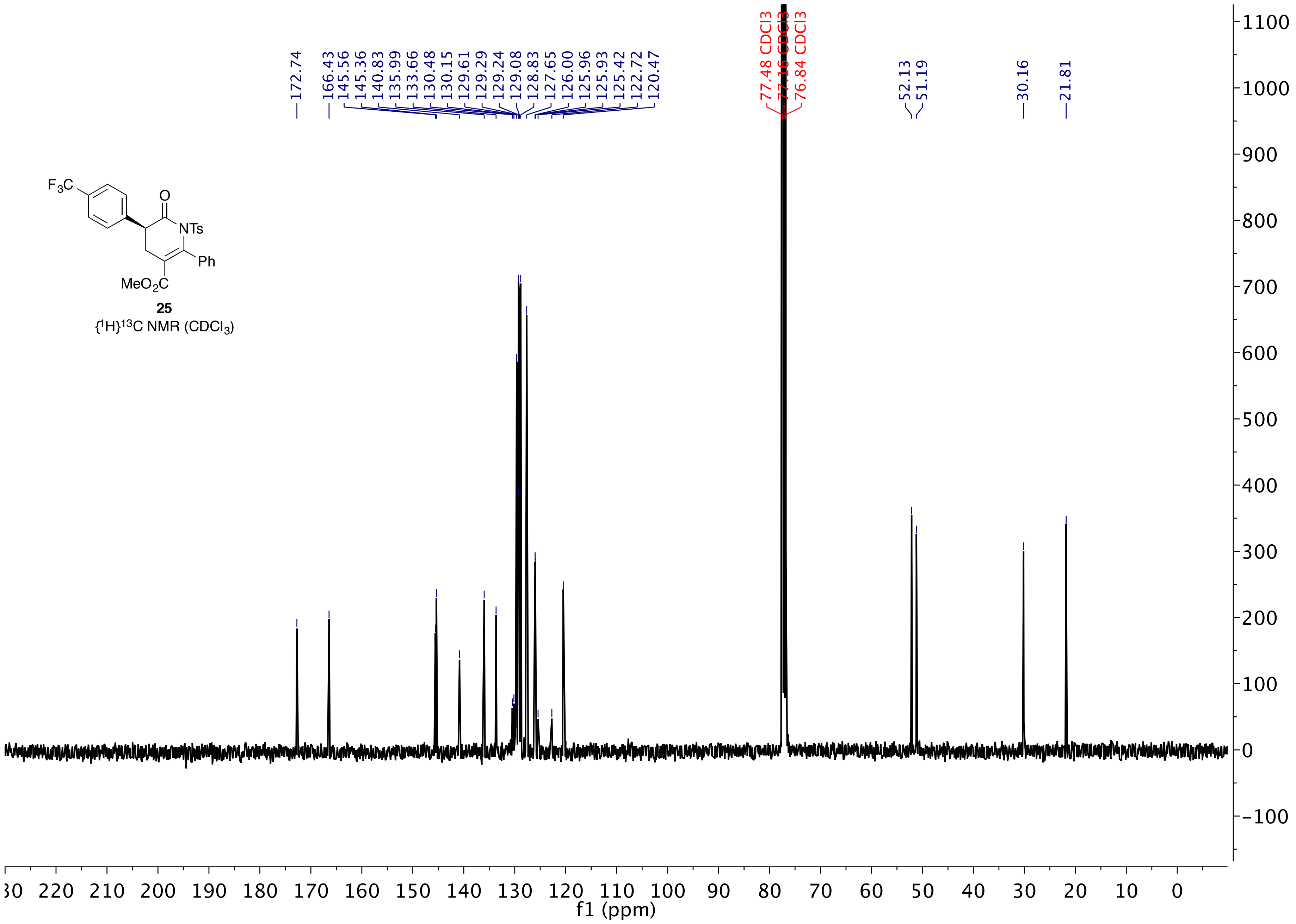
F3C
O
NTs
Ph
MeO2C
25
{1H}13C NMR (CDCl3)
172.74
166.43
145.56
145.36
140.83
135.99
133.66
130.48
130.15
129.61
129.29
129.24
129.08
128.83
127.65
126.00
125.96
125.93
125.42
122.72
120.47
77.48 CDCl3
77.16 CDCl3
76.84 CDCl3
52.13
51.19
30.16
21.81
f1 (ppm)

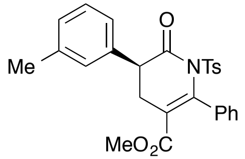

26

^{1}H NMR ($CDCl_3$)

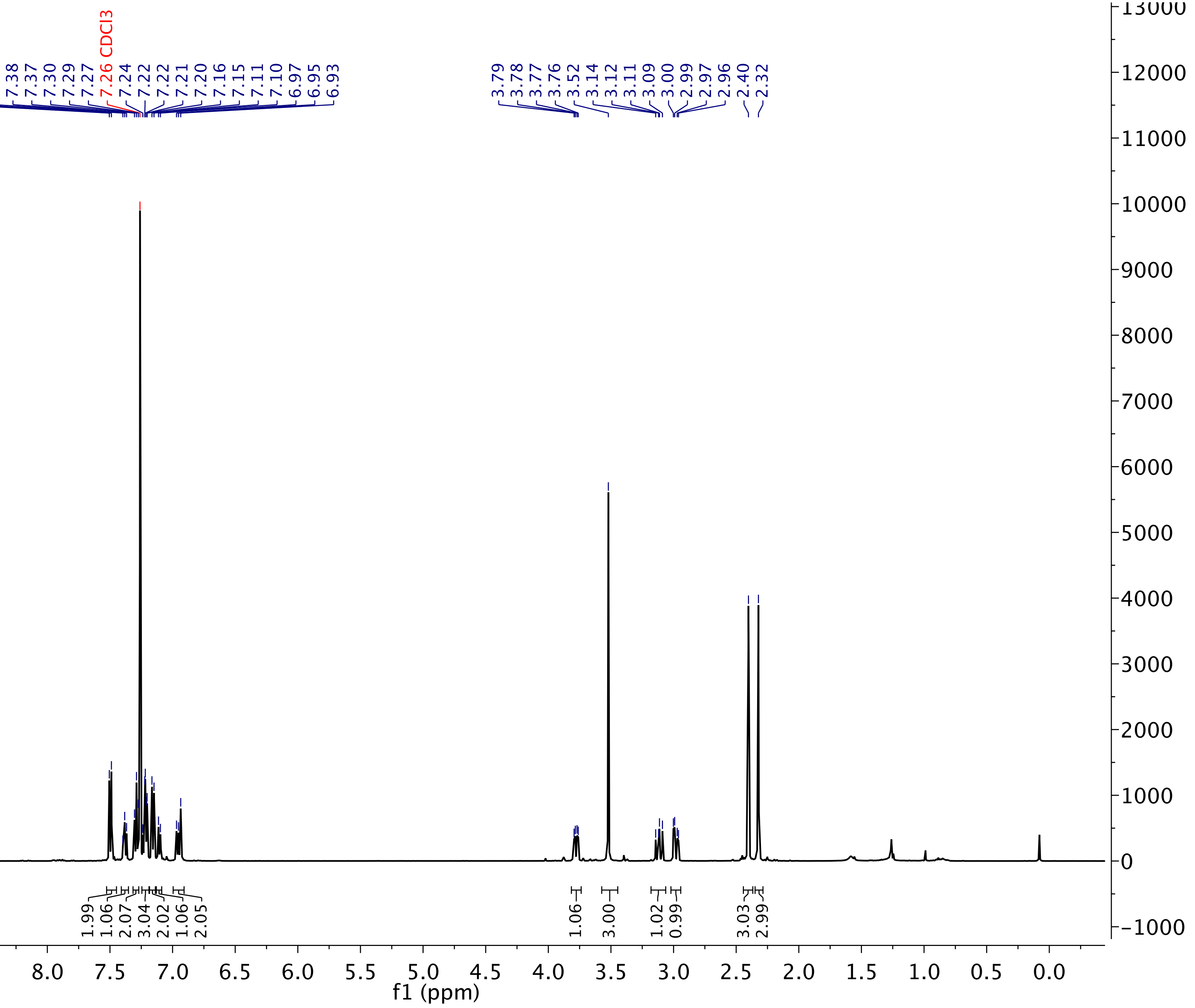

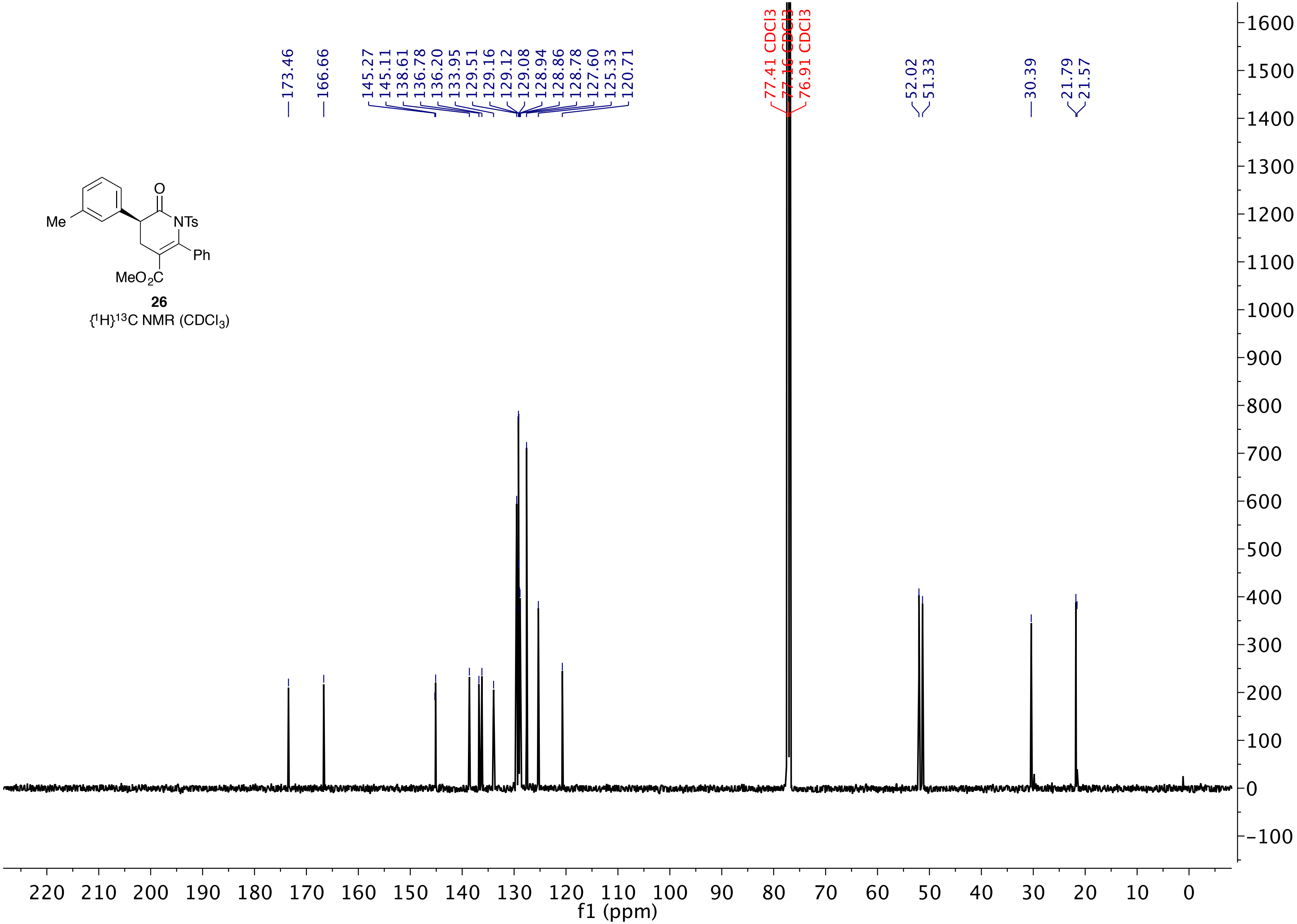

Me
O
NTs
Ph
MeO2C
26
{1H}13C NMR (CDCl3)
173.46
166.66
145.27
145.11
138.61
136.78
136.20
133.95
129.51
129.16
129.12
129.08
128.94
128.86
128.78
127.60
125.33
120.71
77.41 CDCl3
77.16 CDCl3
76.91 CDCl3
52.02
51.33
30.39
21.79
21.57
f1 (ppm)

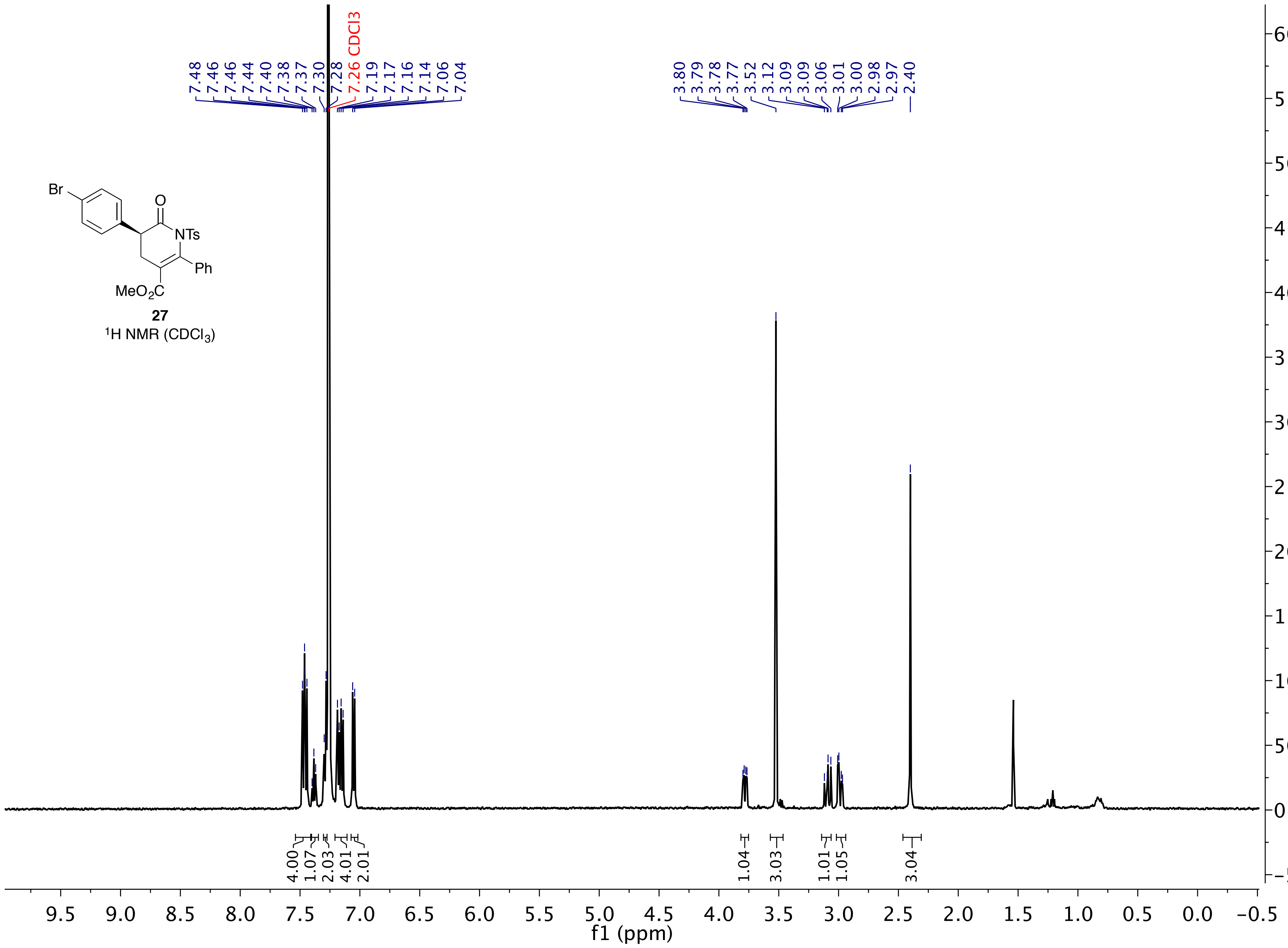

Br
O
NTs
Ph
MeO$_2$C
27
^{1}H NMR (CDCl$_3$)
7.48
7.46
7.46
7.44
7.40
7.38
7.37
7.30
7.28
7.26 CDCl3
7.19
7.17
7.16
7.14
7.06
7.04
3.80
3.79
3.78
3.77
3.52
3.12
3.09
3.09
3.06
3.01
3.00
2.98
2.97
2.40
4.00
1.07
2.03
4.01
2.01
1.04
3.03
1.01
1.05
3.04
9.5 9.0 8.5 8.0 7.5 7.0 6.5 6.0 5.5 5.0 4.5 4.0 3.5 3.0 2.5 2.0 1.5 1.0 0.5 0.0 −0.5
f1 (ppm)
600 550 500 450 400 350 300 250 200 150 100 50 0 −50

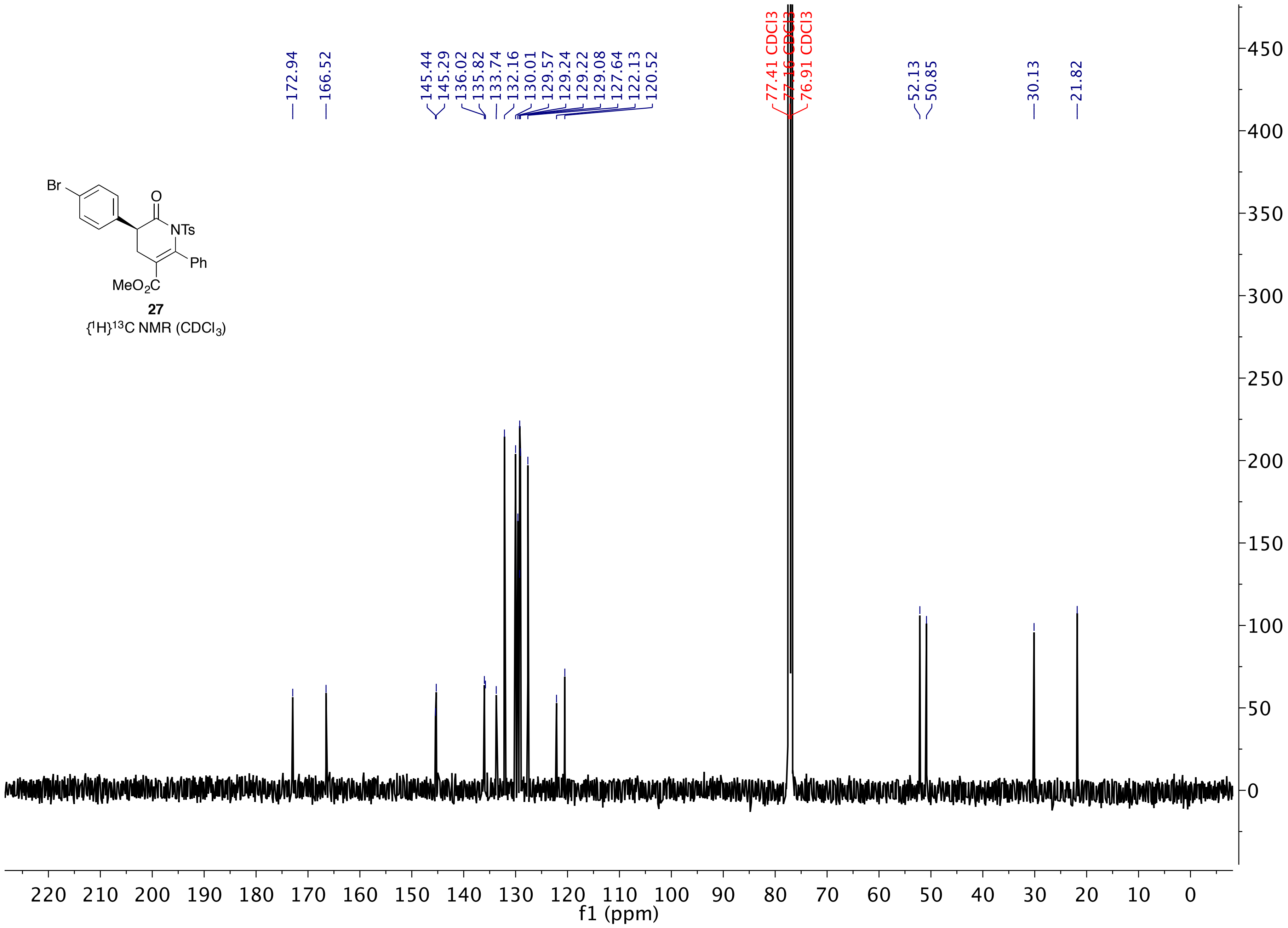
Br
O
NTs
Ph
MeO2C
27
{1H}13C NMR (CDCl3)
172.94
166.52
145.44
145.29
136.02
135.82
133.74
132.16
130.01
129.57
129.24
129.22
129.08
127.64
122.13
120.52
77.41 CDCl3
77.16 CDCl3
76.91 CDCl3
52.13
50.85
30.13
21.82
f1 (ppm)

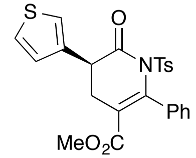

28
^1H NMR ($CDCl_3$)

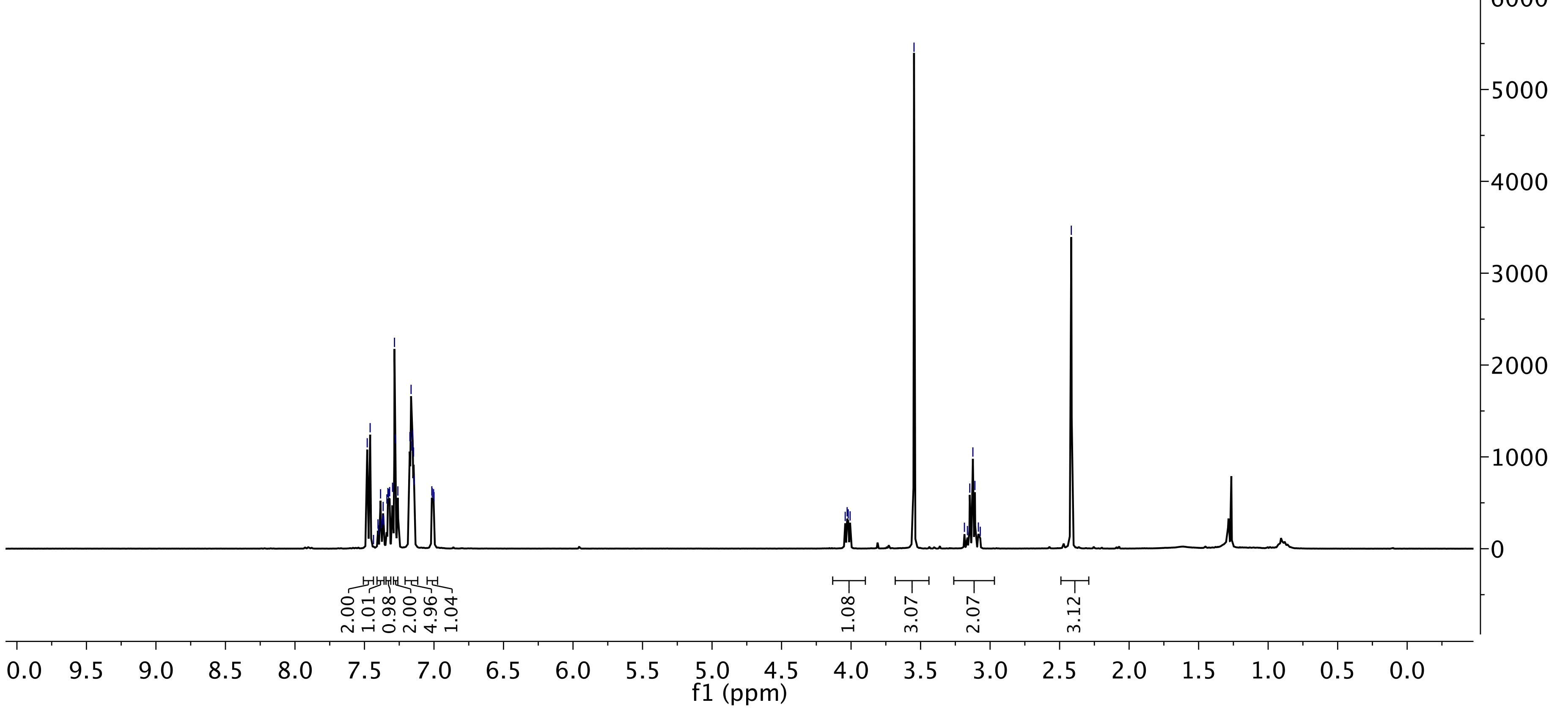

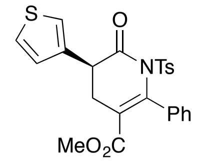

28

{1H}13C NMR ($CDCl_3$)

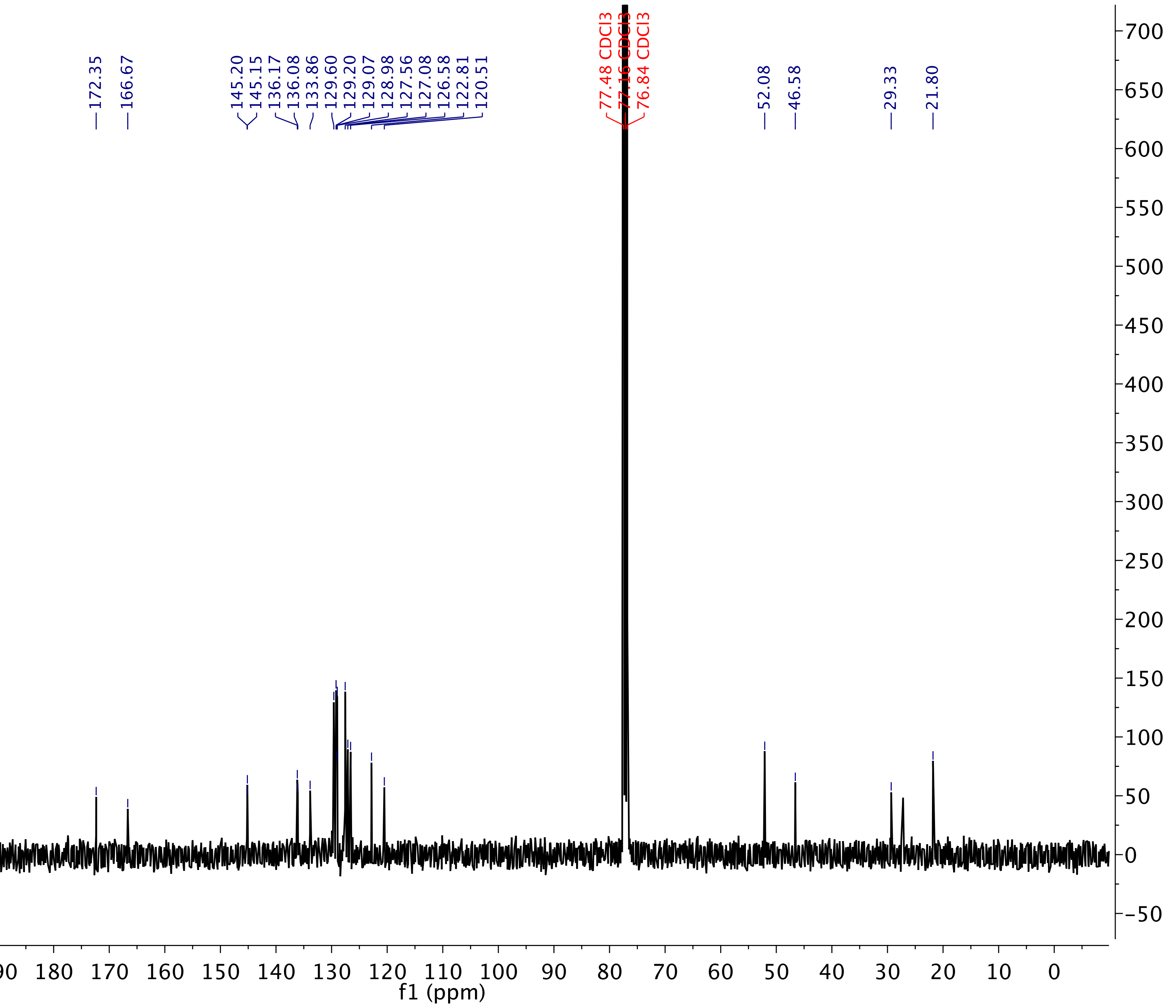

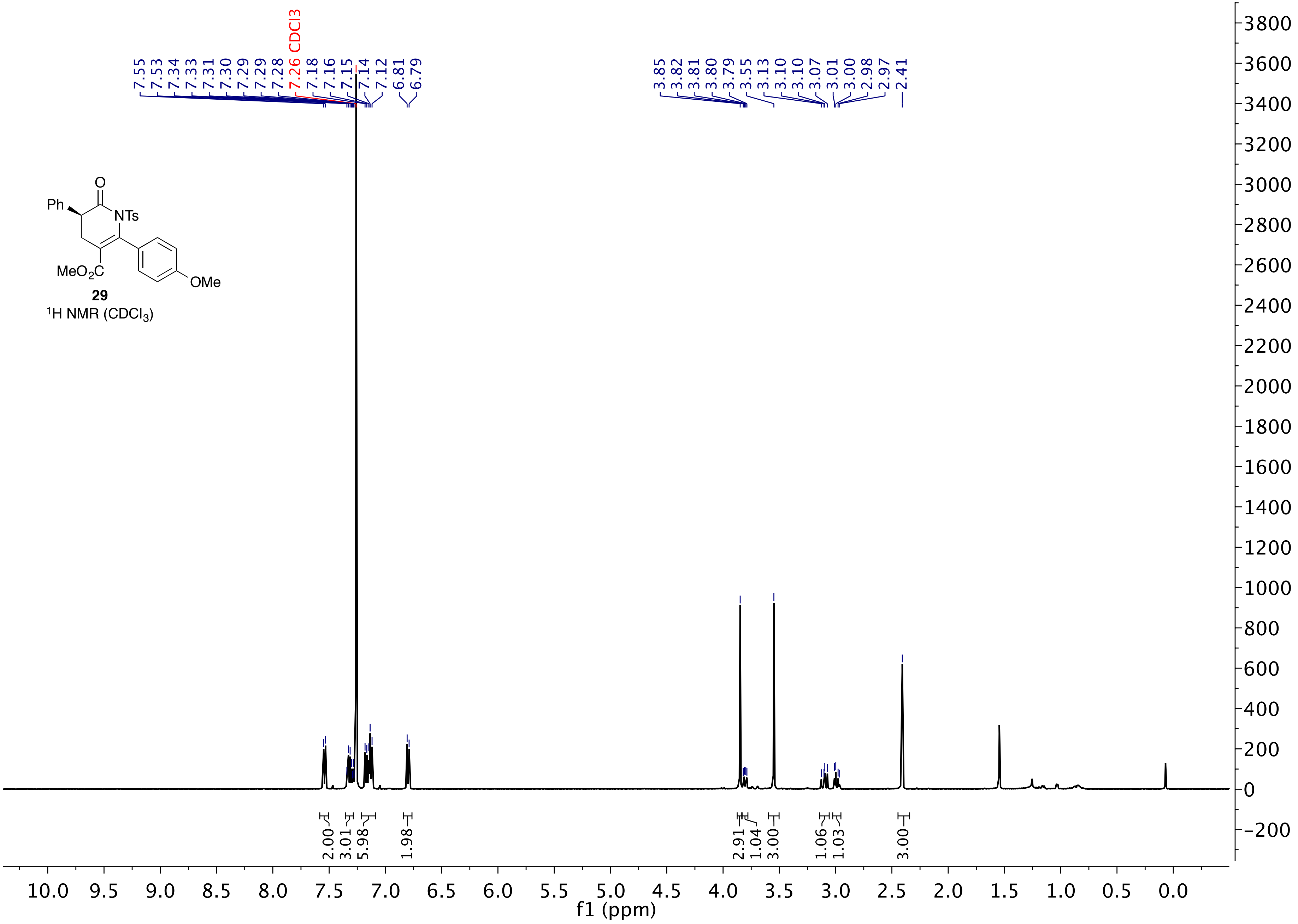

7.55
7.53
7.34
7.33
7.31
7.30
7.29
7.29
7.28
7.26 CDCl3
7.18
7.16
7.15
7.14
7.12
6.81
6.79
3.85
3.82
3.81
3.80
3.79
3.55
3.13
3.10
3.10
3.07
3.01
3.00
2.98
2.97
2.41
Ph
O
NTs
MeO2C
OMe
29
1H NMR (CDCl3)
2.00
3.01
5.98
1.98
2.91
1.04
3.00
1.06
1.03
3.00
10.0 9.5 9.0 8.5 8.0 7.5 7.0 6.5 6.0 5.5 5.0 4.5 4.0 3.5 3.0 2.5 2.0 1.5 1.0 0.5 0.0
f1 (ppm)
3800 3600 3400 3200 3000 2800 2600 2400 2200 2000 1800 1600 1400 1200 1000 800 600 400 200 0 −200

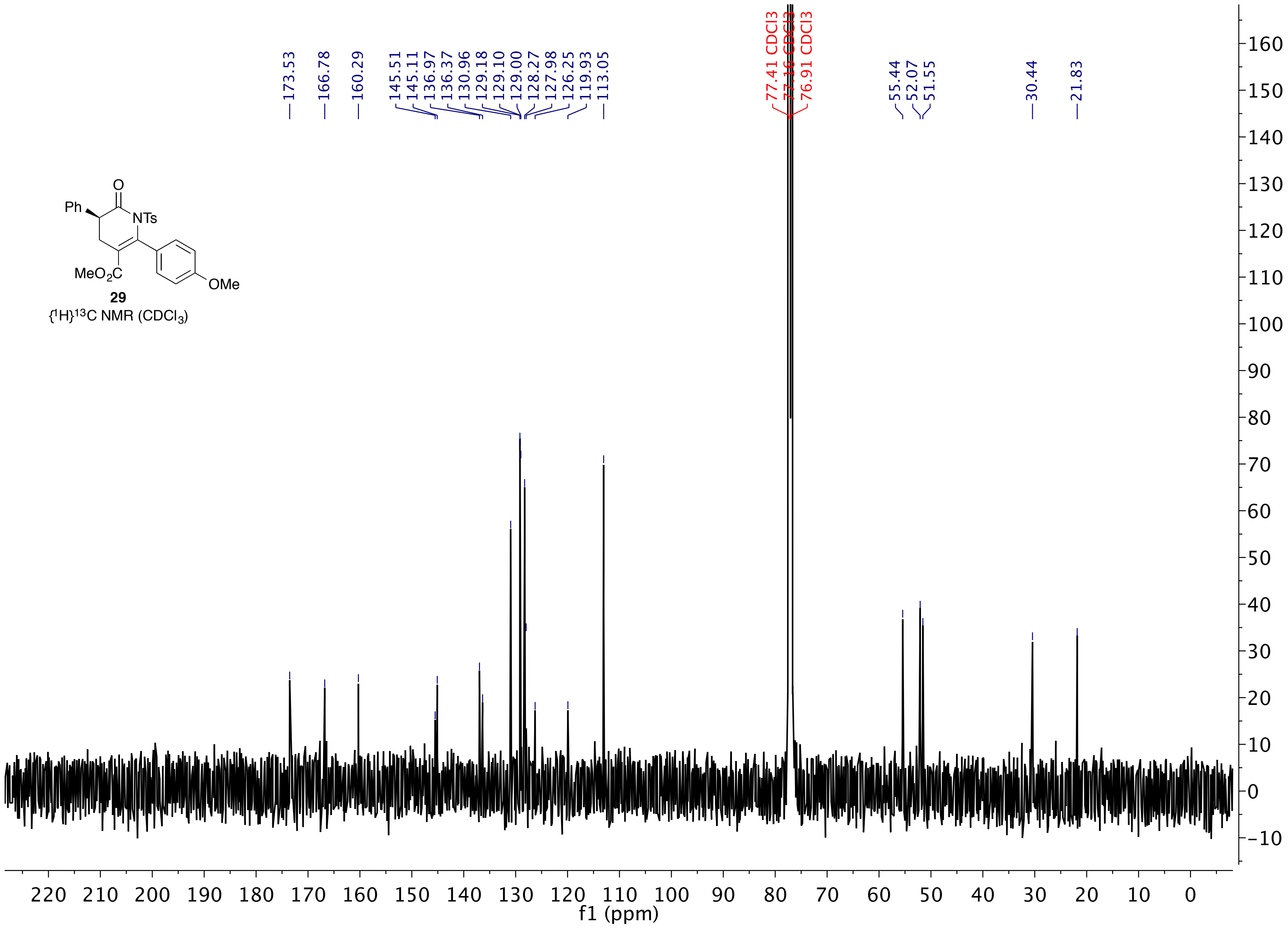
Ph
O
NTs
MeO2C
OMe
29
{1H}13C NMR (CDCl3)
173.53
166.78
160.29
145.51
145.11
136.97
136.37
130.96
129.18
129.10
129.00
128.27
127.98
126.25
119.93
113.05
77.41 CDCl3
77.16 CDCl3
76.91 CDCl3
55.44
52.07
51.55
30.44
21.83
f1 (ppm)

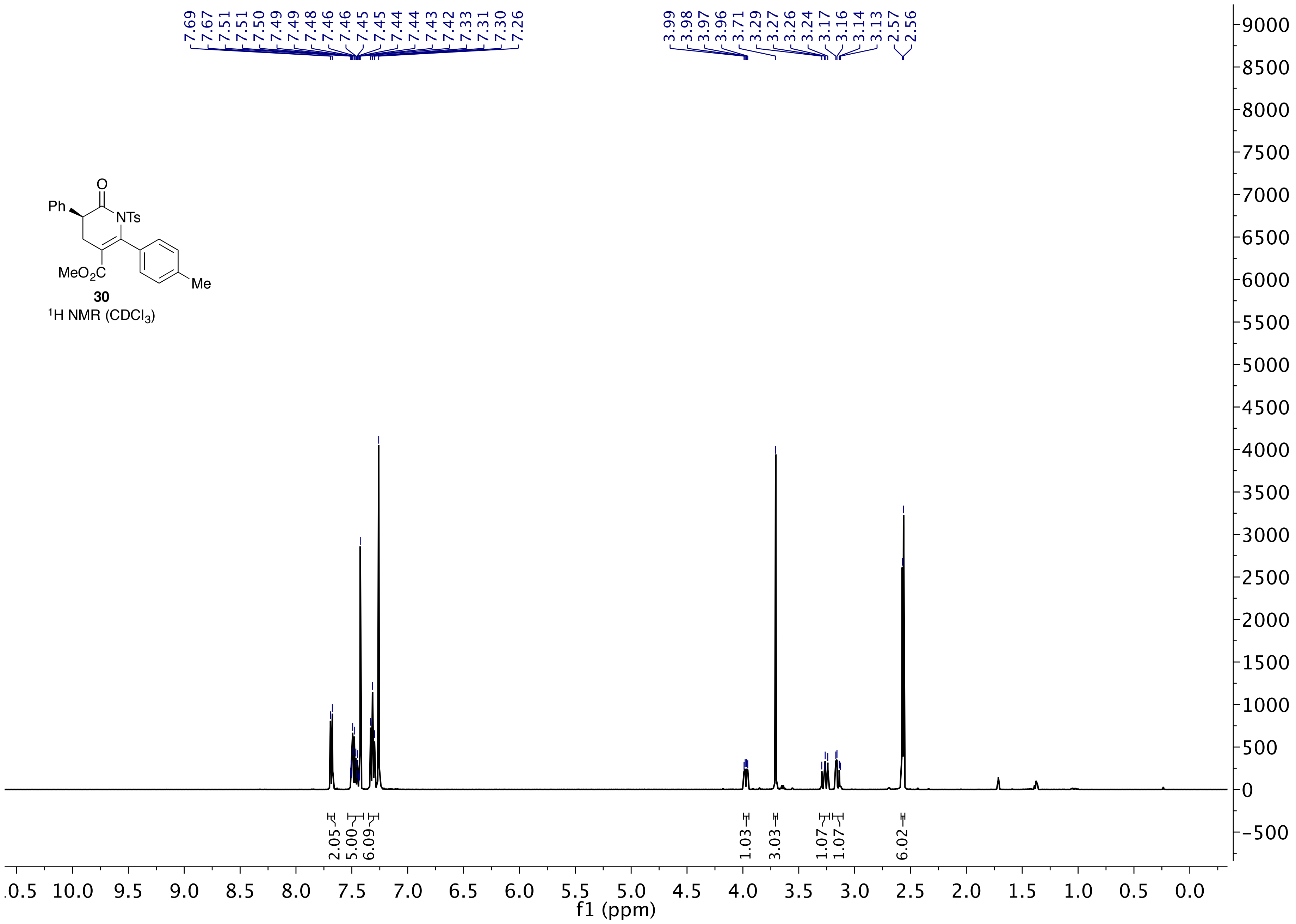
7.69 7.67 7.51 7.51 7.50 7.49 7.49 7.48 7.46 7.46 7.45 7.45 7.44 7.44 7.43 7.42 7.33 7.31 7.30 7.26
3.99 3.98 3.97 3.96 3.71 3.29 3.27 3.26 3.24 3.17 3.16 3.14 3.13 2.57 2.56
Ph
O
NTs
MeO2C
Me
30
1H NMR (CDCl3)
2.05 5.00 6.09 1.03 3.03 1.07 1.07 6.02
f1 (ppm)

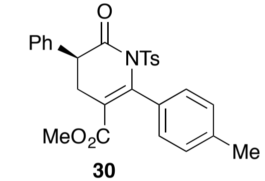

30

{^{1}H}^{13}C NMR ($CDCl_3$)

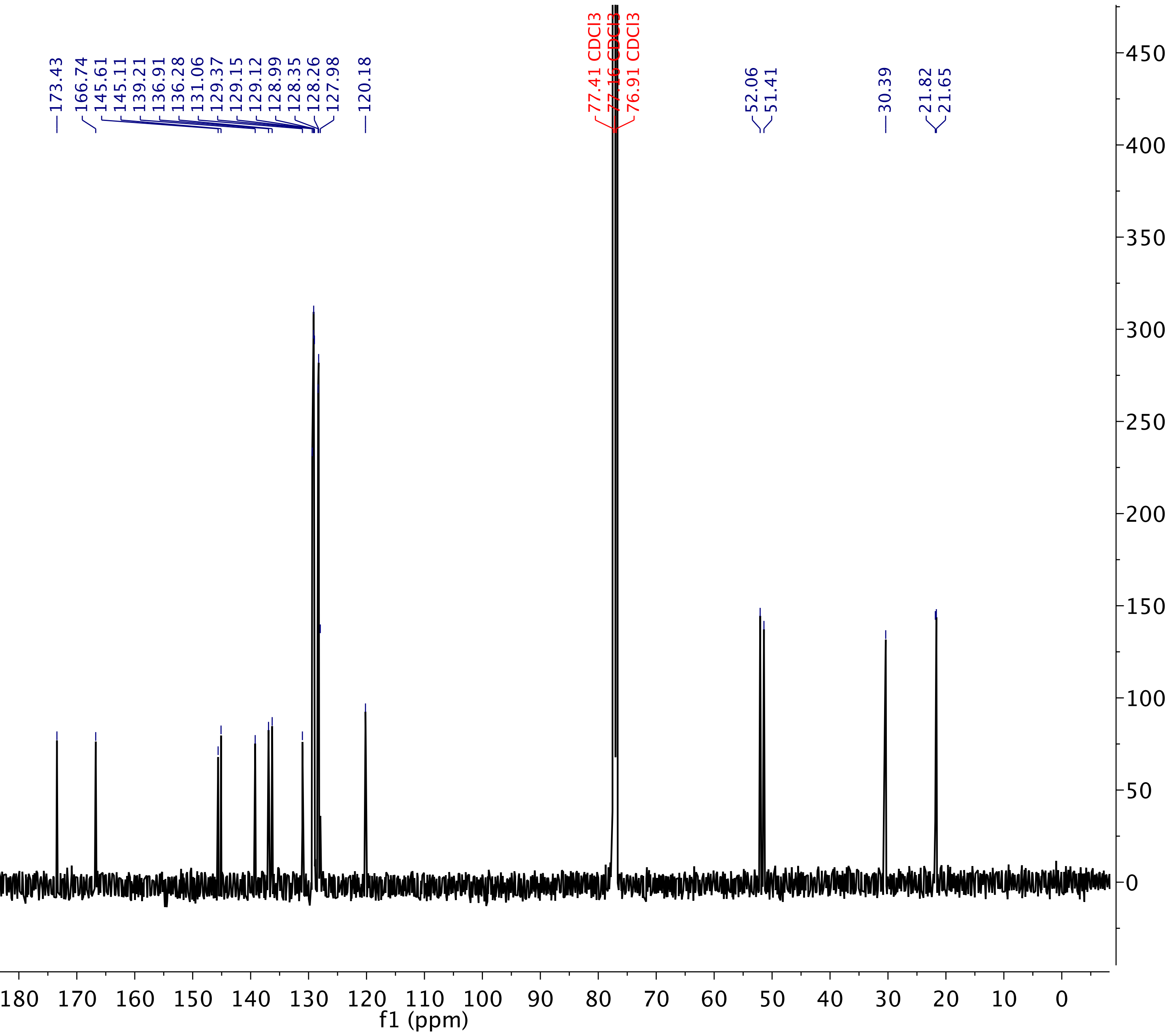

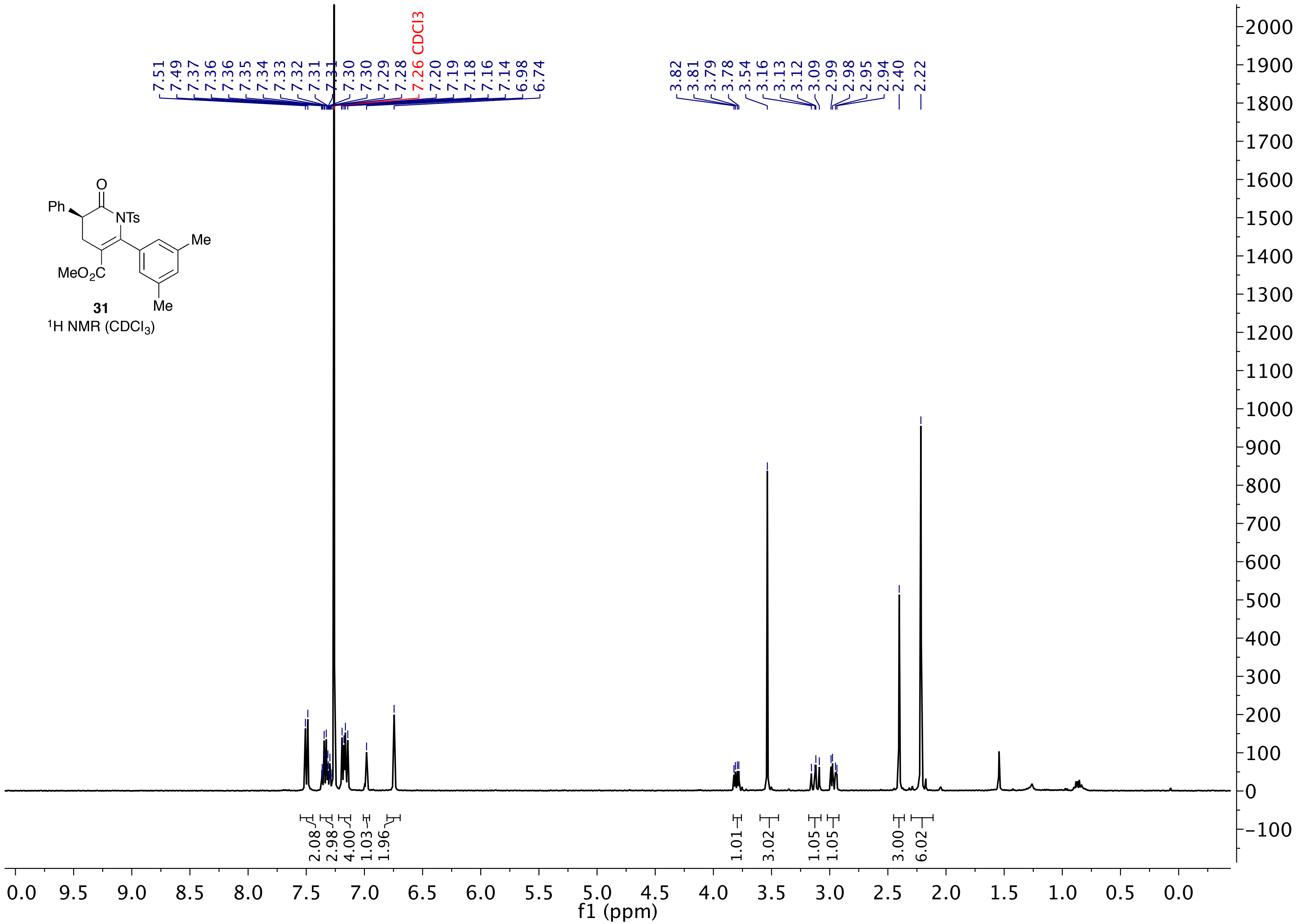
7.51
7.49
7.37
7.36
7.36
7.35
7.34
7.33
7.32
7.31
7.31
7.30
7.30
7.29
7.28
7.26 CDCl3
7.20
7.19
7.18
7.16
7.14
6.98
6.74
3.82
3.81
3.79
3.78
3.54
3.16
3.13
3.12
3.09
2.99
2.98
2.95
2.94
2.40
2.22
Ph
O
NTs
Me
MeO2C
Me
31
1H NMR (CDCl3)
2.08
2.98
4.00
1.03
1.96
1.01
3.02
1.05
1.05
3.00
6.02
f1 (ppm)

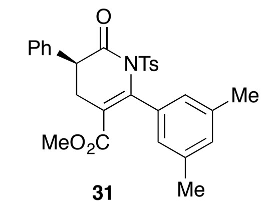

31

{^{1}H}^{13}C NMR ($CDCl_3$)

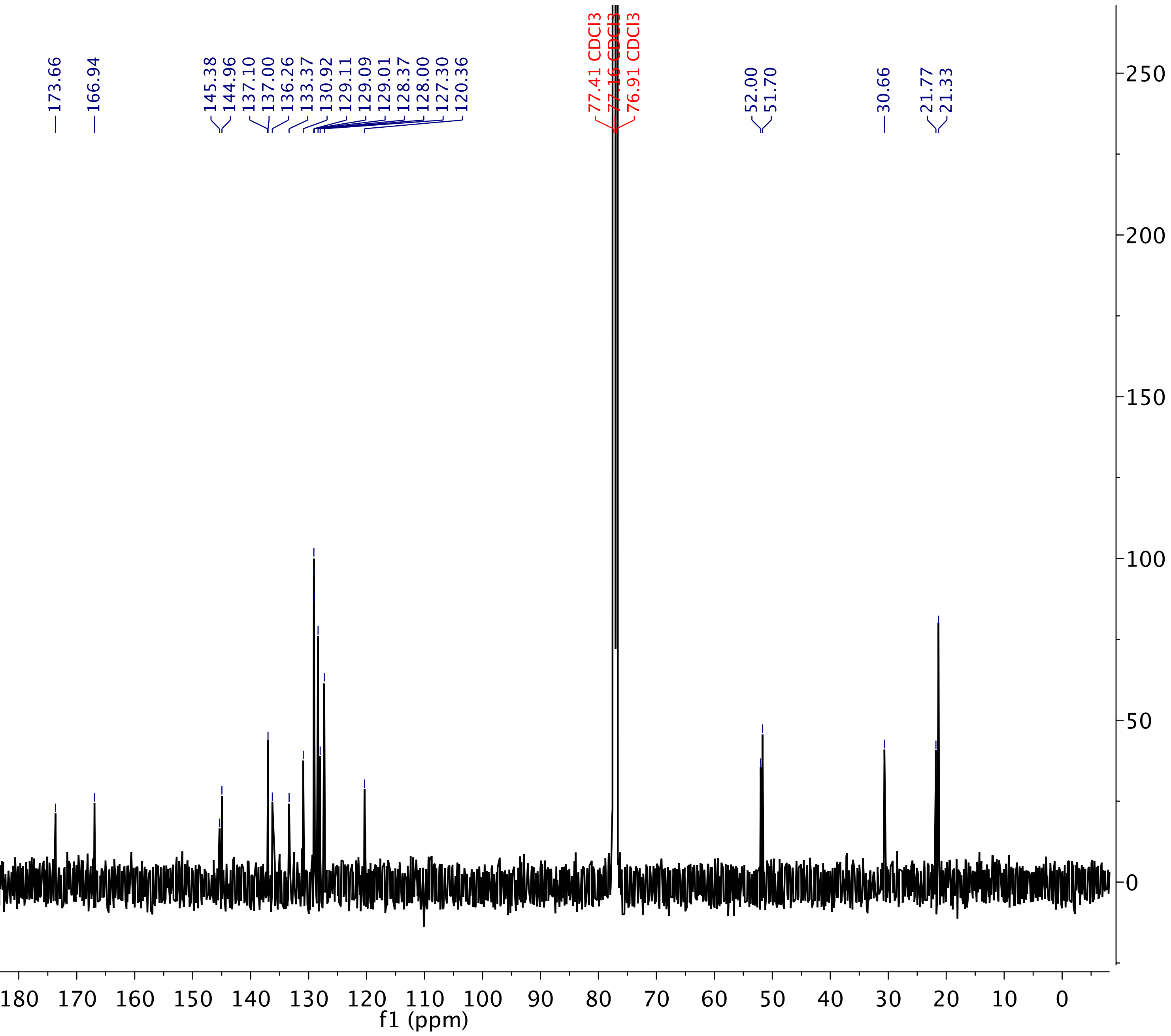

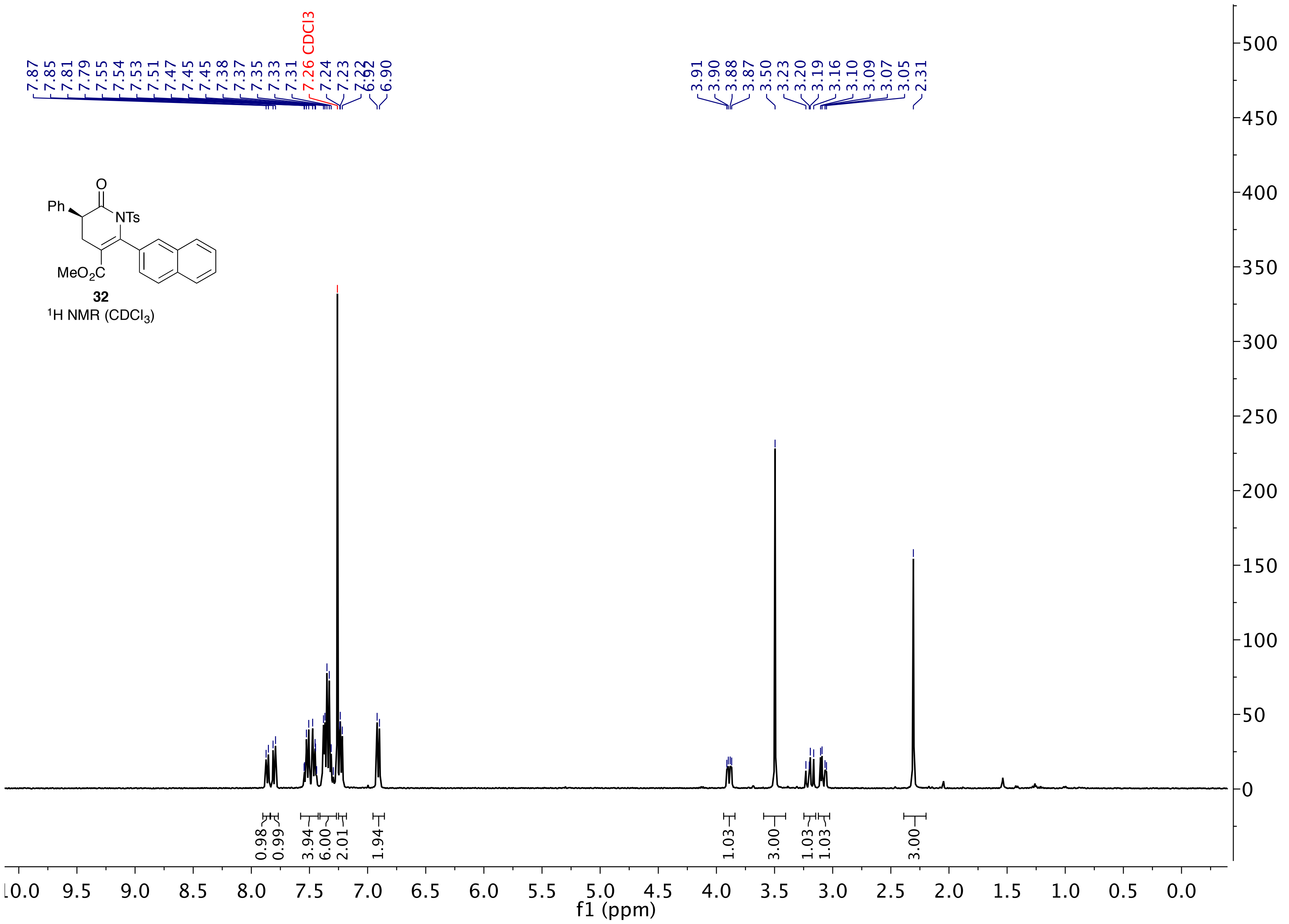
7.87
7.85
7.81
7.79
7.55
7.54
7.53
7.51
7.47
7.45
7.45
7.38
7.37
7.35
7.33
7.31
7.26 CDCl3
7.24
7.23
7.22
6.92
6.90
3.91
3.90
3.88
3.87
3.50
3.23
3.20
3.19
3.16
3.10
3.09
3.07
3.05
2.31
Ph
O
NTs
MeO2C
32
1H NMR (CDCl3)
0.98
0.99
3.94
6.00
2.01
1.94
1.03
3.00
1.03
1.03
3.00
f1 (ppm)

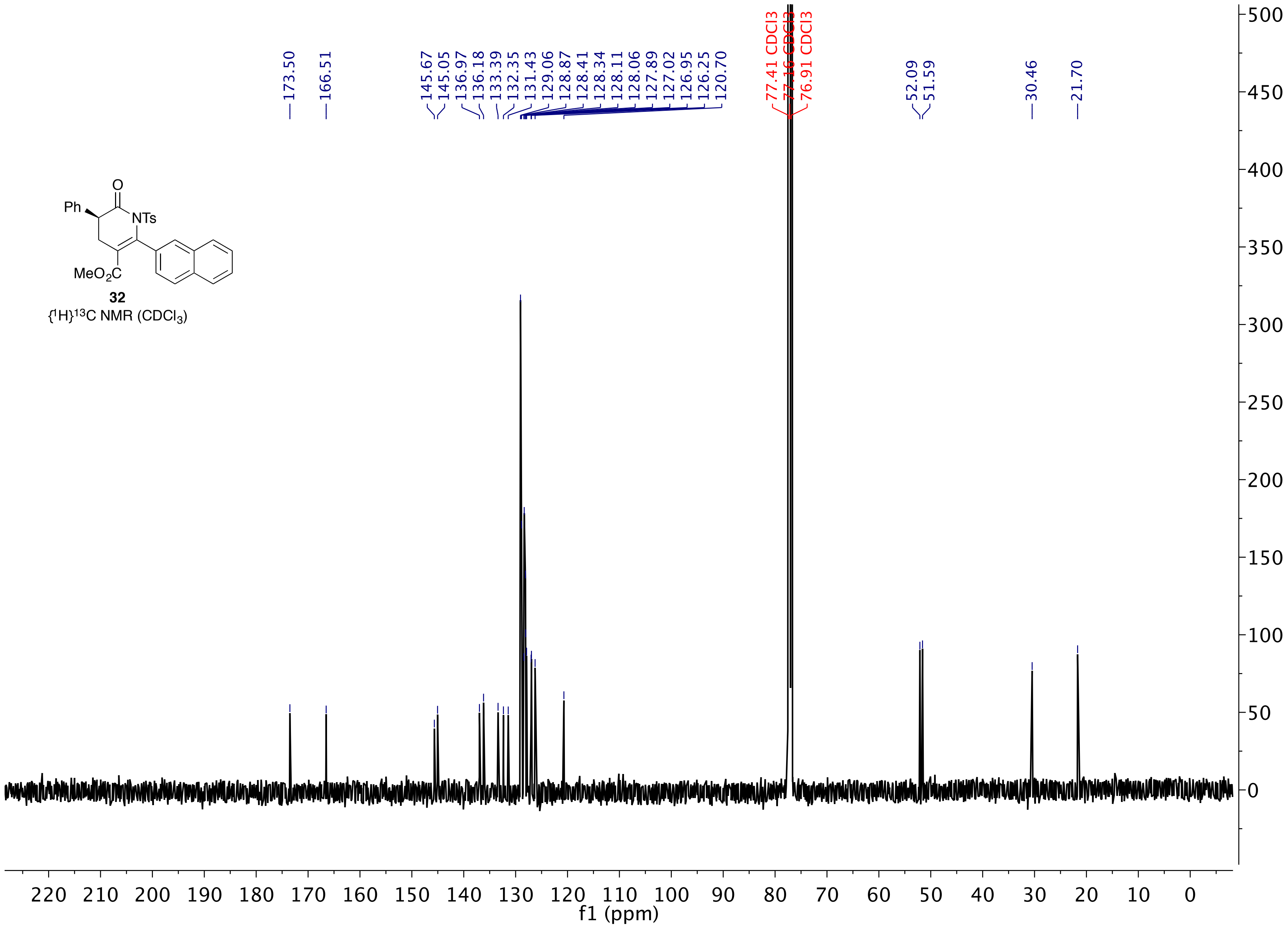
O
Ph
NTs
MeO2C
32
{1H}13C NMR (CDCl3)
173.50
166.51
145.67
145.05
136.97
136.18
133.39
132.35
131.43
129.06
128.87
128.41
128.34
128.11
128.06
127.89
127.02
126.95
126.25
120.70
77.41 CDCl3
77.16 CDCl3
76.91 CDCl3
52.09
51.59
30.46
21.70
f1 (ppm)

5.0 Chiral HPLC Analysis

HPLC data for **6**: Chiralpak AD-H (80:20 hexane:IPA, flow rate 1 mLmin^{-1}, 254 nm, 30 °C) t_R (3*R*): 13.6 min, t_R (3*S*): 15.5 min; 91% ee.

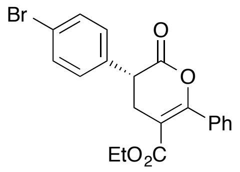

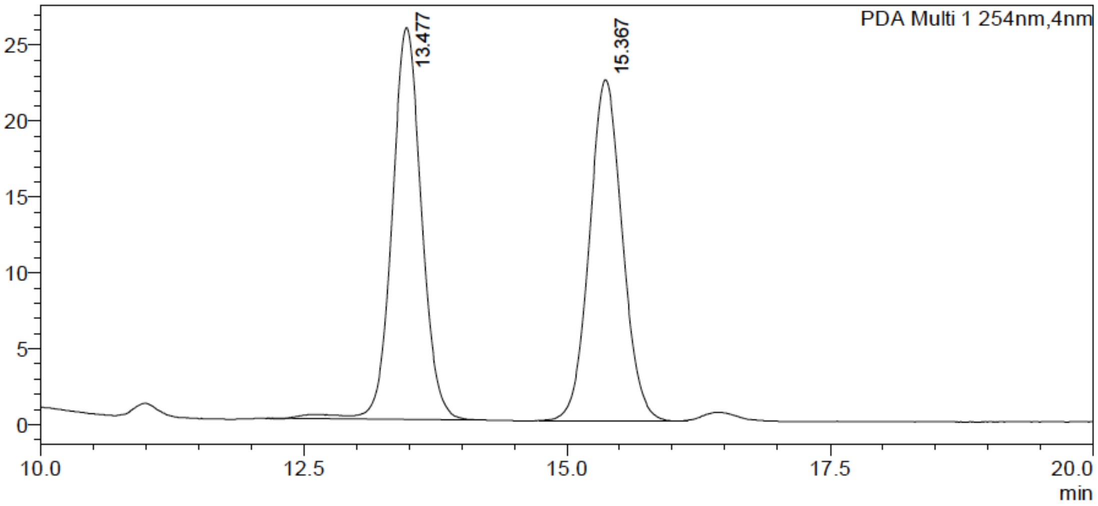

PDA Ch1 254nm

Peak#	Ret. Time	Area%
1	13.477	50.774
2	15.367	49.226
Total		100.000

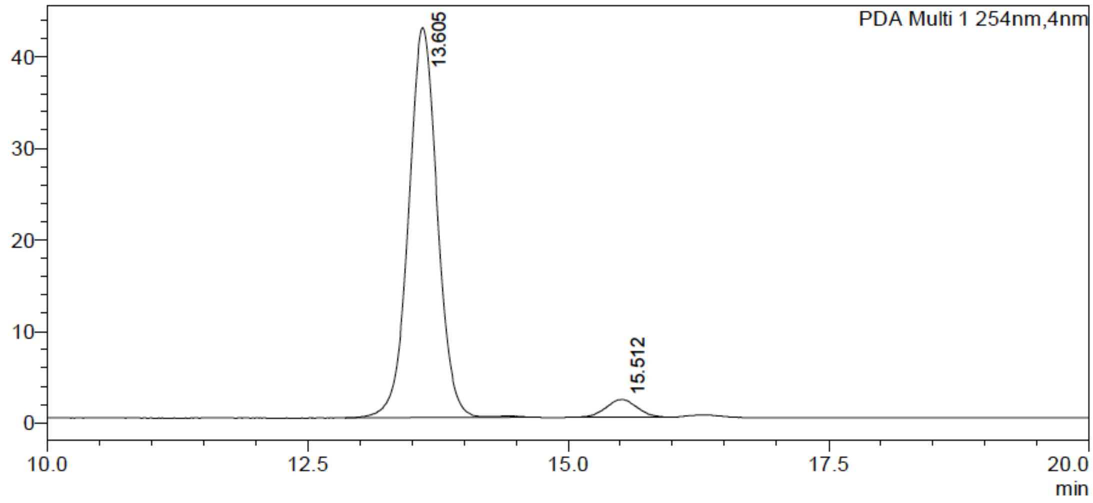

PDA Ch1 254nm

Peak#	Ret. Time	Area%
1	13.605	95.394
2	15.512	4.606
Total		100.000

HPLC data for **10**: Chiralpak AD-H (80:20 hexane:IPA, flow rate 1 mLmin^{-1}, 254 nm, 30 °C) t_R (3*R*): 33.7 min, t_R (3*S*): 37.5 min; 91% ee.

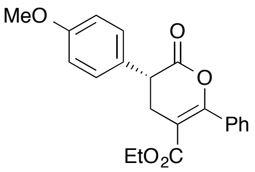

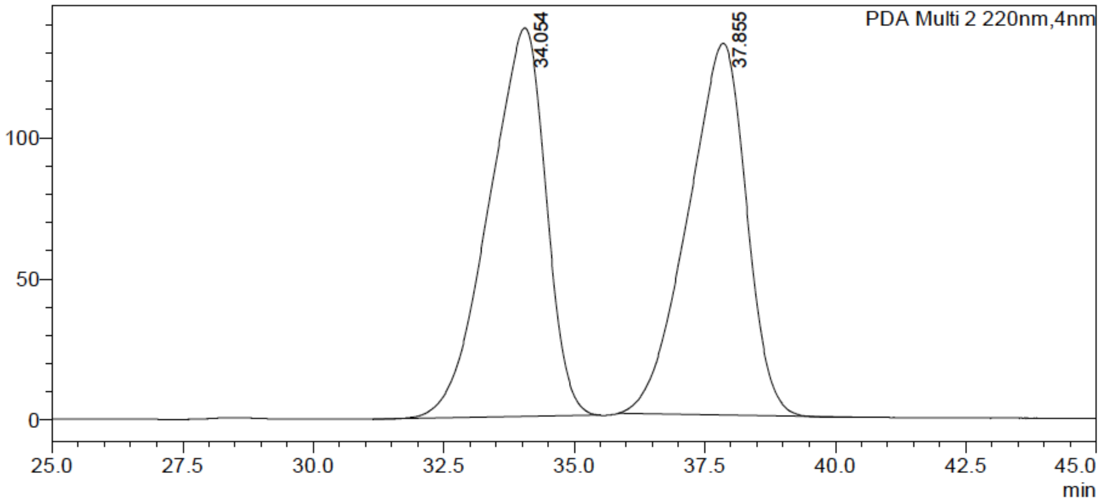

PDA Ch2 220nm

Peak#	Ret. Time	Area%
1	34.054	50.525
2	37.855	49.475
Total		100.000

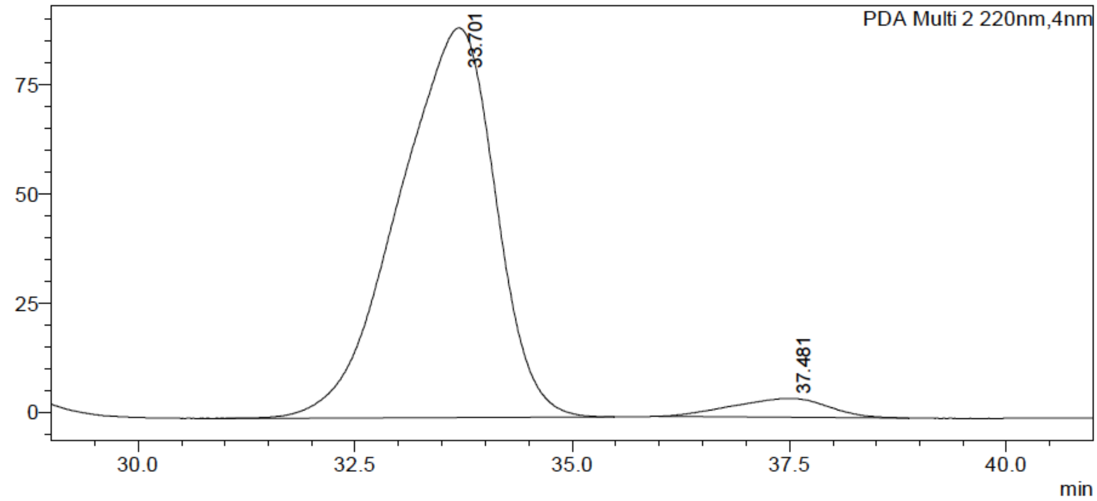

PDA Ch2 220nm

Peak#	Ret. Time	Area%
1	33.701	95.490
2	37.481	4.510
Total		100.000

HPLC data for **11**: Chiralpak AD-H (95:5 hexane:IPA, flow rate 1 mLmin^{-1}, 220 nm, 30 °C) t_R (3*R*): 29.9 min, t_R (3*S*): 34.3 min; 89% ee.

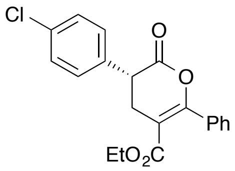

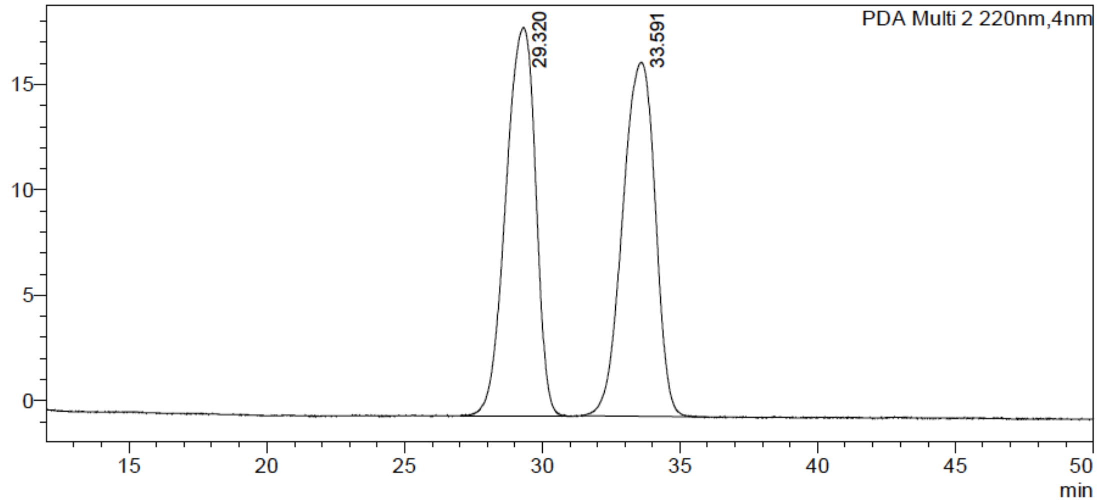

PDA Ch2 220nm

Peak#	Ret. Time	Area%
1	29.320	49.986
2	33.591	50.014
Total		100.000

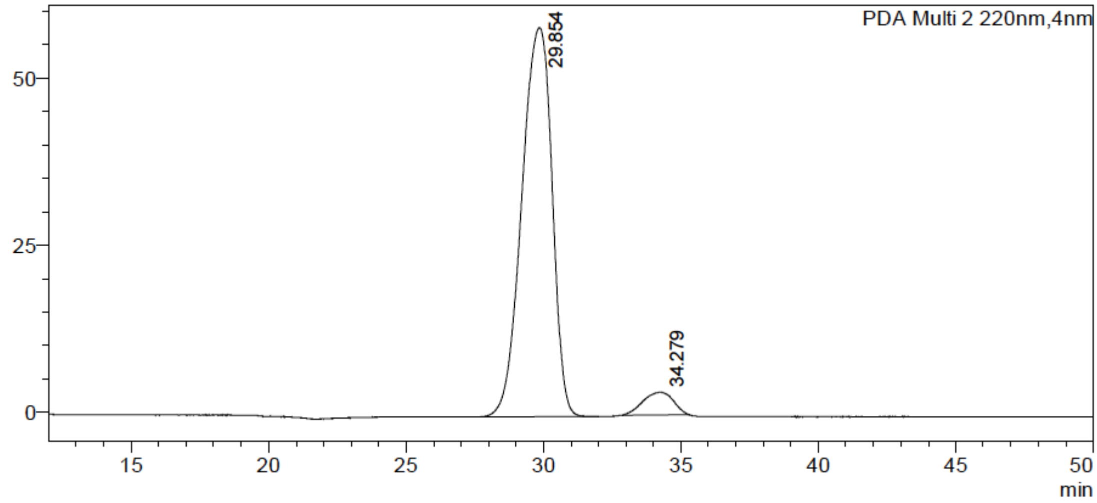

PDA Ch2 220nm

Peak#	Ret. Time	Area%
1	29.854	94.472
2	34.279	5.528
Total		100.000

HPLC data for **12**: Chiralpak OD-H (80:20 hexane:IPA, flow rate 1 mLmin^{-1}, 211 nm, 30 °C) t_R (3*S*): 18.2 min, t_R (3*R*): 26.3 min; 97% ee.

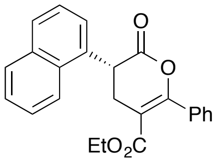

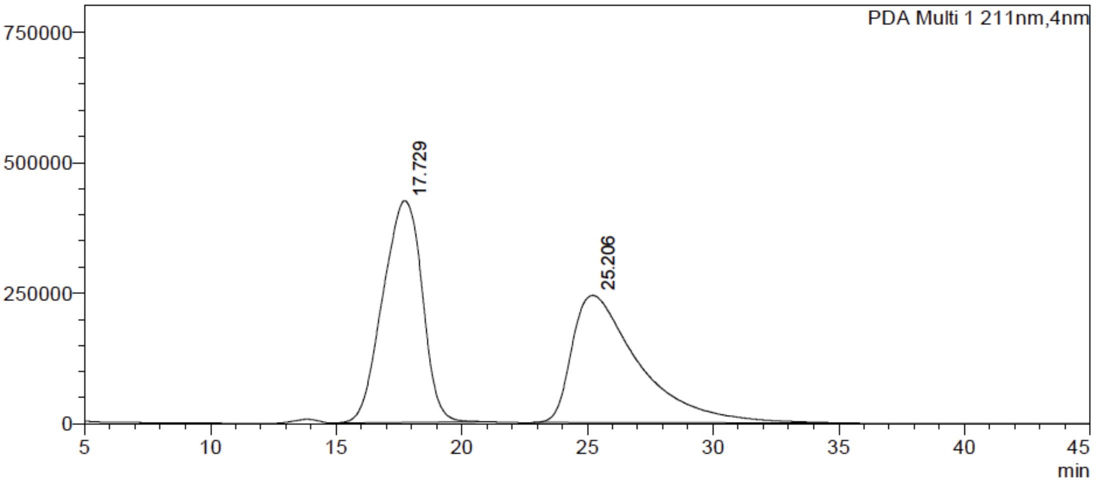

PDA Ch1 211nm

Peak#	Ret. Time	Area%
1	17.729	50.576
2	25.206	49.424
Total		100.000

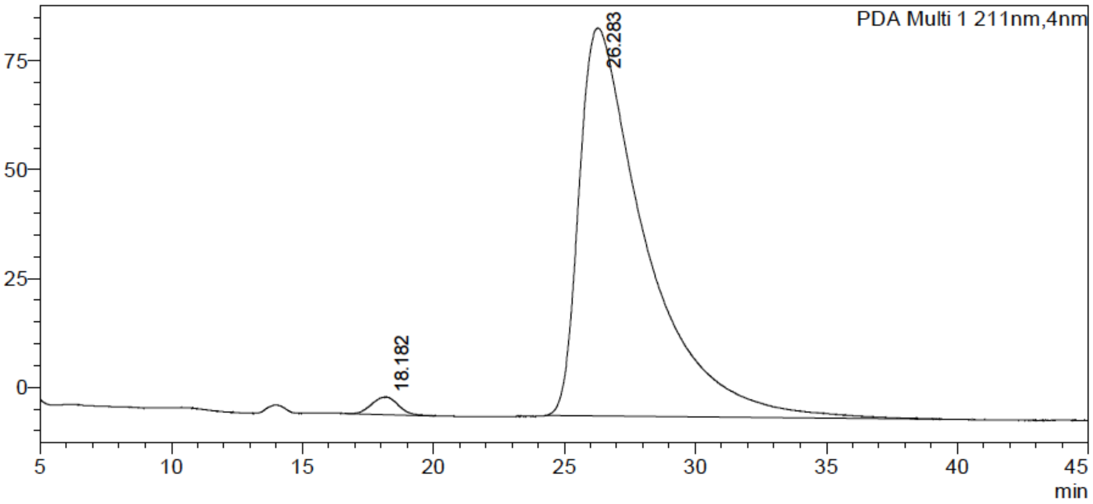

PDA Ch1 211nm

Peak#	Ret. Time	Area%
1	18.182	1.749
2	26.283	98.251
Total		100.000

HPLC data for **13**: Chiralpak OJ-H (70:30 hexane:IPA, flow rate 1 mLmin^{-1}, 220 nm, 30 °C) t_R (3*S*): 15.1 min, t_R (3*R*): 24.4 min; 50% ee.

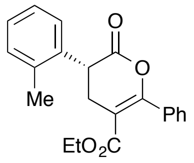

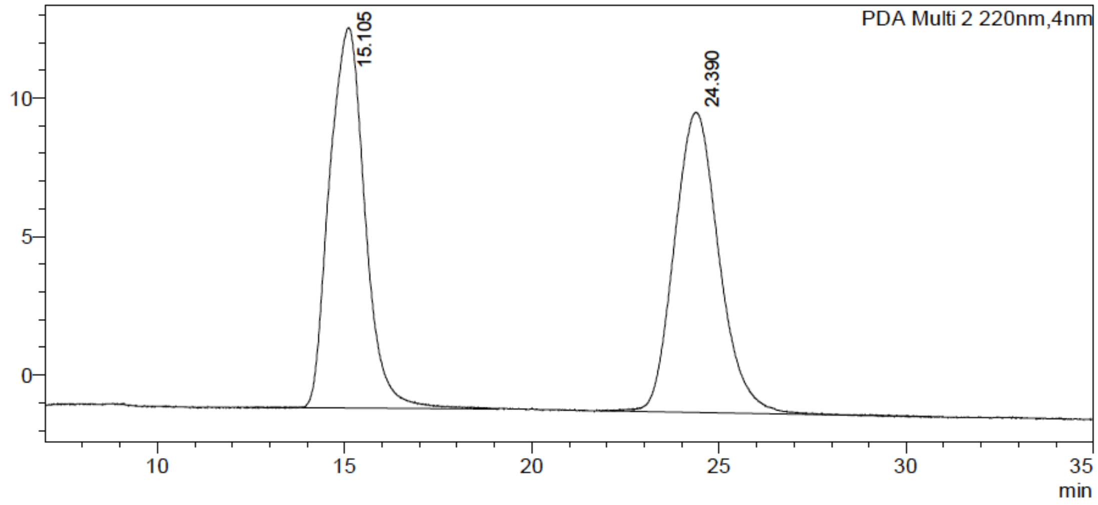

PDA Ch2 220nm

Peak#	Ret. Time	Area%
1	15.105	50.151
2	24.390	49.849
Total		100.000

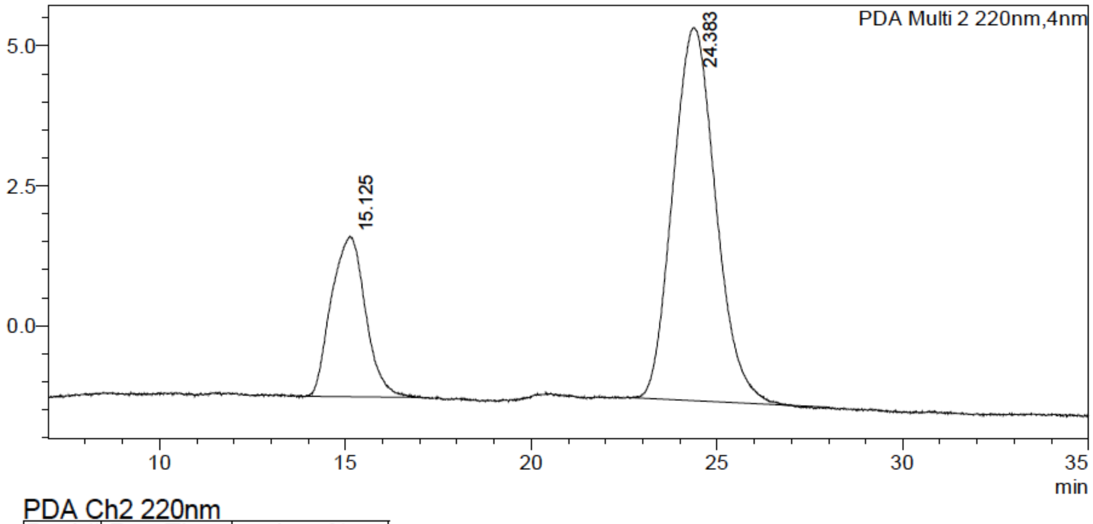

PDA Ch2 220nm

Peak#	Ret. Time	Area%
1	15.125	25.548
2	24.383	74.452
Total		100.000

HPLC data for **14**: Chiralpak AD-H (80:20 hexane:IPA, flow rate 1 mLmin^{-1}, 254 nm, 30 °C) t_R (3*S*): 12.1 min, t_R (3*R*): 16.0 min; 91% ee.

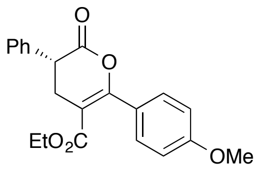

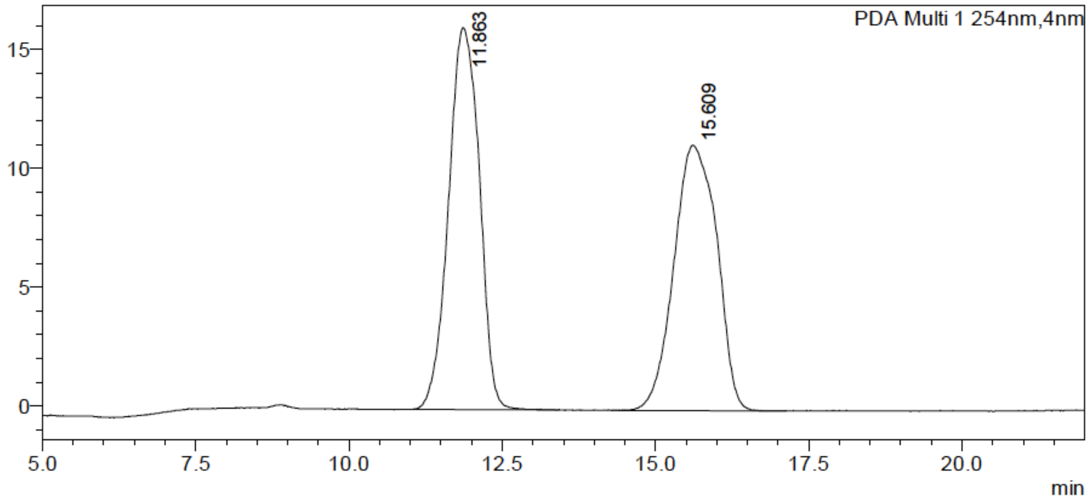

PDA Ch1 254nm

Peak#	Ret. Time	Area%
1	11.863	50.814
2	15.609	49.186
Total		100.000

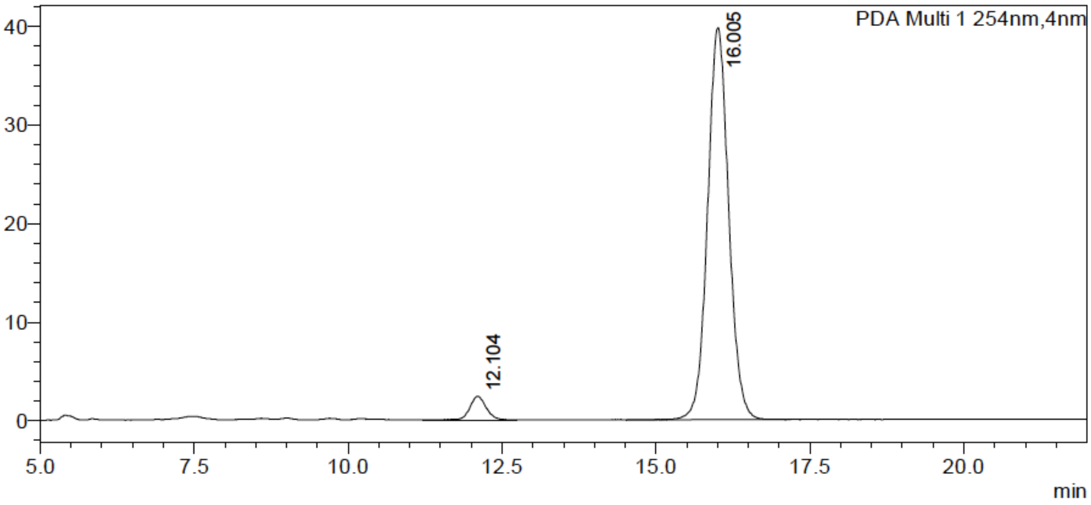

PDA Ch1 254nm

Peak#	Ret. Time	Area%
1	12.104	4.430
2	16.005	95.570
Total		100.000

HPLC data for **15**: Chiralpak AD-H (90:10 hexane:IPA, flow rate 1 mLmin^{-1}, 220 nm, 30 °C) t_R (3*S*): 15.3 min, t_R (3*R*): 18.8 min; 84% ee.

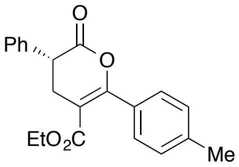

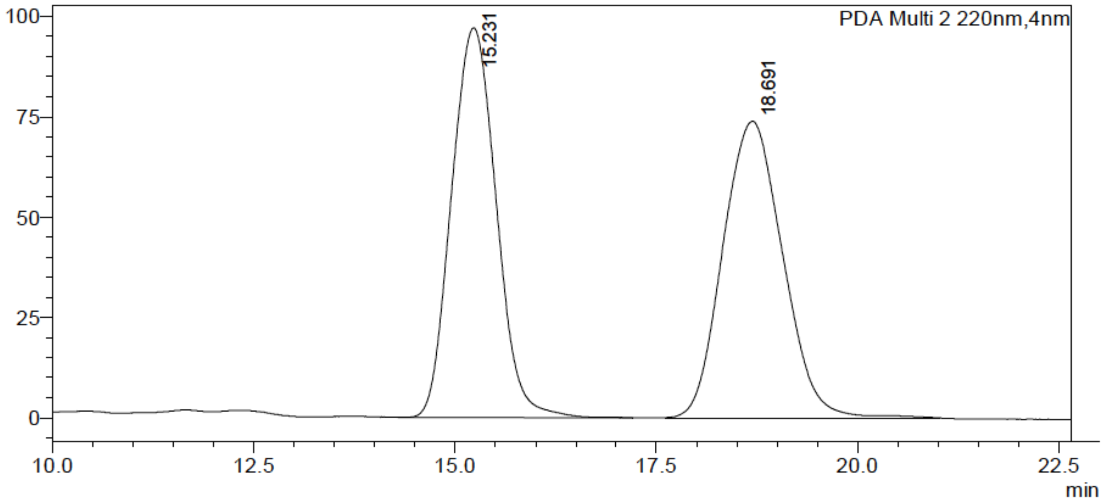

PDA Ch2 220nm

Peak#	Ret. Time	Area%
1	15.231	49.522
2	18.691	50.478
Total		100.000

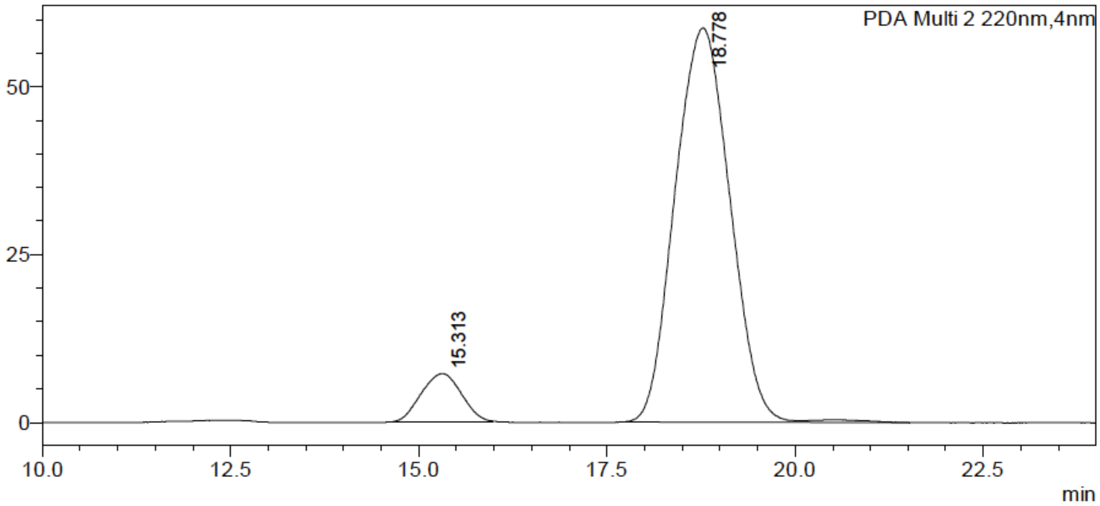

PDA Ch2 220nm

Peak#	Ret. Time	Area%
1	15.313	8.041
2	18.778	91.959
Total		100.000

HPLC data for **16**: Chiralpak OD-H (90:10 hexane:IPA, flow rate 1 mLmin^{-1}, 254 nm, 30 °C) t_R (3*S*): 18.4 min, t_R (3*R*): 26.2 min; 99% ee.

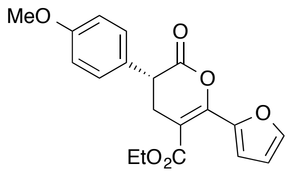

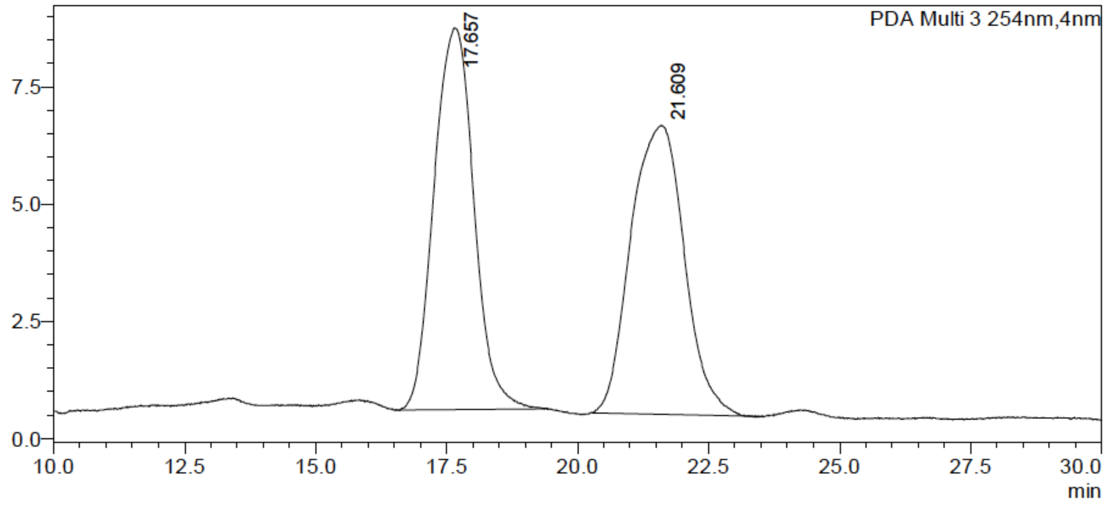

PDA Ch3 254nm

Peak#	Ret. Time	Area%
1	17.657	49.758
2	21.609	50.242
Total		100.000

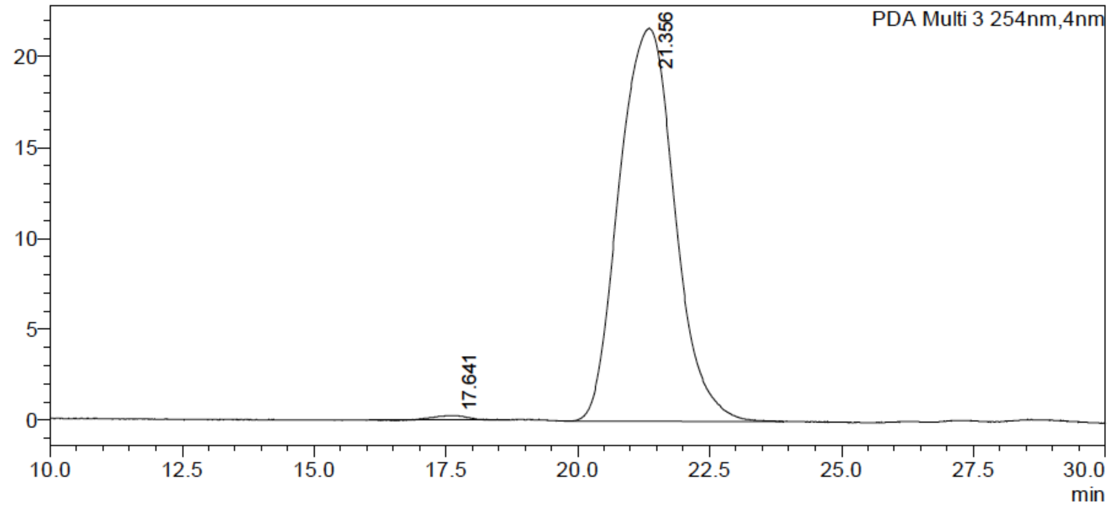

PDA Ch3 254nm

Peak#	Ret. Time	Area%
1	17.641	0.593
2	21.356	99.407
Total		100.000

HPLC data for **17**: Chiralpak OD-H (90:10 hexane:IPA, flow rate 1 mLmin^{-1}, 211 nm, 30 °C) t_R (3*R*): 18.4 min, t_R (3*S*): 26.2 min; 86% ee.

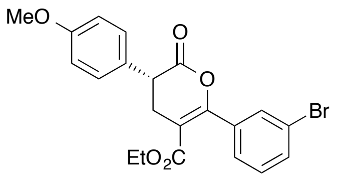

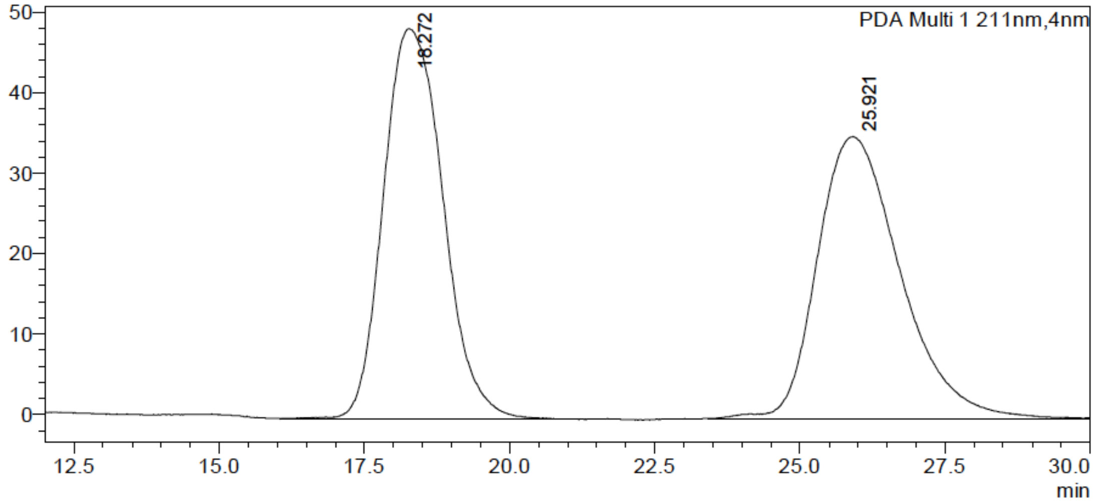

PDA Ch1 211nm

Peak#	Ret. Time	Area%
1	18.272	49.832
2	25.921	50.168
Total		100.000

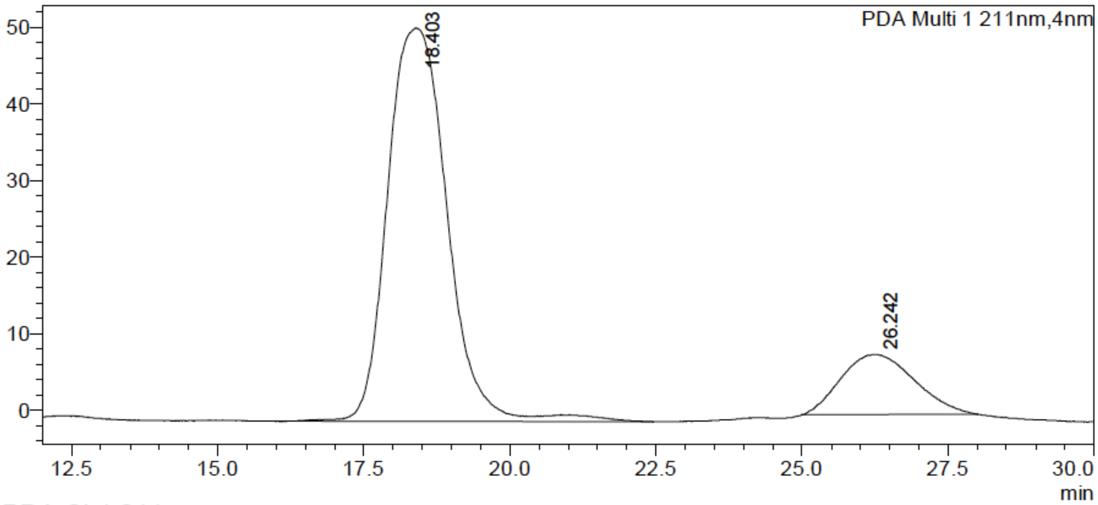

PDA Ch1 211nm

Peak#	Ret. Time	Area%
1	18.403	84.103
2	26.242	15.897
Total		100.000

HPLC data for **18**: Chiralpak OD-H (90:10 hexane:IPA, flow rate 1 mLmin^{-1}, 220 nm, 30 °C) t_R (3*S*): 24.1 min, t_R (3*R*): 28.2 min; 86% ee.

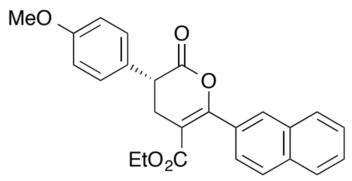

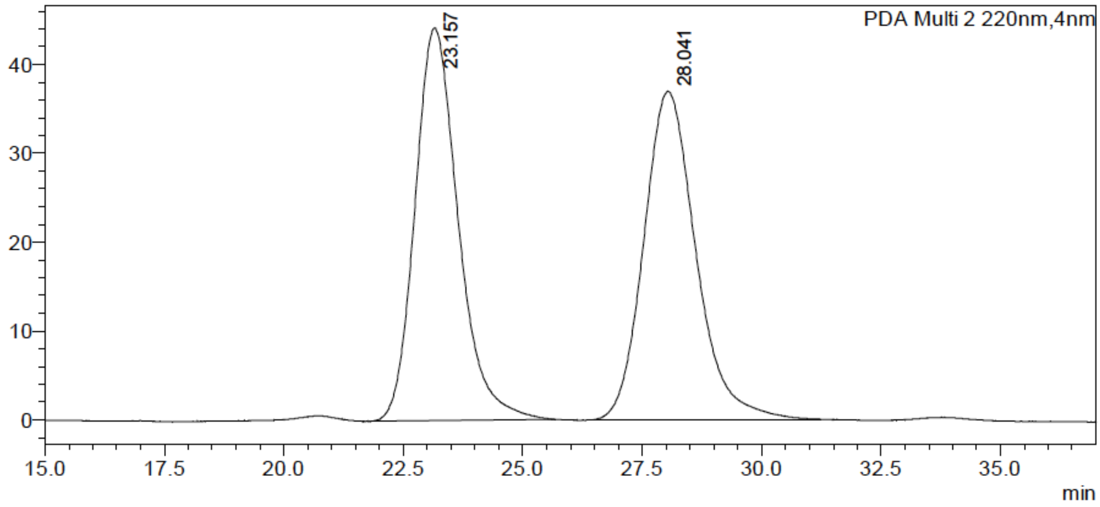

PDA Ch2 220nm

Peak#	Ret. Time	Area%
1	23.157	49.893
2	28.041	50.107
Total		100.000

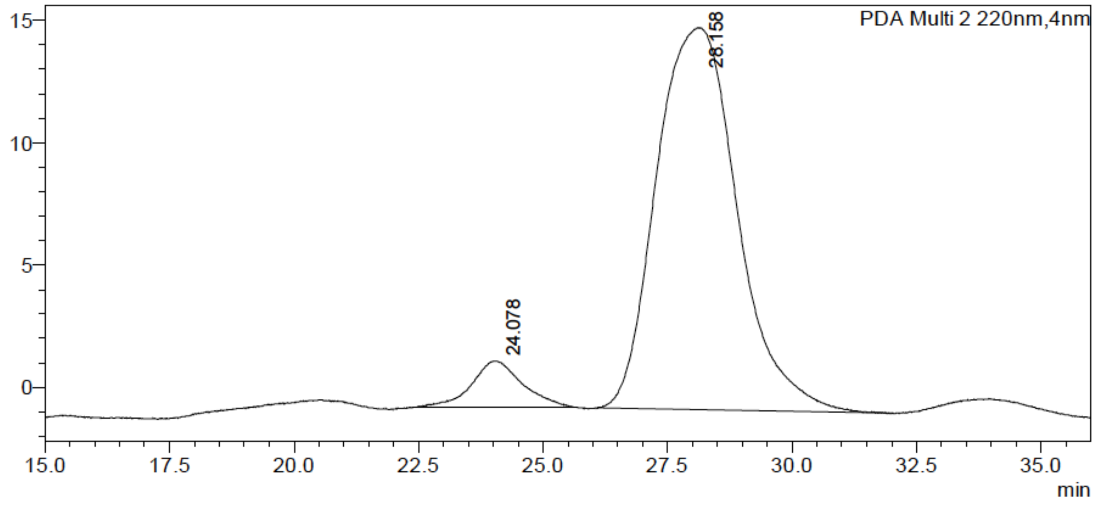

PDA Ch2 220nm

Peak#	Ret. Time	Area%
1	24.078	7.062
2	28.158	92.938
Total		100.000

HPLC data for **22**: Chiralpak AD-H (80:20 hexane:IPA, flow rate 1 mLmin^{-1}, 254 nm, 30 °C) t_R (3*S*): 15.8 min, t_R (3*R*): 23.0 min; 90% ee.

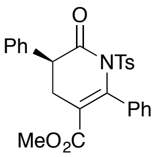

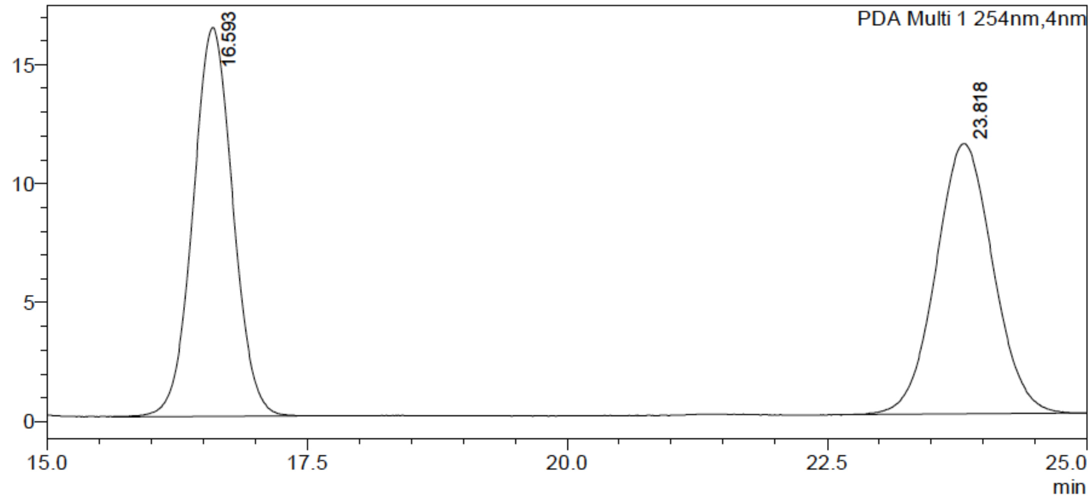

PDA Ch1 254nm

Peak#	Ret. Time	Area%
1	16.593	50.120
2	23.818	49.880
Total		100.000

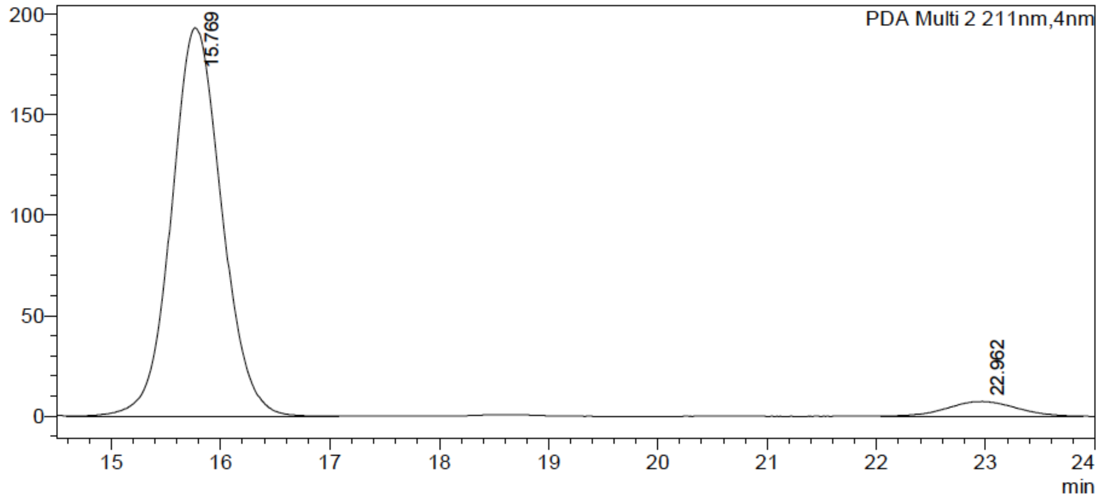

PDA Ch2 211nm

Peak#	Ret. Time	Area%
1	15.769	95.057
2	22.962	4.943
Total		100.000

HPLC data for **23**: Chiralpak OD-H (80:20 hexane:IPA, flow rate 1 mLmin^{-1}, 220 nm, 30 °C) t_R (3*S*): 12.7 min, t_R (3*R*): 25.4 min; 95% ee.

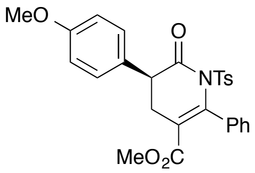

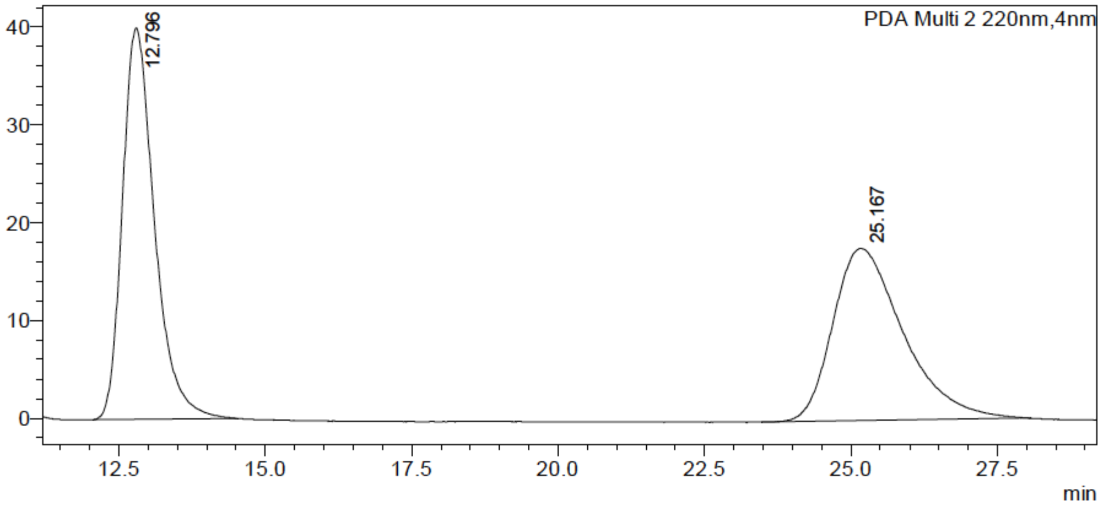

PDA Ch2 220nm

Peak#	Ret. Time	Area%
1	12.796	50.792
2	25.167	49.208
Total		100.000

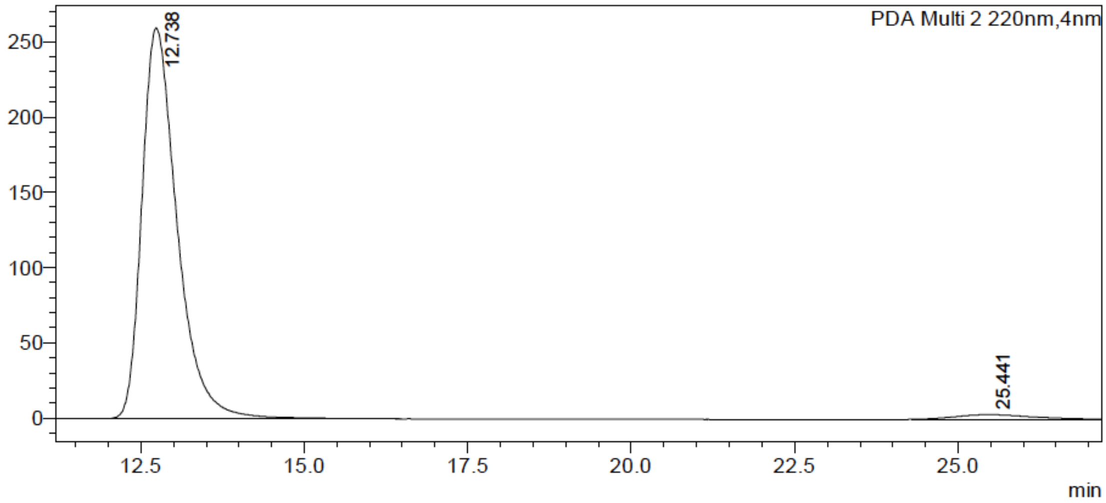

PDA Ch2 220nm

Peak#	Ret. Time	Area%
1	12.738	97.380
2	25.441	2.620
Total		100.000

HPLC data for **24**: Chiralpak AD-H (80:20 hexane:IPA, flow rate 1 mLmin^{-1}, 254 nm, 30 °C) t_R (3*R*): 23.4 min, t_R (3*S*): 26.2 min; 94% ee.

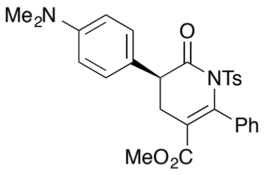

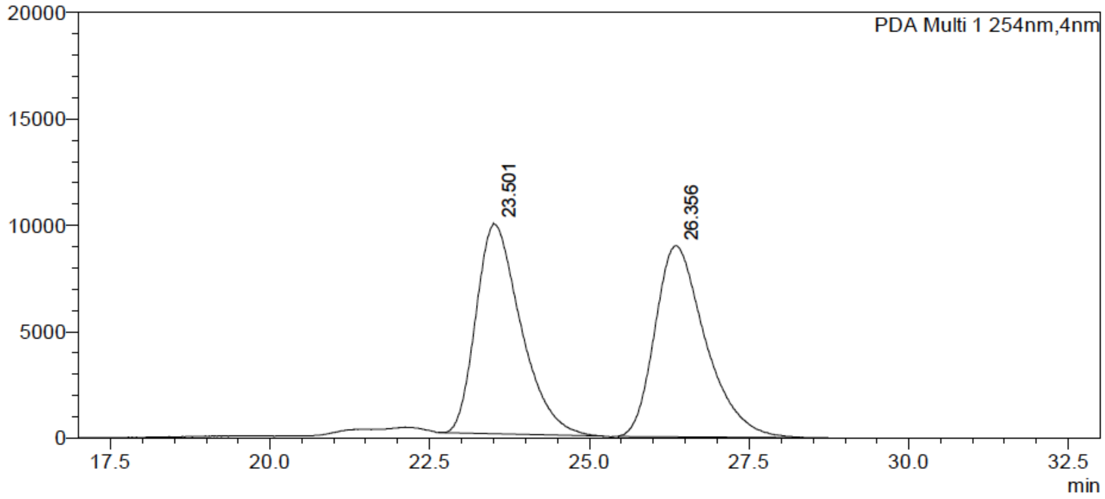

PDA Ch1 254nm

Peak#	Ret. Time	Area%
1	23.501	49.429
2	26.356	50.571
Total		100.000

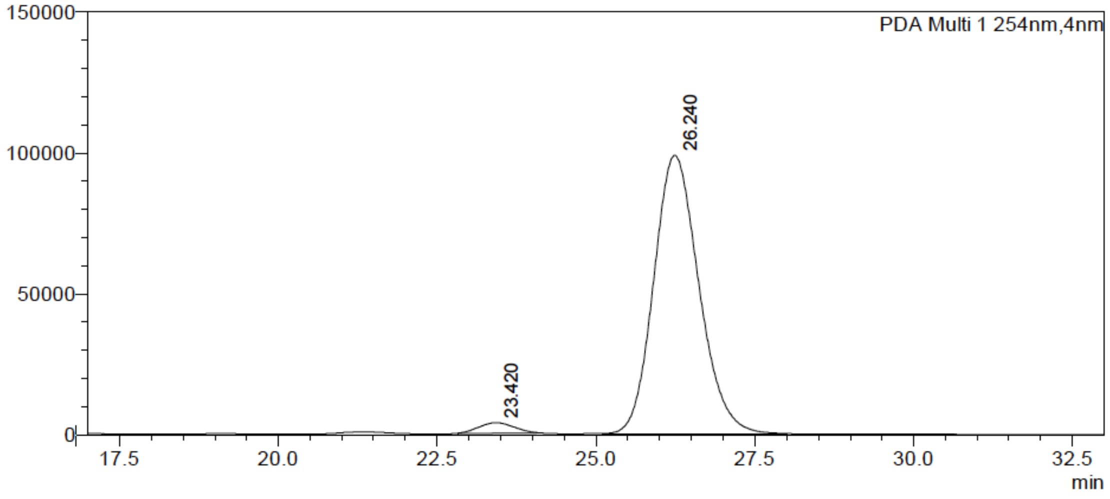

PDA Ch1 254nm

Peak#	Ret. Time	Area%
1	23.420	2.847
2	26.240	97.153
Total		100.000

HPLC data for **25**: Chiralpak AD-H (80:20 hexane:IPA, flow rate 1 mLmin^{-1}, 254 nm, 30 °C) t_R (3*R*): 17.2 min, t_R (3*S*): 21.5 min; 97% ee.

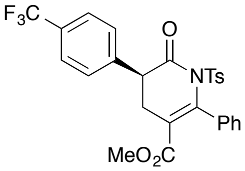

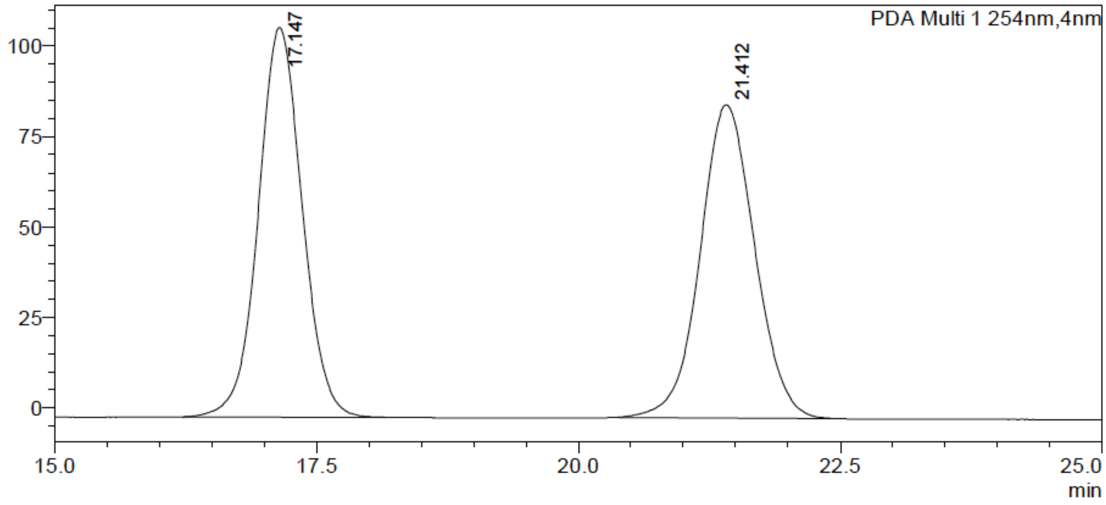

PDA Ch1 254nm

Peak#	Ret. Time	Area%
1	17.147	49.971
2	21.412	50.029
Total		100.000

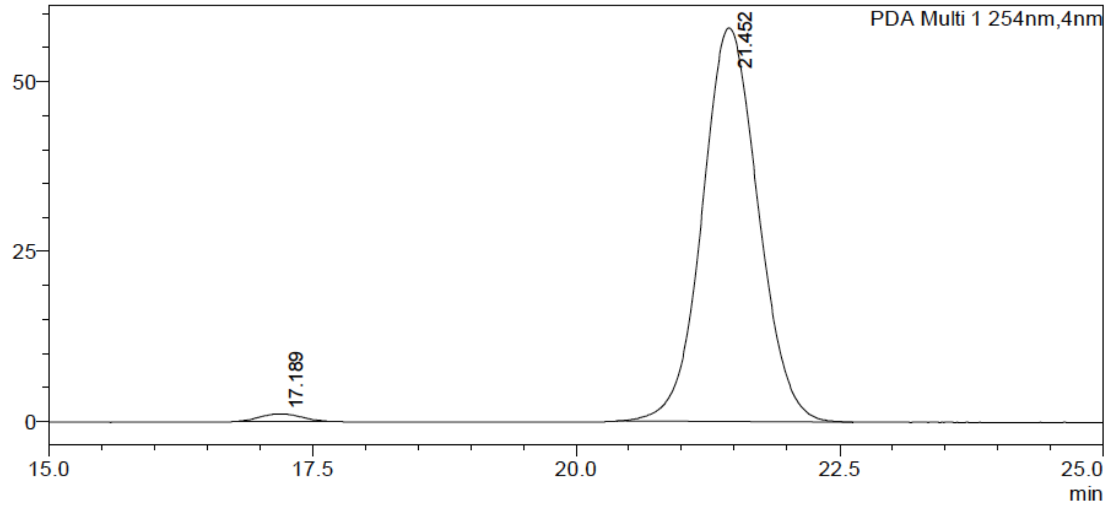

PDA Ch1 254nm

Peak#	Ret. Time	Area%
1	17.189	1.366
2	21.452	98.634
Total		100.000

HPLC data for **26**: Chiralpak AD-H (80:20 hexane:IPA, flow rate 1 mLmin^{-1}, 254 nm, 30 °C) t_R (3*S*): 14.7 min, t_R (3*R*): 28.7 min; 94% ee.

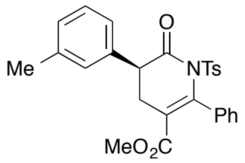

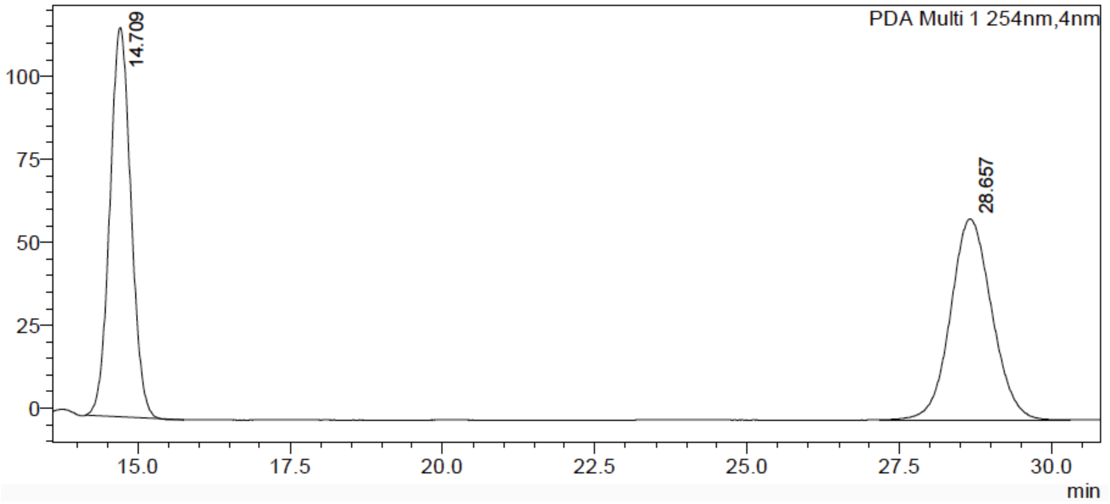

PDA Ch1 254nm

Peak#	Ret. Time	Area%
1	14.709	49.760
2	28.657	50.240
Total		100.000

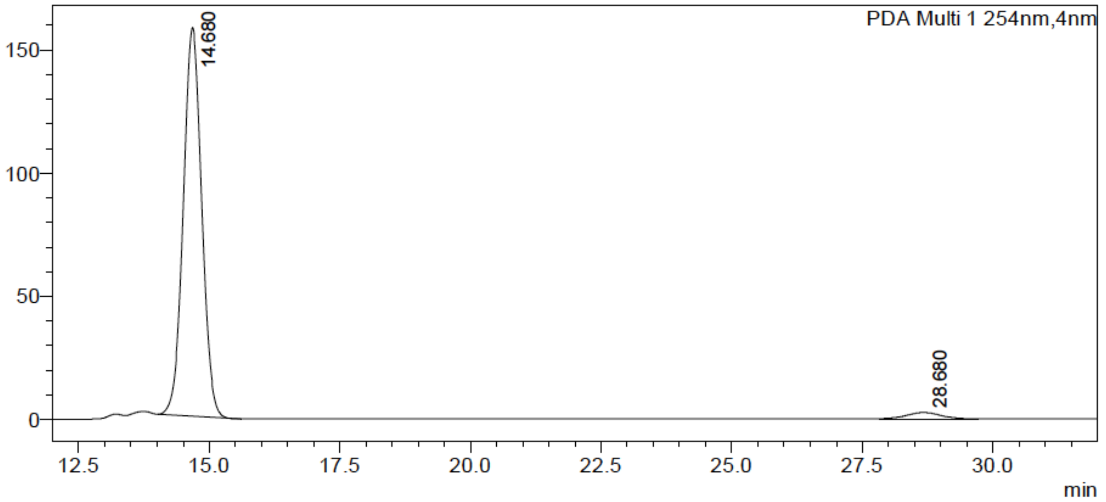

PDA Ch1 254nm

Peak#	Ret. Time	Area%
1	14.680	97.158
2	28.680	2.842
Total		100.000

HPLC data for **27**: Chiralpak AD-H (80:20 hexane:IPA, flow rate 1 mLmin^{-1}, 254 nm, 30 °C) t_R (3*R*): 23.3 min, t_R (3*S*): 28.3 min; 72% ee.

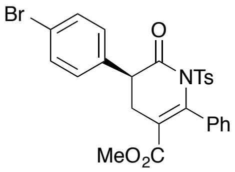

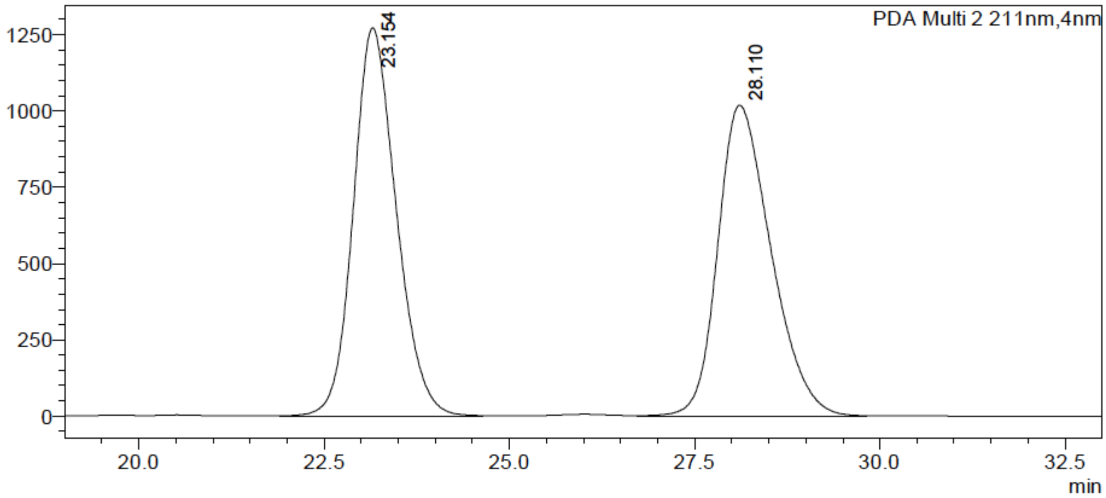

PDA Ch2 211nm

Peak#	Ret. Time	Area%
1	23.154	50.238
2	28.110	49.762
Total		100.000

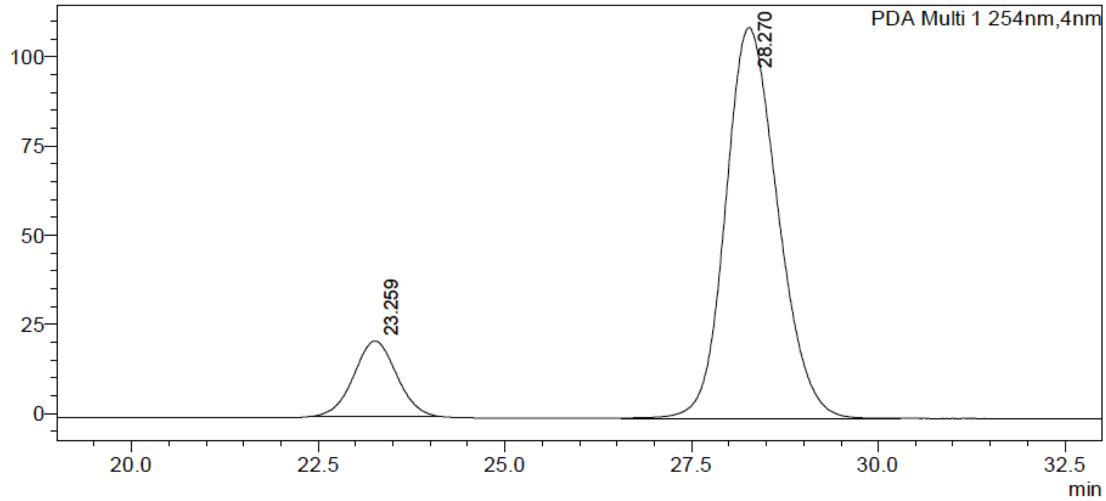

PDA Ch1 254nm

Peak#	Ret. Time	Area%
1	23.259	13.805
2	28.270	86.195
Total		100.000

HPLC data for **28**: Chiralpak IB (92.5:7.5 hexane:IPA, flow rate 1.5 mLmin^{-1}, 220 nm, 30 °C) t_R (3*S*): 13.9 min, t_R (3*R*): 15.8 min; 90% ee.

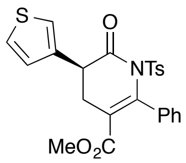

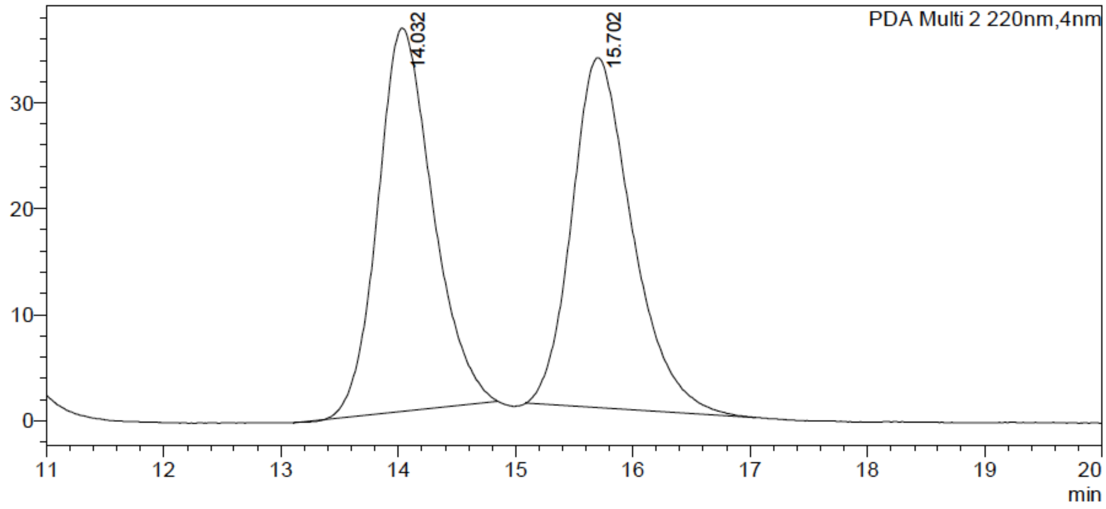

PDA Ch2 220nm

Peak#	Ret. Time	Area%
1	14.032	49.872
2	15.702	50.128
Total		100.000

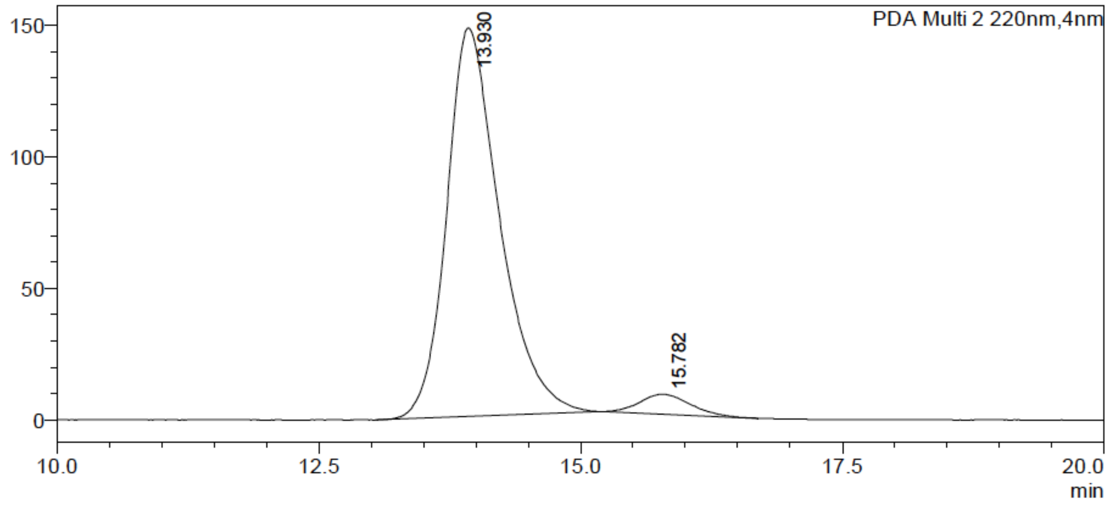

PDA Ch2 220nm

Peak#	Ret. Time	Area%
1	13.930	95.356
2	15.782	4.644
Total		100.000

HPLC data for **29**: Chiralpak OD-H (80:20 hexane:IPA, flow rate 1 mLmin^{-1}, 254 nm, 30 °C) t_R (3*S*): 15.5 min, t_R (3*R*): 25.8 min; 98% ee.

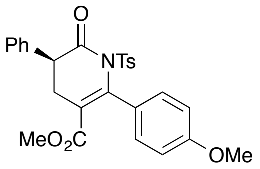

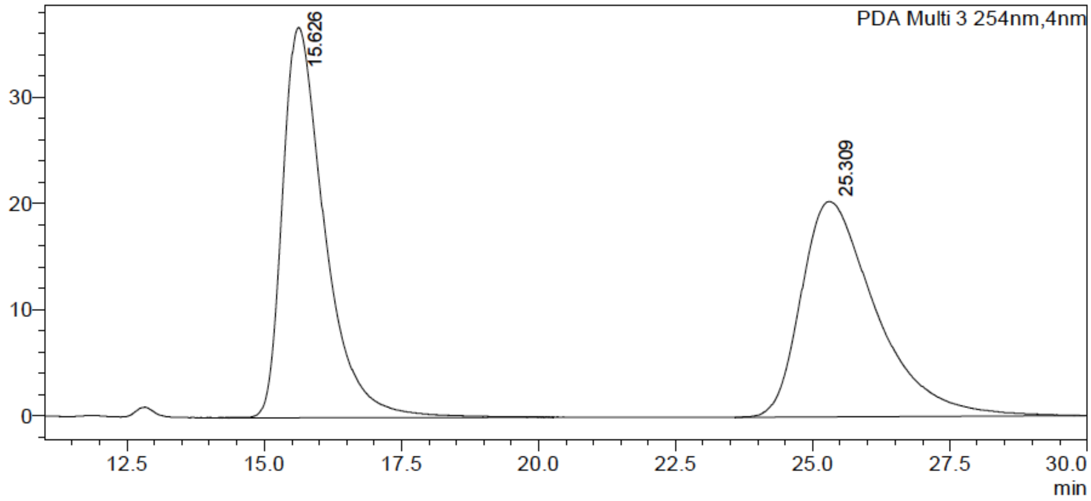

PDA Ch3 254nm

Peak#	Ret. Time	Area%
1	15.626	50.466
2	25.309	49.534
Total		100.000

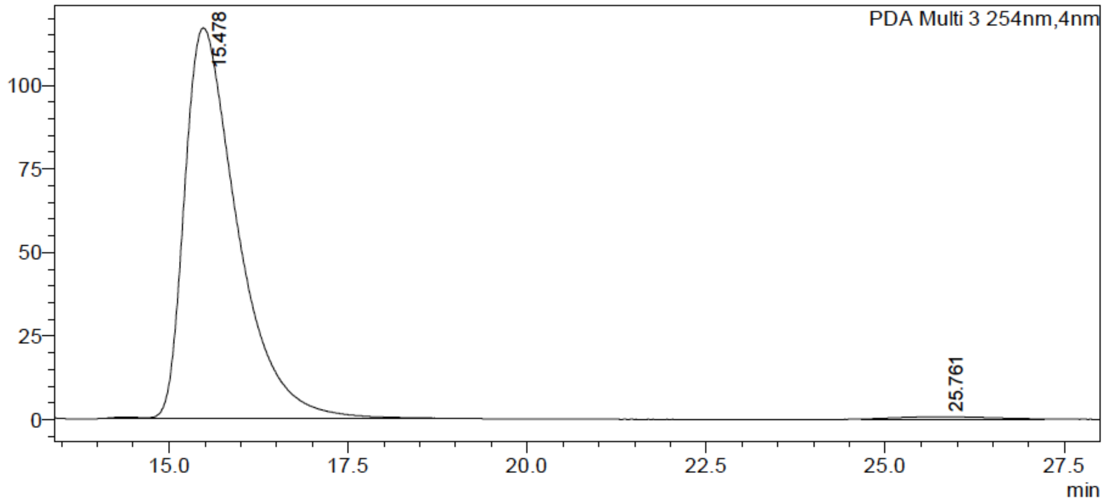

PDA Ch3 254nm

Peak#	Ret. Time	Area%
1	15.478	99.036
2	25.761	0.964
Total		100.000

HPLC data for **30**: Chiralpak AD-H (80:20 hexane:IPA, flow rate 1 mLmin^{-1}, 211 nm, 30 °C) t_R (3*S*): 13.3 min, t_R (3*R*): 25.6 min; 90% ee.

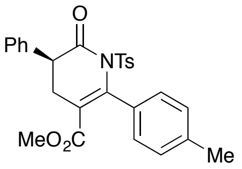

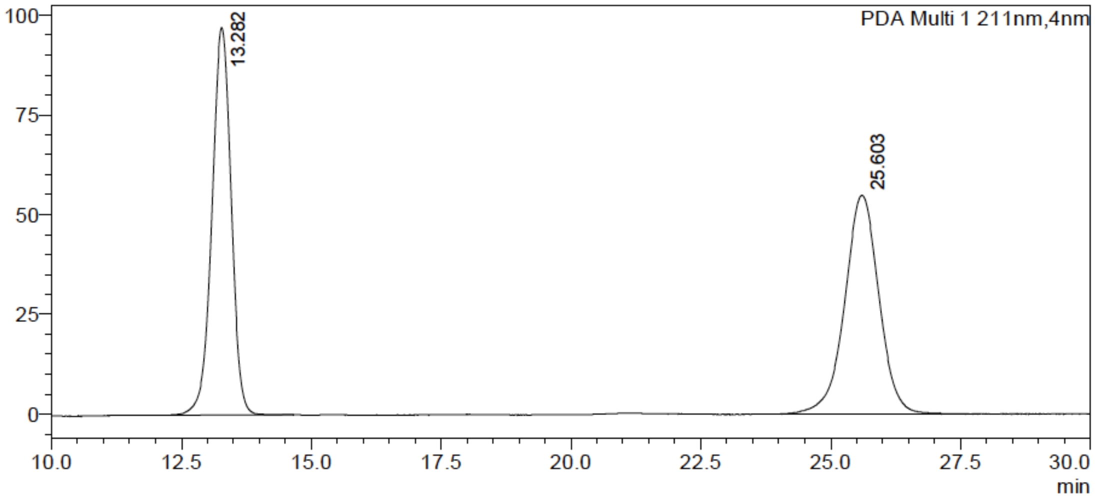

PDA Ch1 211nm

Peak#	Ret. Time	Area%
1	13.282	49.949
2	25.603	50.051
Total		100.000

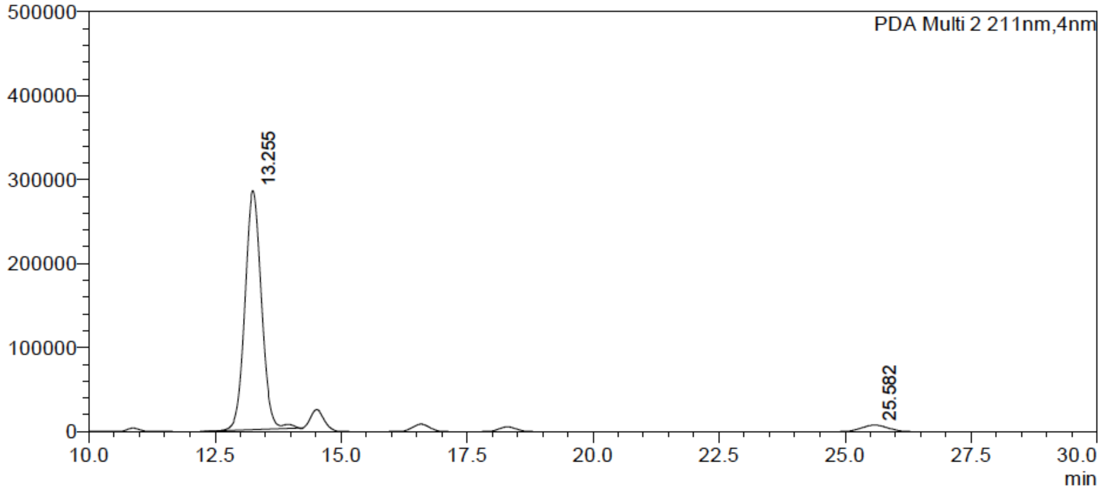

PDA Ch2 211nm

Peak#	Ret. Time	Area%
1	13.255	94.834
2	25.582	5.166
Total		100.000

HPLC data for **31**: Chiralpak AD-H (90:10 hexane:IPA, flow rate 1 mLmin^{-1}, 211 nm, 30 °C) t_R (3*S*): 11.8 min, t_R (3*R*): 17.6 min; 97% ee.

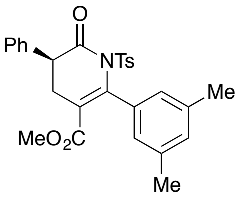

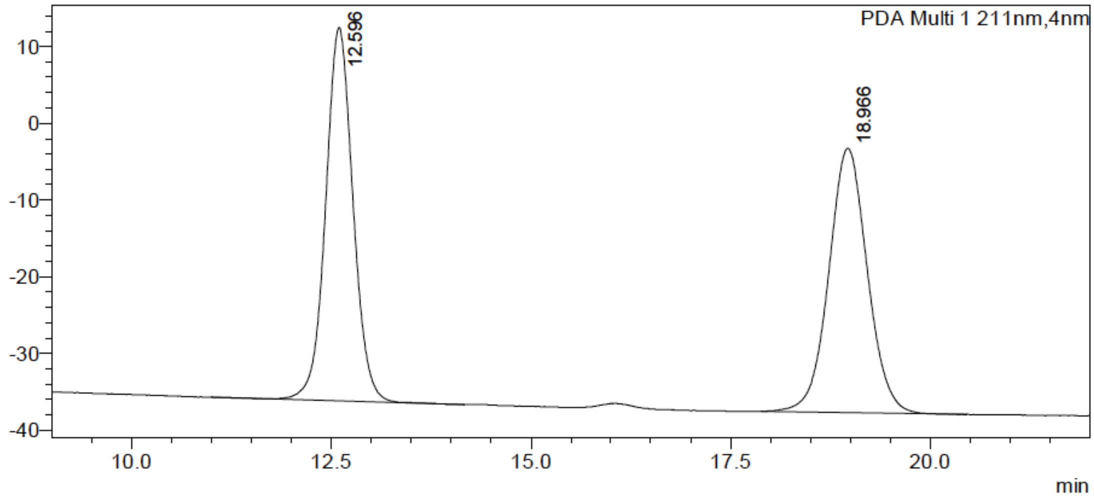

PDA Ch1 211nm

Peak#	Ret. Time	Area%
1	12.596	50.116
2	18.966	49.884
Total		100.000

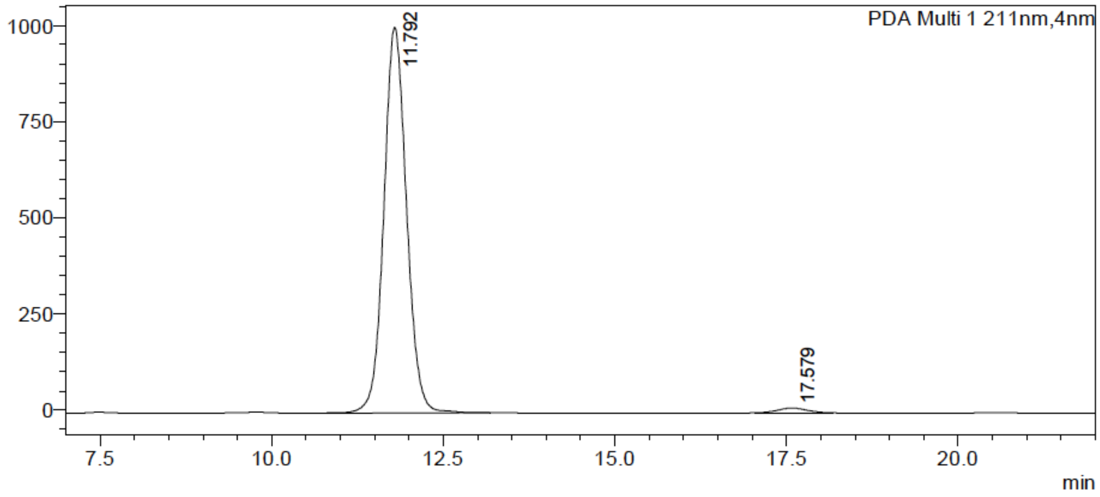

PDA Ch1 211nm

Peak#	Ret. Time	Area%
1	11.792	98.397
2	17.579	1.603
Total		100.000

HPLC data for **32**: Chiralpak AD-H (80:20 hexane:IPA, flow rate 1 mLmin^{-1}, 254 nm, 30 °C) t_R (3*S*): 15.3 min, t_R (3*R*): 23.7 min; 97% ee.

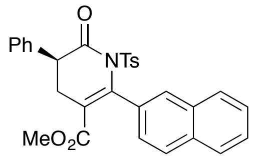

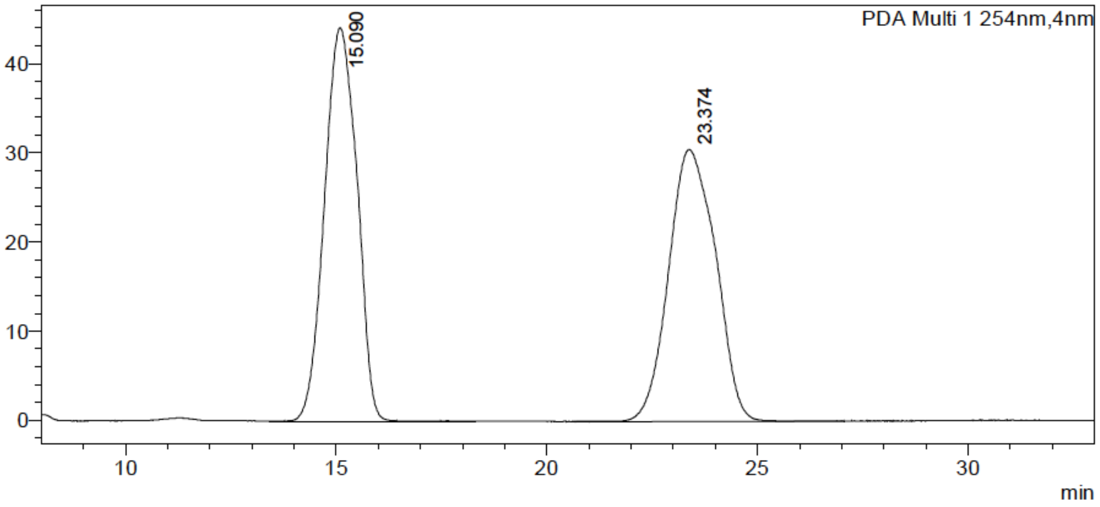

PDA Ch1 254nm

Peak#	Ret. Time	Area%
1	15.090	50.043
2	23.374	49.957
Total		100.000

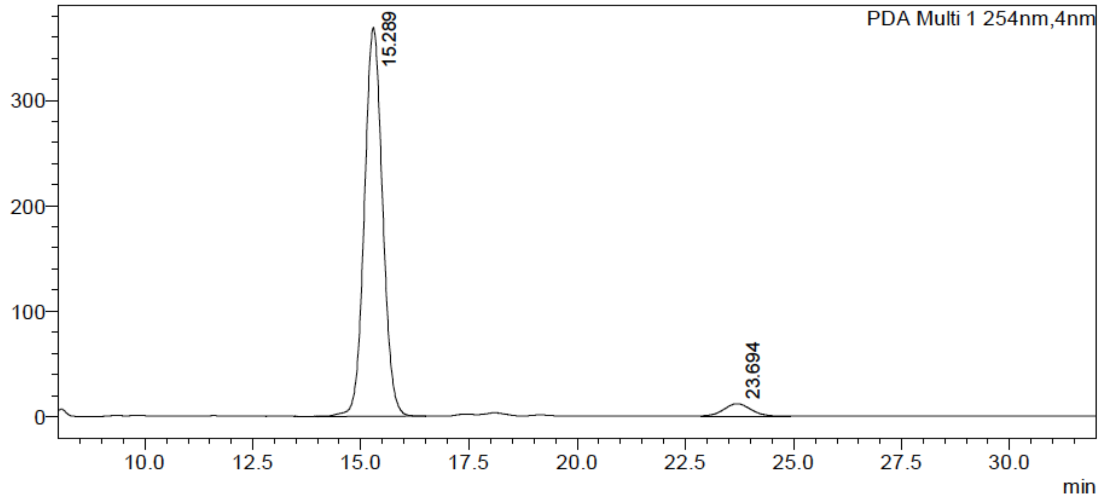

PDA Ch1 254nm

Peak#	Ret. Time	Area%
1	15.289	95.592
2	23.694	4.408
Total		100.000
